# Evolution of Genome Size and Complexity in the *Rhabdoviridae*

**DOI:** 10.1371/journal.ppat.1004664

**Published:** 2015-02-13

**Authors:** Peter J. Walker, Cadhla Firth, Steven G. Widen, Kim R. Blasdell, Hilda Guzman, Thomas G. Wood, Prasad N. Paradkar, Edward C. Holmes, Robert B. Tesh, Nikos Vasilakis

**Affiliations:** 1 CSIRO Biosecurity, Australian Animal Health Laboratory, Geelong, Victoria, Australia; 2 Department of Biochemistry and Molecular Biology, The University of Texas Medical Branch, Galveston, Texas, United States of America; 3 Center for Biodefense and Emerging Infectious Diseases and Department of Pathology, Center for Tropical Diseases, and Institute for Human Infections and Immunity, The University of Texas Medical Branch, Galveston, Texas, United States of America; 4 Marie Bashir Institute for Infectious Diseases and Biosecurity, Charles Perkins Centre, School of Biological Sciences and Sydney Medical School, The University of Sydney, Sydney, New South Wales, Australia; Leiden University Medical Center, NETHERLANDS

## Abstract

RNA viruses exhibit substantial structural, ecological and genomic diversity. However, genome size in RNA viruses is likely limited by a high mutation rate, resulting in the evolution of various mechanisms to increase complexity while minimising genome expansion. Here we conduct a large-scale analysis of the genome sequences of 99 animal rhabdoviruses, including 45 genomes which we determined *de novo*, to identify patterns of genome expansion and the evolution of genome complexity. All but seven of the rhabdoviruses clustered into 17 well-supported monophyletic groups, of which eight corresponded to established genera, seven were assigned as new genera, and two were taxonomically ambiguous. We show that the acquisition and loss of new genes appears to have been a central theme of rhabdovirus evolution, and has been associated with the appearance of alternative, overlapping and consecutive ORFs within the major structural protein genes, and the insertion and loss of additional ORFs in each gene junction in a clade-specific manner. Changes in the lengths of gene junctions accounted for as much as 48.5% of the variation in genome size from the smallest to the largest genome, and the frequency with which new ORFs were observed increased in the 3’ to 5’ direction along the genome. We also identify several new families of accessory genes encoded in these regions, and show that non-canonical expression strategies involving TURBS-like termination-reinitiation, ribosomal frame-shifts and leaky ribosomal scanning appear to be common. We conclude that rhabdoviruses have an unusual capacity for genomic plasticity that may be linked to their discontinuous transcription strategy from the negative-sense single-stranded RNA genome, and propose a model that accounts for the regular occurrence of genome expansion and contraction throughout the evolution of the *Rhabdoviridae*.

## Author Summary

Understanding the patterns and mechanisms of genome evolution is one of the most important, yet least understood, aspects of RNA virus biology. The evolutionary challenge faced by RNA viruses is to maximize functional diversity within severe constraints on genome size. Here we show that rhabdoviruses, a family of RNA viruses that infect hosts as diverse as plants, insects and vertebrates, have an unusual capacity for genomic plasticity. By analysing the complete or near-complete genome sequences of 99 animal rhabdoviruses, we show that genome expansion and contraction has likely occurred frequently throughout the evolution of the family. Genomic plasticity has been associated with the evolution of alternative, overlapping and consecutive ORFs within the major structural protein genes, as well as the insertion and loss of additional ORFs in each gene junction in a clade-specific manner. This has resulted in remarkable diversity in genome organisation and gene expression strategies that is reflective of the broad ecological diversity of rhabdoviruses. We conclude that genomic plasticity in rhabdoviruses may be linked to their discontinuous transcription strategy from the negative-sense single-stranded RNA genome and propose a general model that accounts for both gains and losses in genome size and complexity.

## Introduction

RNA viruses are among the most structurally and ecologically diverse of all life forms [1]. Their genomes may consist of positive (+) sense, negative (-) sense or ambi-sense single-stranded (ss) RNA, or double-stranded (ds) RNA, and may take the form of a single or multiple segments that are packaged in single or multiple particles. RNA viruses also employ a plethora of strategies for replication and gene expression, and encode a vast array of structural and non-structural proteins, many of which are unique and have multiple, highly specialized functions [2]. Despite their diversity, RNA virus genomes are ubiquitously small, averaging only 10 kb, and with a maximum size of ~32 kb for some members of the order *Nidovirales* [3,4]. This size limitation has been linked to high mutation rates (a mean rate of ~1 mutation /genome /replication) due to replication with an error-prone RNA-dependent RNA polymerase that lacks proofreading capability [5,6]. High error rates are thought to limit genome sizes because, as size increases, the number of deleterious mutations also increases to levels beyond which reproduction of the fittest variant cannot be guaranteed [7,8].

Due to this fundamental evolutionary constraint, RNA viruses have employed various mechanisms of genome compression, such as the use of alternative or overlapping open reading frames (ORFs) and the evolution of multiple functions for individual proteins [4,7,9]. For some RNA viruses, increases in genome size have been associated with increases in the size of replicative proteins [10] and the presence of helicase and proof-reading exonuclease domains [3,11–13]. However, the mechanisms and evolutionary context that would favour increased genome size and complexity, given constraints on replication efficiency, are currently unknown [3,4].

The *Rhabdoviridae* is one of the most ecologically diverse families of RNA viruses. Rhabdoviruses have been identified in a very wide range of plants and animals, including mammals, birds, reptiles, and fish with many transmitted by arthropod vectors [14,15]. The family includes rabies virus (RABV), which causes over 25,000 human deaths annually [16], vesicular stomatitis Indiana virus (VSIV), which has served as an important model for the study of many aspects of mammalian virus replication and virus-host interactions, and many other important pathogens of humans, livestock, farmed aquatic animals and food crops. The non-segmented [–] ssRNA rhabdovirus genome is packaged within a characteristic bullet- or rod-shaped particle comprising five structural proteins—the nucleoprotein (N), polymerase-associated phosphoprotein (P), matrix protein (M), glycoprotein (G) and RNA-dependent RNA polymerase (L) [17]. The genome features partially complementary, untranslated leader (*l*) and trailer (*t*) sequences and five ORFs arranged in the order 3’-N-P-M-G-L-5’. Each ORF is flanked by relatively conserved transcription initiation (TI) and transcription termination/polyadenylation (TTP) sequences which orchestrate expression of the five corresponding capped and polyadenylated mRNAs [17]. Rhabdovirus genomes may also contain additional ORFs encoding putative proteins, which are mostly of unknown function. These may occur as alternative or overlapping ORFs within the major structural protein genes or as independent ORFs flanked by TI or TTP sequences in the regions between the structural protein genes [15], some of which appear to have arisen by gene duplication [15,18–22].

Here we undertake the first large-scale analysis of the evolution of genome size and complexity in a family of [–] ssRNA viruses. We demonstrate that remarkable changes in genome size and complexity have occurred in rhabdoviruses in a clade-specific manner, primarily by extension and insertion of additional transcriptional units in the structural protein gene junctions, followed by occasional losses. We also show that rhabdoviruses have evolved a large number of accessory proteins and that the use of non-canonical gene expression strategies appears to be common, particularly amongst vector-borne rhabdoviruses.

## Results

### Genome sequences and sequence annotation

**Fig 1:**
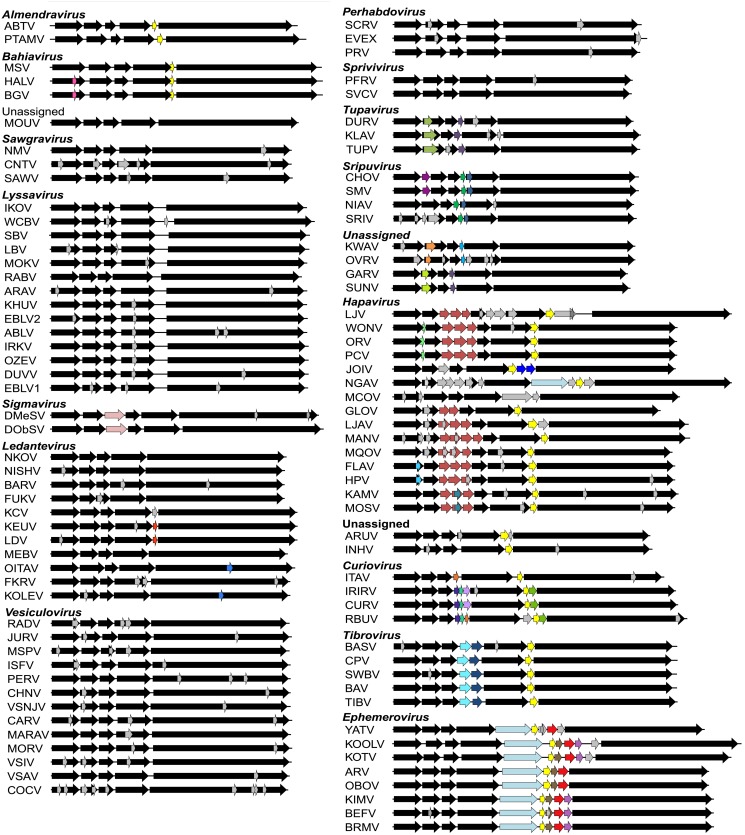
Schematic representation of the genomes of rhabdoviruses analysed. The genomes are shown in (+) sense with arrows indicating the locations of ORFs ≥180 nt. The five common structural protein genes (N, P, M, G and L) are shaded in black. Orthologous genes or genes encoding structurally similar proteins are shaded in the same colour, including viroporin-like proteins which are shaded in yellow. ORFs for which no orthologous or structurally similar proteins could be identified are shaded in light grey. The viruses are grouped according to established genera, proposed new genera or unassigned species (see Fig. 2).

Our data set comprised the complete or near-complete genome sequences of 99 animal rhabdoviruses, including 45 viruses isolated from various vertebrates and arthropods for which we determined the sequences *de novo* (S1 Table). Incomplete genomes lacked only the extreme terminal sequences. All rhabdovirus genomes contained the five canonical structural protein genes (N, P, M, G and L); however, there was remarkable diversity in the number and location of other long ORFs. Across the data set, we identified 179 additional ORFs ≥180 nt in length of which 142 shared no detectable protein sequence similarity with any other protein in our data set or with those in public databases (S2 Table). These additional ORFs were located either within the structural protein genes or in additional transcriptional units located in regions between these genes (Fig. 1). The additional transcriptional units were annotated by using relatively conserved TI and TTP motifs. The core TI sequence (UUGU) was conserved with some minor variations (CUGU, UUGC, UUGA, UCGU, UGAU) employed in some viruses. The TTP motif G[U]_7_ was also conserved, with the variation A[U]_7_ occurring only in several genes of one virus (CHOV).

**Fig 2:**
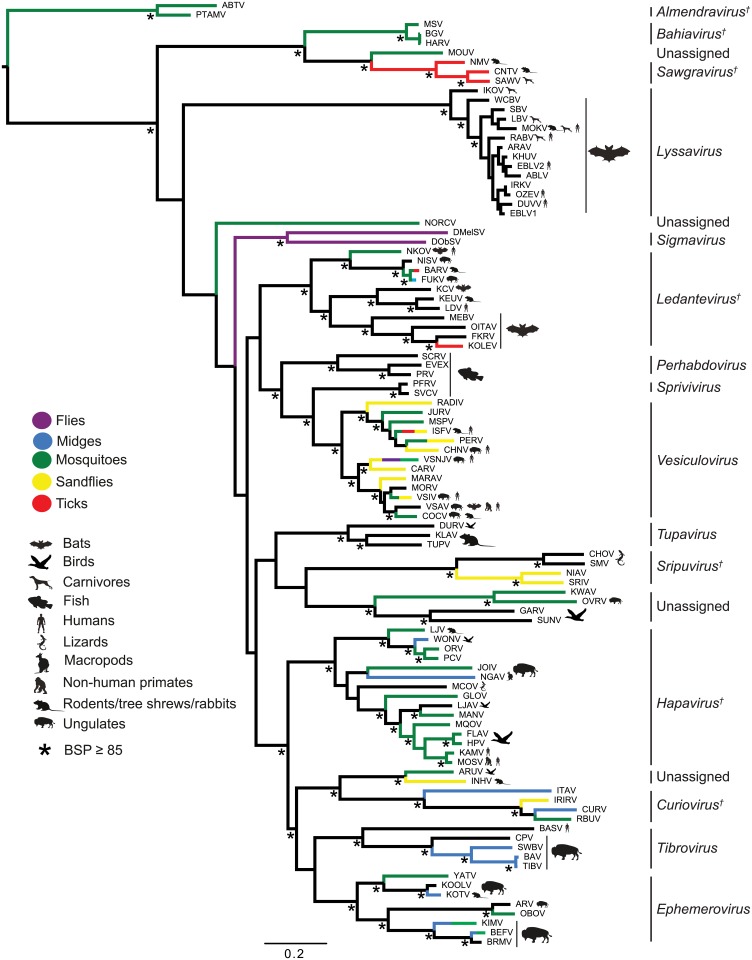
ML phylogenetic tree of 100 rhabdovirus L protein sequences. Branches are colour-coded according to known vector species, while the principal animal host species (where known) are shown by indicated symbols. Horizontal branch lengths are drawn to a scale of amino acid substitutions/site, and all bootstrap proportion values (BSP) ≥ 85% are shown by the * symbol. Newly proposed genera are indicated by a † symbol. Cytorhabdovirus, novirhabdovirus and nucleorhabdovirus outgroup sequences were excluded from the tree as they were too divergent to establish a reliable rooting. The tree is therefore rooted arbitrarily on one of two basal clades (genera *Almendravirus* and *Bahiavirus*) that comprise viruses isolated from mosquitoes.

Due to the large number and diversity of additional ORFs, we adopted a standard nomenclature that does not necessarily reflect structural homology. Unless previously assigned a distinctive name (e.g., BEFV G_NS_, α1, α2, β and γ proteins), all ORFs ≥180 nt were assigned names according to the following rules: i) each additional transcriptional unit was designated U (unknown) followed by a number as they appeared in order in the genome presented in positive polarity (i.e., U1, U2, U3, etc); ii) the first ORF within each transcriptional unit was assigned the same designation as the transcriptional unit; and iii) each subsequent ORF within any transcriptional unit (alternative, overlapping or consecutive) was designated by letter (i.e., U1x, U1y, U1z) (S2 Table). Alternative ORFs are defined here as those which occur in a different frame within another longer ORF; overlapping ORFs are alternative ORFs which extend beyond the end of the primary ORF; and consecutive ORFs are those which do not overlap but follow consecutively within the same transcriptional unit. The arbitrary cut-off of ≥180 nt (≥60 aa) was selected on the basis that two small basic proteins of 55 and 65 amino acids (C and C’) have been shown to be expressed from an alternative ORF within the VSIV P gene [23,24]. These are the smallest known rhabdovirus proteins.

### Phylogenetic relationships and proposed taxonomic assignments

To determine the evolutionary history of the rhabdoviruses studied here, we inferred a phylogenetic tree using conserved regions of the L protein of all 99 viruses in our data set as well as the recently described North Creek virus (NORCV) [25,26] (Fig. 2). All but two of these 100 rhabdoviruses (NORCV and MOUV) clustered into 17 well-supported monophyletic groups (bootstrap proportion [BSP] ≥ 85); however, many of the deeper nodes were unresolved throughout the phylogeny. Eight of the well-supported clades corresponded to the eight established genera (*Lyssavirus*, *Vesiculovirus*, *Perhabdovirus*, *Sigmavirus*, *Ephemerovirus*, *Tibrovirus*, *Tupavirus* and *Sprivivirus)* and we assigned a further seven clades as proposed new genera (*Almendravirus*, *Bahiavirus*, *Curiovirus*, *Hapavirus*, *Ledantevirus*, *Sawgravirus* and *Sripuvirus*). The taxonomic assignment of the two remaining clades was considered to be ambiguous (S1 Table). For simplicity of expression we refer here to all as ‘genera’, whether existing or proposed, but we recognise that taxonomic proposals require consideration and ratification by the International Committee on Taxonomy of Viruses (ICTV).

Although the analysis was limited by the availability of single isolates of most viruses, apparent structure by geographic location or reservoir host was not observed in the phylogeny. However, multiple genera appeared to be primarily associated with bats (i.e., ledanteviruses, lyssaviruses), fish (i.e., perhabdoviruses, spriviviruses) or ungulates (i.e., ephemeroviruses, tibroviruses, vesiculoviruses). Vector-borne rhabdoviruses were present in 12 of the 17 groups, dominating the dimarhabdovirus supergroup, but were largely absent from clades associated with bats (*Lyssavirus*), flies (*Sigmavirus*) and fish (*Perhabdovirus*, *Sprivivirus*) (Fig. 2). The exception to this trend was the *Tupavirus* clade, which comprised viruses that have not yet been associated with a vector species, and for which little is known about their ecology or distribution.

Each of the seven newly proposed rhabdovirus genera formed an independent, well-supported monophyletic group in the L protein phylogeny (BSP ≥ 85), and comprised viruses with similar genome organization (Fig. 1; Fig. 2). In several instances, viruses clustered closely with other members of a genus, yet we considered them to be unassigned species due to major differences in genomic architecture (see below). For example, the newly proposed genus *Curiovirus* comprises a monophyletic group of four viruses isolated from biting midges (*Culicoides* sp.), sandflies (*Lutzomyia* spp.) and mosquitoes (*Coqillettidia* and *Trichoprosopon* spp.) from the forests of South America and the Caribbean (S1 Table). The genomes of CURV, IRIRV, RBUV and ITAV all have one or more ORFs located between the M and G genes, and the G and L genes. In contrast, the closely related ARUV and INHV lack additional genes between the M and G and for this reason we have excluded them from the genus *Curiovirus* at this time. We also recognize the previous suggestion that CURV and ITAV should be assigned to a new genus for which the name *Bracorhabdovirus* (Brazilian Amazonian *Culicoides* rhabdoviruses) was proposed [27]. However, our analysis clearly indicates that this monophyletic group has a broader host range and geographic distribution than this regionally-derived name suggests.

Five of the novel viruses (comprising four putative new species) identified in this study were assigned to established genera. Two of these, KOOLV and YATV, clustered within the existing *Ephemerovirus* clade, (BSP ≥ 85) and possessed the characteristic genome organization of ephemeroviruses, including a non-structural glycoprotein gene (G_NS_) followed by a viroporin (α1) and several other small proteins (Fig. 1; Fig. 2). Similarly, two novel viruses isolated from biting midges (*Culicoides insignis*), SWBV and BAV, clustered within the genus *Tibrovirus* (BSP ≥ 85) and exhibited the conserved N-P-M-U_1_-U_2_-G-U3-L genome organisation (Fig. 1; Fig. 2; S1 Table). SWBV was assigned as a new species (*Sweetwater Branch virus*), but BAV is closely related to TIBV and may be regarded as the same species (*Tibrogargan virus*). Finally, a novel tupavirus (KLAV) identified from two species of vole (*Microtus* and *Clethrionomys* spp.), clustered with the TUPV and DURV clade in the L protein phylogeny (Fig. 2; S1 Table).

A more detailed rationale for the assignment of viruses to existing and proposed new genera is provided as supplementary text.

### Evolution of genome size and complexity

We identified a 48.5% variation in genome size from the smallest genome (FUKV, *Ledantevirus*; 10,863 nt) to the largest in our data set (KOOLV, *Ephemerovirus*; 16,133 nt). All genomes, including those for which extreme terminal sequences were unresolved, appeared to fall within this range. Variations in genome size were associated with: i) variation in the length of intergenic regions (IGRs) between transcriptional units; ii) variation in the length of 3’ and 5’ untranslated regions (UTRs) within individual transcriptional units; iii) the presence of additional transcriptional units containing long ORFs; and iv) the presence of overlapping or consecutive long ORFs within individual transcriptional units. An examination of genome size across the phylogeny revealed a general trend towards larger genomes in the lower third of the tree, which is comprised of the hapaviruses, curioviruses, tibroviruses and ephemeroviruses, as well as several unassigned viruses (S1 Fig.). Although this may indicate that an enhanced capacity for genome expansion is a property specific to this group, variation in genome size can also be observed between viruses in the majority of genera in the data set.

Several clade-specific patterns were evident when the lengths of the transcriptional units and IGRs were compared within and between rhabdovirus genera (Table 1). Ledantevirus genomes were smallest on average (1.75 × the length of the L) whereas ephemeroviruses genomes were the largest (2.37 × the length of the L, Table 1). Interestingly, although substantial variation in the length of gene junctions was observed in several genera (including ephemeroviruses and lyssaviruses), most variation in genome size occurred as the result of the presence of new, non-canonical ORFs in the regions between the structural protein genes (Table 1). Although new ORFs were observed in each IGR across the phylogeny (N-P, P-M, M-G and G-L) their location was primarily restricted to a single IGR within each genus. For example, while hapavirus genome expansion occurred primarily in the P-M junction, genome expansion in the ephemeroviruses occurred at the G-L junction and tibrovirus and curiovirus genomes contained additional ORFs primarily in the M-G junction (Table 1). This suggests that once a new ORF arises at a particular gene junction within a lineage, further expansion is more likely to continue at the same gene junction, rather than begin anew elsewhere in the genome.

**Table 1:** Averages (standard deviation) of the actual lengths of each genome region (nt), and the normalized lengths relative to the length of the L gene.

Genus	Length	N-P junction	P-M junction	M-G junction	G-L junction	Non-coding	New ORFs	Genome
***Ephemerovirus***	Normalized*	1.31 (0.88)	1.11 (0.66)	0.96 (0.48)	57.18 (8.95)	14.05 (7.59)	0.49 (0.03)	2.37 (0.10)
	actual	84 (55)	71 (42)	61 (31)	3655 (557)	897 (480)	3137 (209)	15147 (604)
***Curiovirus***	normalized	0.44 (0.07)	0.48 (0.09)	11.18 (2.82)	11.79 (4.71)	5.56 (3.59)	0.20 (0.08)	2.05 (0.06)
	actual	28 (5)	31 (6)	714 (181)	753 (304)	355 (229)	1271 (539)	13092 (435)
***Tibrovirus***	normalized	0.45 (0.06)	2.69 (4.97)	19.21 (1.03)	6.66 (0.79)	9.78 (0.82)	0.21 (0.01)	2.08 (0.01)
	actual	28 (4)	160 (290)	1202 (27)	419 (60)	623 (52)	1331 (39)	13233 (74)
***Hapavirus***	normalized	0.80 (0.72)	21.61 (8.31)	2.29 (3.67)	13.77 (13.51)	7.13 (3.60)	0.34 (0.13)	2.13 (0.16)
	actual	51 (46)	1370 (526)	145 (234)	875 (857)	453 (229)	2140 (849)	13544 (985)
***Vesiculovirus***	normalized	1.23 (0.70)	1.16 (0.65)	1.55 (1.15)	1.82 (1.49)	8.30 (3.40)	0.01 (0.01)	1.77 (0.04)
	actual	78 (44)	73 (40)	98 (71)	115 (94)	522 (211)	29 (89)	11158 (240)
***Ledantevirus***	normalized	0.48 (0.15)	0.36 (0.07)	0.47 (0.17)	1.42 (1.78)	4.51 (1.50)	0.01 (0.01)	1.75 (0.04)
	actual	30 (10)	23 (4)	30 (11)	90 (113)	284 (50)	52 (95)	11125 (257)
***Sripuvirus***	normalized	3.93 (3.39)	0.56 (0.19)	4.25 (0.03)	0.62 (0.10)	6.58 (3.28)	0.06 (0.02)	1.79 (0.03)
	actual	248 (214)	35 (12)	268 (3)	39 (6)	415 (206)	346 (99)	11279 (149)
***Tupavirus***	normalized	0.66 (0.06)	0.45 (0.01)	5.53 (0.35)	2.63 (3.01)	5.58 (0.56)	0.06 (0.02)	1.81 (0)
	actual	42 (4)	29 (1)	350 (22)	167 (190)	353 (35)	363 (115)	11459 (27)
***Bahiavirus***	normalized	2.68 (0.33)	1.53 (0.24)	2.01 (1.38)	3.69 (0.26)	11.86 (2.14)	0.03 (0)	1.93 (0.02)
	actual	175 (21)	100 (16)	131 (90)	241 (17)	774 (140)	221 (24)	12615 (31)
***Sawgravirus***	normalized	1.30 (0.33)	0.89 (0.08)	1.29 (0.27)	0.83 (0.73)	6.75 (1.10)	0 (0)	1.76 (0.02)
	actual	83 (21)	57 (5)	81 (18)	54 (47)	432 (71)	0 (0)	11267 (175)
***Lyssavirus***	normalized	1.41 (0.18)	1.33 (0.23)	3.25 (0.09)	8.95 (1.41)	17.61 (1.75)	0.01 (0.01)	1.88 (0.02)
	actual	90 (12)	85 (15)	208 (6)	571 (90)	1125 (112)	30 (77)	11983 (94)

*For clarity, the normalized length of each intergenic region is shown multiplied by 100.

**Fig 3:**
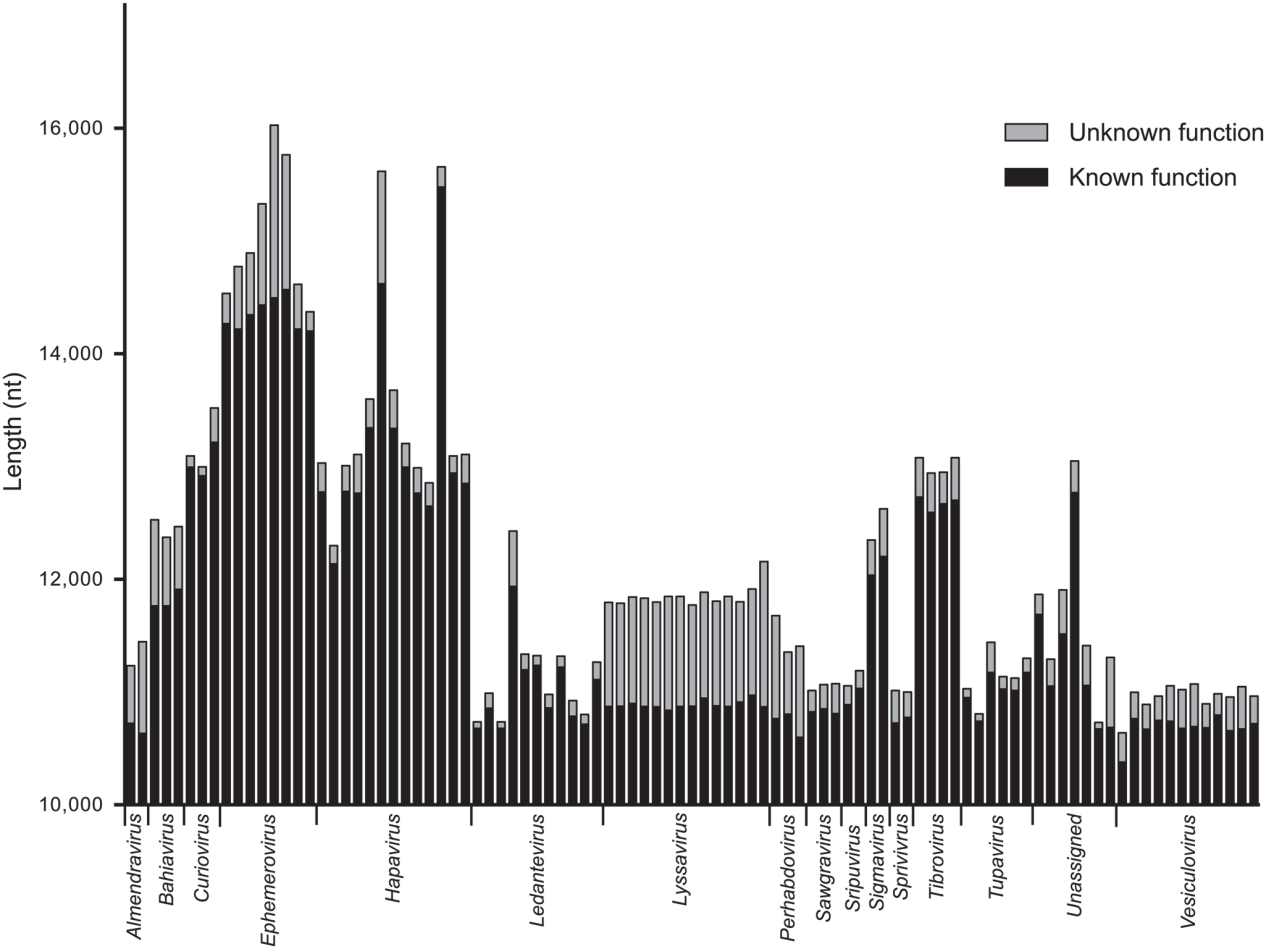
The relative length of sequences of known or predicted function and unknown function. Sequences of known or predicted function include ORFs and transcriptional regulatory sequences. Sequences of unknown function include 5’- and 3’-UTRs within transcriptional units and IGRs between transcriptional units. Genomic leader sequences (up to the N gene TI sequence) and trailer sequences (beyond the L gene TTP sequence) were excluded from the analysis as the extreme terminal sequences of some viruses were not determined. The sequence lengths are provided for each virus in the data set but identified only by their genus assignment.

Whilst the genome architecture in some viruses was highly compact, others featured long stretches of sequence with non-ascribed function that occurred primarily as 5’UTRs and 3’UTRs within transcriptional units (Fig. 3). The proportion of untranslated sequences within or between transcriptional units ranged from 0.5% (FUKV; 58 nt) to 10.6% (WCBV; 1290 nt) and did not correlate with genome size. Furthermore, although all lyssaviruses (such as WCBV) featured a high proportion of untranslated sequences (primarily evident as a very long 3’UTR in the G gene), there was no consistent association between the proportion of untranslated sequences and genus assignment (Fig. 3). For example, in the genus *Hapavirus*, the proportion of untranslated sequences in the two largest genomes varied from 1.1% (NGAV) to 6.4% (LJV). Similarly, in the genus *Ephemerovirus* the proportion of untranslated sequences varied from 1.2% in the smallest genome (YATV) to 9.6% in the largest genome (KOOLV). The presence of long stretches of untranslated sequence, which occurred primarily within transcriptional units, suggests these regions may be functional. However, it is unclear at this time why they are present in some rhabdoviruses and not in others.

**Gene duplication**. Previous studies have provided evidence of gene duplication in the *Rhabdoviridae*, involving the G and G_NS_ genes [18,21] and the β and γ genes [22] in the ephemeroviruses, and the U1, U2 and U3 genes in the hapaviruses FLAV and WONV [15,19,20]. To identify further examples of gene duplication, we conducted a BLAST analysis of all proteins in our database (E-value <1e-3) and used ClustalX alignments to confirm sequence similarity. By this analysis, ORFs located between the P and M genes of most hapaviruses encode proteins which share detectable sequence similarity. This family of homologous P-M intergenic region proteins (PMIPs) includes the U1, U2 and U3 proteins of LJV, WONV, PCV, ORV, LJAV, MANV, MQOV, FLAV, HPV, KAMV and MOSV (S2 Fig. and S3 Fig.), as well as the U1x proteins of MANV and GLOV which are encoded in ORFs overlapping their respective U1 ORFs (S4 Fig.). Although pairwise alignments provide clear evidence for homology, the hapavirus PMIPs share generally low levels of sequence identity and no universally conserved motifs, indicating considerable structural and functional divergence from their ancestral homolog. Proteins encoded in the P-M region in other hapaviruses (i.e., JOIV U1, NGAV U1, U1x and NGAV U2) failed to display significant similarity with the PMIPs or evidence of gene duplication but this may be due to further structural divergence. Additional evidence of gene duplication included the U2 and U3 proteins of JOIV (encoded in ORFs located between the G and L genes), and the N-terminal regions of the P proteins and the upstream U1 accessory proteins of the sripuviruses CHOV and SMV, each of which share significant sequence similarity (S5 Fig.). These data suggest that the U1 protein of the sripuviruses originated from a duplication of the P gene, with the downstream copy of the gene retaining the parental function. Similarly, in the curioviruses there is extensive amino acid sequence similarity between the U3 proteins of CURV and IRIRV and the N-terminal region of the G proteins, suggesting evolution of U3 through partial duplication of the G gene, which lies immediately downstream.

### Accessory genes and gene families

Putative accessory genes were found to be abundant and varied greatly in number and location in each genome (Fig. 1). A complete list of ORFs >180 nt is annotated in S2 Table. In most cases, homology searches detected no significant amino acid sequence identity with entries in GenBank. However, various rhabdovirus accessory gene families were identified based on amino acid sequence identity in our custom BLAST searches, or common structural characteristics.

**Fig 4:**
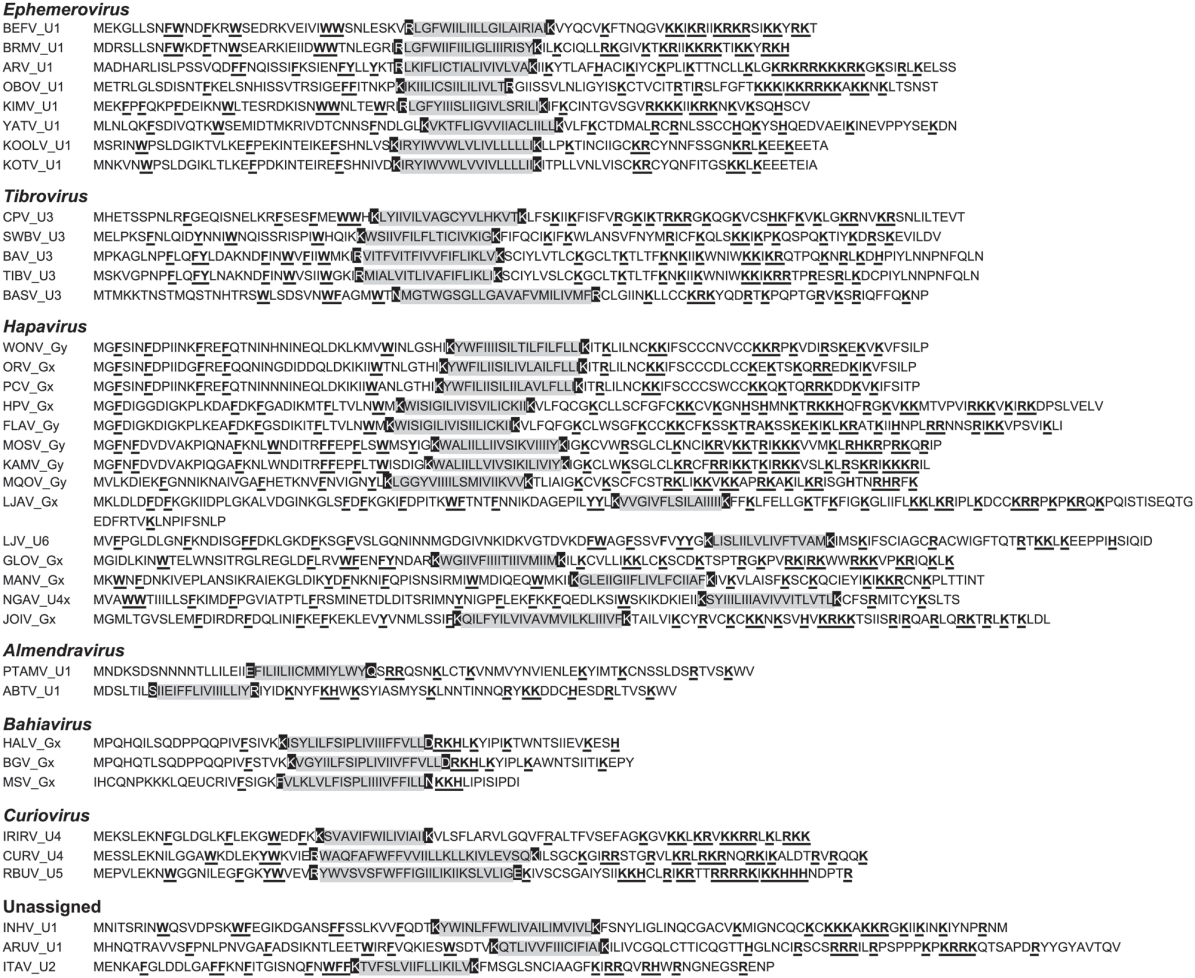
Illustration of the similar structural characteristics of viroporin-like proteins. The sequences illustrate predicted hydrophobic transmembrane domains (grey shaded) which are usually bounded by anchoring charged residues (black shaded), an N-terminal domain containing several large aromatic residues (F, Y, W), and a C-terminal domain containing a large number of basic residues (R, K, H) (bold and underlined). The proteins are assigned numbers according to our defined annotation rules and are grouped according to existing genera and new genera proposed in this paper.

**Viroporins**. Viroporins are small hydrophobic proteins that oligomerize in host cell membranes to form hydrophilic pores, disrupting various cellular processes and promoting virus replication [28]. ORFs encoding viroporin-like proteins were found in more than one-third of the rhabdoviruses in the data set, either as overlapping or consecutive ORFs within the G gene, or in additional transcriptional units following the G (or G_NS_) gene (Fig. 1). ORFs encoding putative viroporins were evident in the genomes of all ephemeroviruses, tibroviruses, hapaviruses, bahiaviruses, almendraviruses and curioviruses, as well as the unassigned species ARUV and INHV (Fig. 4). Several of these proteins have been identified previously [19,22,29–35]. Like the BEFV α1 protein for which viroporin activity has been confirmed experimentally, these proteins have the structure characteristics of class IA viroporins, including a central transmembrane and a highly basic C-terminal domain. However, although located in similar positions in the genomes, they are generally too divergent in sequence to establish orthology [22,36].

**Other small transmembrane proteins**. Small proteins with a predicted central transmembrane domain but lacking other characteristics of class 1A viroporins were identified in several other rhabdoviruses (S6 Fig.; S2 Table). Transmembrane proteins with an N-terminal ectodomain are encoded in the Gx ORF of sripuviruses and the U3 ORF of one curiovirus (RBUV). However, in other curioviruses (CURV and IRIRV), transmembrane proteins are encoded in the U2 ORF and are predicted to have the reverse membrane topology to the RBUV U3 protein. Sequence alignments further suggest these proteins are not orthologous. There is also a small double-membrane spanning protein with a predicted short ectodomain loop encoded in an alternative ORF in the FUKV M gene that is not present in other ledanteviruses.

**Other small hydrophobic (SH) proteins**. Small highly hydrophobic proteins (6.8–10.8 kD) lacking predicted transmembrane domains are encoded in all tupaviruses (as independent transcriptional units following the M gene) and sripuviruses (as overlapping ORFs within the M gene) (S7 Fig.; S2 Table). All have similar hydropathy profiles with a highly hydrophilic N-terminal domain extending to the centre of the sequence, but sequence identity indicative of orthology is restricted to closely-related viruses. Several of these SH proteins have been identified previously but their function remains unknown [37–40].

**Large class I transmembrane glycoproteins**. All ephemeroviruses encode a class I transmembrane glycoprotein (G_NS_) in the ORF following the G gene [18,21,30,31]. NGAV (assigned to the proposed new genus *Hapavirus*) also encodes a G_NS_ protein with similar structural characteristics [35]. However, as we found no evidence to support recombination between NGAV and any ephemerovirus, the NGAV G_NS_ gene is likely to have arisen by an independent duplication event of the upstream G gene with which it shares amino acid sequence identity. ORF U1 immediately following the MCOV G gene (genus *Hapavirus*) also encodes a large class I transmembrane glycoprotein but lacks the set of conserved cysteine residues that are characteristic of G and G_NS_ proteins, and our homology searches failed to identify similarity with any known protein (S8 Fig.).

**Other genus-specific accessory gene families**. Orthologous sets of accessory genes occur in genus-specific patterns in each of the structural protein gene junctions (Fig. 1; S2 Table). In addition to the hapavirus PMIP genes, these include genes in the N-P junction of sripuviruses CHOV and SMV (U1 proteins), the M-G junction of curioviruses (U1 and U1x proteins) and tibroviruses (U1 and U2 proteins), and the G-L junction of curioviruses (U3x proteins) and ephemeroviruses (α2, β, γ and δ proteins) (S9 Fig. to S11 Fig.). Some of these orthologous gene sets have been described previously [15]. Most encode proteins without remarkable structural characteristics and of unknown function (S2 Table).

### Non-canonical gene expression

Several general architectural patterns in the arrangement of ORFs were evident, implicating several mechanisms of non-canonical gene expression. Non-cannonical expression mechanisms are used commonly in other families of RNA viruses to increase genome complexity without significantly increasing genome size [41]. The patterns we observed in this data set were associated with consecutive, overlapping of alternative ORFs within individual transcriptional units.

**Fig 5:**
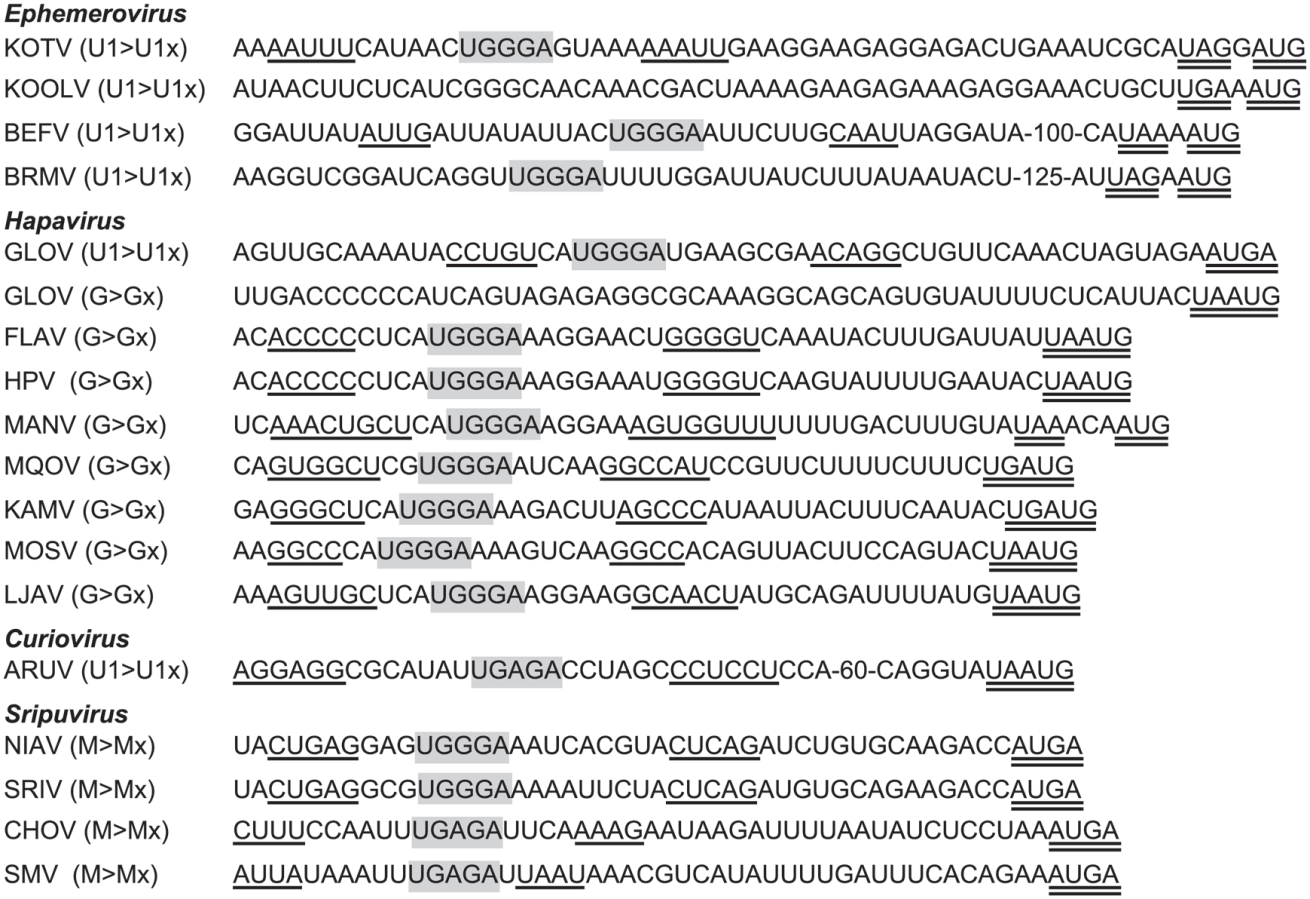
TURBS-like sequence motifs in the genomes of sripuviruses, curioviruses, hapaviruses and ephemeroviruses. The motif features the sequence UGGGA (highlighted) flanked short stretches of anti-complementary sequence (single underlined) upstream of overlapping or adjacent initiation and termination codons (double underlined). Variations in the TURBS sequence (UGAGA) occur in CHOV, SMV and ARUV. The ORF junctions (M-Mx; U1-U1x; G-Gx; α1-α2) are indicated for each virus. No TURBS-like sequence was detected upstream of the adjacent initiation and termination codons at the GLOV G—U3 junction or the KOOLV α1-α2 junction.

**Consecutive ORFs and TURBS motifs**. Consecutive long ORFs with termination and initiation codons that are either overlapping (e.g., UAAUG) or separated by a short stretch of nucleotides were common in several groups of rhabdoviruses (Fig. 5). As previously observed for FLAV, this ‘stop-start’ arrangement is commonly preceded by a ‘termination upstream ribosome-binding site’ (TURBS), which contains a short sequence motif that is complementary to the loop region of helix 26 of 18S ribosomal RNA [19,41]. The TURBS may also contain flanking anti-complementary sequence motifs that are predicted to form a stem-loop structure. This arrangement was found in the M transcriptional unit in the sripuviruses, the G transcriptional unit of several hapaviruses (FLAV, HPV, MANV, MQOV, KAMV, MOSV and GLOV) and the transcriptional unit between the P and M genes of GLOV. The ‘stop-start’ arrangement also occurs in the transcriptional unit between the G and L genes of ARUV, allowing expression of the U2 ORF, but in this case the TURBS appears to be further upstream of the stop-start site. Finally, the α gene transcriptional unit in most ephemeroviruses contains consecutive ORFs encoding a viroporin (α1) and a second protein of unknown function (α2). In KOTV, a TUBRS is evident upstream of the stop-start site but in other ephemeroviruses the TURBS appears to be more cryptic.

**Overlapping ORFs and ribosomal-frame shift (RFS) sites**. Overlapping ORFs are common in rhabdovirus genomes and represent a second common architectural arrangement requiring non-canonical gene expression. Overlapping ORFs occur within the N transcriptional unit (WONV, ORV, PCV, MCOV, MANV), the G transcriptional unit (WONV, ORV, PCV, BGV, HARV) or within additional transcriptional units between the P and M genes (MANV, NGAV) or the M and G genes (CURV, IRIRV, RBUV). Expression of the second ORFs in these arrangements would require either internal initiation in an alternative reading frame or another mechanism such as RNA editing or a ribosomal frame-shift (RFS) to extend the first ORF. Use of alternative initiation codons has been reported in the M and P genes of VSV and the P gene of RABV, and RNA editing has been described in the P gene of paramyxoviruses [23,42–45]. Although not described previously in mononegaviruses, potential RFS sites were identified in some of these rhabdovirus gene overlap regions, featuring the ‘slippery’ sequence motifs UARUUUUUUCA (BGV, HARV, MSV) or CCNUUUUUUGA (WONV, ORV, PCV) followed by a predicted stem-loop structure (S12 Fig.). These sequence motifs and associated stem-loop structures most closely resemble the-1 RFS that allows expression of *gag-pol* in HIV-1 and other lentiviruses [41,46].

**Fig 6:**
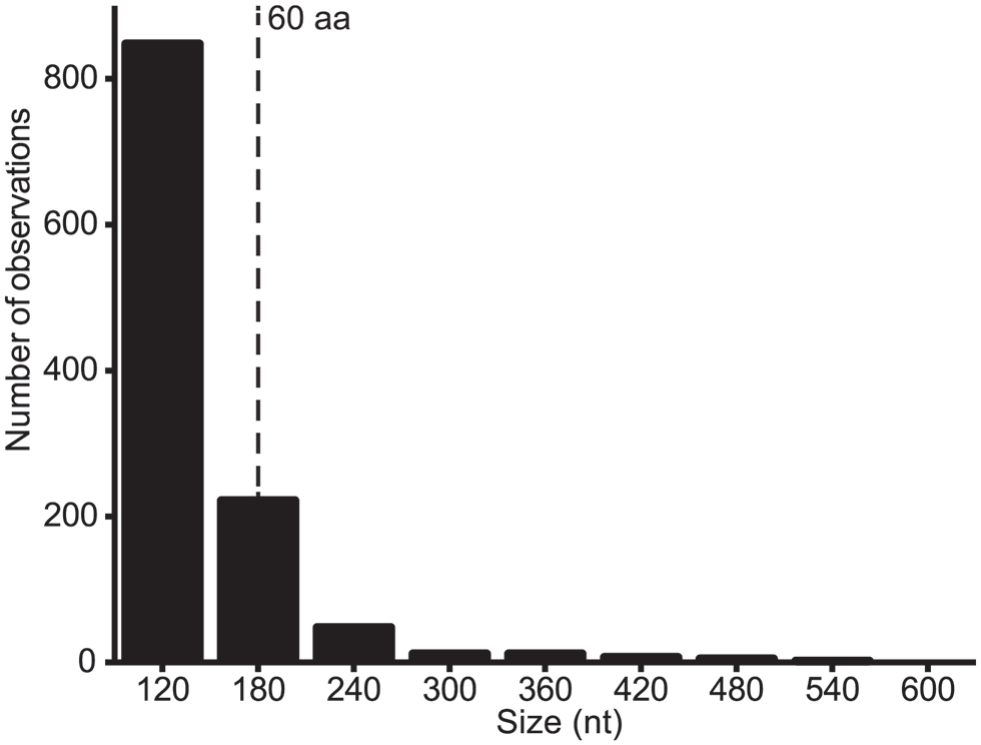
Number of alternative ORFs of various size ranges (nt) across the genome data set. ORFs ≥ 90 nt were identified in each genome and the assembled set was grouped into size ranges (i.e., 90–149, 150–209, 210–269, etc). The total number of observations of ORFs in each size range is shown. All ORFs ≥180 nt (60 aa) are listed in S2 Table.

**Alternative ORFs and leaky ribosomal scanning**. The third architectural arrangement involves the use of alternative ORFs within a longer ORF. This arrangement was described previously in VSIV, in which two small basic proteins of 55 and 65 amino acids (C and C’) are expressed from an alternative ORF within the P gene [23,24]. On this basis, we scanned the rhabdovirus genome data set for alternative ORFs of various size ranges and observed that the frequency varied from ~2.3/genome for ORFs in the range of 90–150 nt (30–50 amino acids) to ~8.6/genome for range 150–210 nt (30–70 amino acids) (Fig. 6). Alternative ORFs ≥60 amino acids occurred in each of the structural protein genes (N, P, M, G and L) and in the additional transcriptional units between the P and M genes. They were most common in the P and least common in the M genes. As observed in other viruses, expression of these alternative ORFs could occur by leaky ribosomal scanning, allowing initiation of transcription by a proportion of ribosomes on the alternative start codon [41]. Although, it is not known which (if any) of these alternative ORFs are expressed, several factors are likely to be important in determining the probability and level of expression: i) the Kozak contexts of the first and alternative initiation codons; ii) the length of the alternative ORF (longer ORFs are less likely to occur by chance); iii) the location of the alternative ORF (distally located ORFs are less likely to be expressed in long transcripts); and iv) the expression level of the transcript (L gene transcripts are likely to be the least abundant). For example, short ORFs with initiation codons in poor Kozak context at the distal end of the L gene are not likely to be expressed at significant levels, if at all. However, in some cases, closely related viruses were found to contain alternative ORFs at the same genome location, with initiation codons in good context and encoding predicted polypeptides with high levels of sequence identity (S2 Table). Such arrangements occurred in the N genes of HPV and FLAV, the P genes of MANV and MQOV, the U2 and M genes of KAMV and MOSV, and near the start of the G genes of the sripuviruses (NIAV, SRIV, CHOV and SMV); these proteins are considered very likely to be both expressed and functional.

## Discussion

We have conducted a detailed analysis of the structural organisation and genome evolution of a family of negative-sense RNA viruses—the *Rhabdoviridae*. Previous studies have surveyed known rhabdoviruses for biological and genomic diversity, revealed phylogenetic relationships, and considered factors that may have determined their rates of evolution [14,15,47,48]. In this study, we greatly expanded the repertoire of rhabdovirus genome sequences, which demonstrate extensive variation in genome size and complexity, allowing the assignment of seven proposed new genera. We also identified patterns of accessory gene evolution and expression, and showed that changes in rhabdovirus genome length and composition have occurred throughout the evolutionary history of the family, primarily through the generation and loss of new transcriptional units. This observation is especially striking given the obvious constraints on viral genome size [7].

The most remarkable aspect of this analysis is the number and variety of additional ORFs identified in rhabdovirus genomes, which provides a very different perspective of the family and its evolution than had been obtained from studies of the traditional prototype members (VSIV and RABV). As many of these ORFs occur as additional transcriptional units complete with conserved transcriptional control sequences, there is a high likelihood that they would be expressed in infected cells. Expression of ORFs located in additional transcriptional units has been demonstrated previously for several ephemeroviruses and for the hapavirus WONV [18,21,30,31,36,49]. Others occur as either alternative or overlapping ORFs. Further studies are required to determine which of these ORFs may be expressed, but we suggest that expression is likely when both the encoded amino acid sequence and the translational context are conserved in related species.

Notably, very few of the additional ORFs detected in this analysis encode proteins with identifiable sequence similarity to other known proteins. Sequence similarity, when detected, occurred only between closely related viruses assigned to a genus and, although some accessory protein families were identified, these were more commonly related by shared structural characteristics, such as charged or transmembrane domains, than by sequence. This has been observed previously for so-called orphan (‘ORFan’) proteins in other viruses and bacteria. It has been suggested that the uniqueness of orphan proteins, or their restriction to a single species or genus, is the result of creation *de novo*, rather than by recombination or lateral gene transfer, and that they play an ‘accessory’ role in viral pathogenicity or transmission instead of having functions in virion structure or replication [50–52]. It has also been observed that many orphan proteins are predicted to be highly disordered in structure or, when ordered, structural resolution has revealed unique folds [50]. As such, future determination of the biological activities of the plethora of novel proteins identified here will require functional studies that may well provide important insights into aspects of infection and immunity as well as fundamental cellular processes and pathways.

Substantial variation in genome size and complexity was also observed in many rhabdovirus genera, suggesting that the length of the genome is not heavily constrained in all members of the family. Indeed, the presence of new ORFs and/or very long stretches of non-coding sequence within or between transcriptional units was noted frequently. Previous observations have demonstrated that foreign genes of up to ~6 kb can be inserted into the VSIV genome without significant disruption to viral replication *in vitro* [53,54]. Expanded VSIV genomes were morphologically similar but proportionally longer than wild-type viruses, suggesting that the unique morphology of the rhabdovirus particle may more readily accommodate genome expansion than other virion structures. A significant body of evidence suggests that genome size in RNA viruses is likely to be constrained by low replication fidelity [7,8], and a relationship between genome size and error rate has been observed in a diverse array of organisms [55]. However, if the genome sizes of rhabdoviruses are constrained by selective pressures other than (or in addition to) those imposed by the background mutation rate, genome expansion may not require a concomitant reduction in polymerase error rates. As the mutation rate of rhabdoviruses has only been determined experimentally for VSIV thus far (~6 × 10^–6^ subs/nucleotide/replication), it is impossible to assess whether the increases in genome size observed here have been associated with concomitant reductions in mutation rate [48].

It is also striking that while some rhabdovirus genomes appear to have undergone major changes in length and complexity, others contain only the 3’ and 5’ promoter regions and five canonical transcriptional units with minimal 5’ and 3’UTRs. This suggests that the acquisition and loss of new genes and intergenic regions may be a regular feature of rhabdovirus evolution. Previous studies of RNA viruses have concluded that constraints on genome size imposed by polymerase error have led to various strategies to minimize genome size while increasing functional complexity, such as gene overlaps and protein multi-functionality [9,56]. Given these size constraints, it is unclear why long non-coding regions would arise both within and between transcriptional units and be maintained throughout the evolution of some rhabdovirus genera. It has been known for many years that a long 3’-UTR of unknown function (ψ region) in the G gene of RABV is unnecessary for efficient replication in cell culture or in mice, but may play a role in neuroinvasion [57–59]. Indeed, the retention of similar ψ regions in all lyssaviruses and the existence of long UTRs and IGRs in other rhabdoviruses suggests that they must provide some fitness advantage *in vivo*, such as stabilising RNA secondary structure, serving as a source of, or targets for, micro RNAs, or attenuating transcription of downstream genes to achieve the most effective balance of gene expression. Indeed, an analysis of patterns and rates of sequence evolution in the *Rhabdoviridae* and other families in the *Mononegavirales* revealed that, although non-coding regions are less conserved than those that encode proteins, their evolutionary rates are associated with relative genomic position, suggesting that they impact on gene expression [60].

Additional ORFs and non-coding sequences occurred at all junctions of the canonical structural protein genes (i.e., N-P, P-M, M-G, and G-L), although there was variation in both the frequency of insertion and the extent of expansion. Notably, insertions at the N-P junction are rare, with a single additional ORF present in the closely related sripuviruses CHOV and SMV, and short overlapping ORFs present within the N gene transcriptional unit in some hapaviruses. It has been reported previously in a study of VSIV recombinants that only the N-P gene junction was refractory to the stable expression of an inserted transcriptional unit, and resulted in a virus with significantly reduced replication efficiency [61]. In contrast, transcriptional units inserted at other gene junctions were stably expressed, maintained through repeated passages and had no effect on replication efficiency. As the insertion of additional transcriptional units attenuates expression levels of all downstream genes, this may be associated with the importance of maintaining precise control of N and P protein ratios in infected cells to ensure efficient switching between the transcription and replication modes of the ribonucleoprotein complex [62,63].

**Fig 7:**
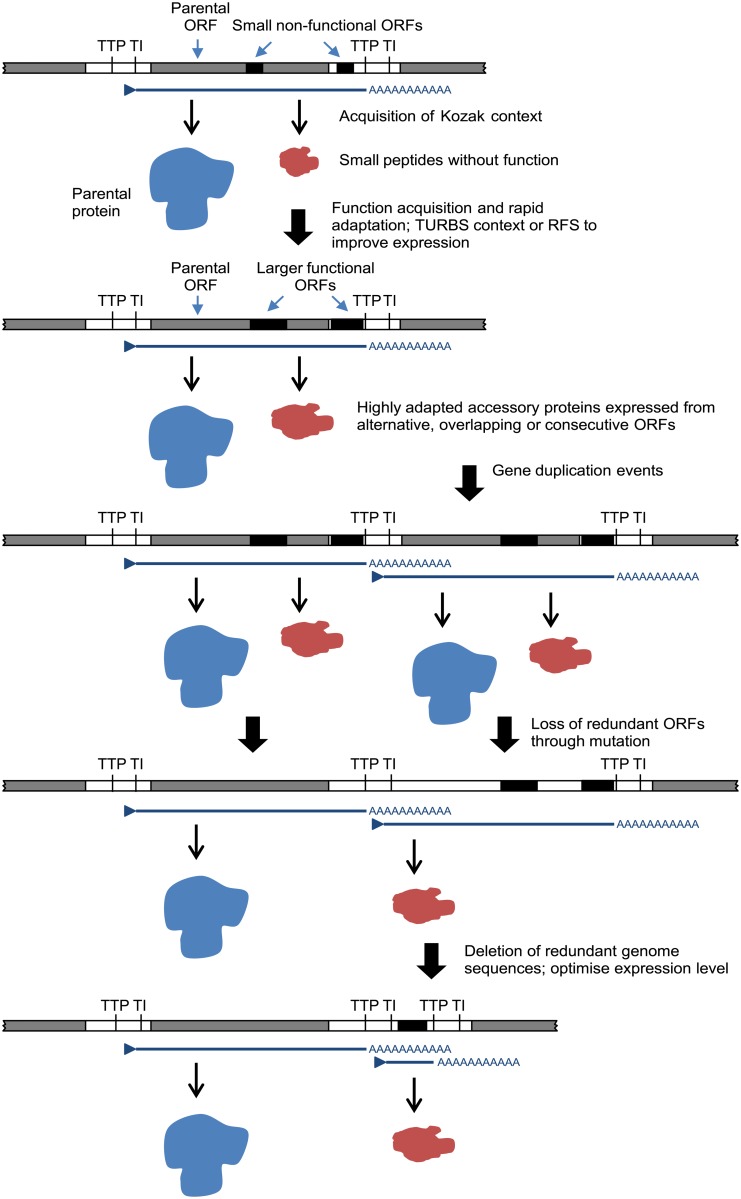
A model for the evolution of rhabdovirus accessory genes. The model accounts for accessory genes that emerge initially from small ORFs arising randomly through mutation in alternative reading frames within existing ORF or in 5’ or 3’UTRs within transcriptional units.

The relationships, locations and contexts of additional ORFs in various viruses lead us to propose a general model for rhabdovirus genome plasticity, which can account for both gains and losses in genome size and complexity (Fig. 7). In each of these viruses, small ORFs of various lengths occur within most transcriptional units; and although only those ≥180 nt have been catalogued here, there are numerous other smaller ORFs throughout most genomes. It is reasonable to assume that, although the polypeptides encoded in many of these ORFs may not be expressed at all during infection, some may be expressed through leaky ribosomal scanning. These are likely to represent a rich genetic resource for the evolution of new functional genes in RNA viruses [4], triggering the rapid evolution of highly specialised functions. Contemporarily, the evolution of a suitable Kozak context, TURBS motifs and ribosomal frame-shift sites would allow optimal expression within the parental transcriptional unit. Ultimately, these new ORFs may become uncoupled from the parental gene through gene (sequence) duplication [18]. As observed previously, this process would allow unconstrained evolution of the new ORF and loss of the redundant copy of the parental ORF [4,64]. Alternatively, new genes may also evolve independently of existing ORFs. In some rhabdoviruses in our data set, very long non-coding regions (up to 749 nt) were present either within or between transcriptional units that could serve as a resource to spawn genes *de novo* in the absence of the evolutionary constraints imposed on alternative or overlapping ORFs. This is most likely to occur when ORFs are present in transcribed non-coding regions (UTRs) such as the ψ region of WCBV in which, uniquely amongst lyssaviruses, an ORF of 180 nt has been identified [65]. The creation of new genes *de novo* in non-transcribed IGRs, such as those present in the G-L gene junctions of LJV, KOTV and KOOLV, almost certainly would require prior or simultaneous evolution of new or modified transcriptional control sequences to allow their expression.

We recognise that other mechanisms of genome expansion are also possible. In Central American isolates of VSIV, for example, imprecise reiterative insertions of up to 300 nt in the 5’-UTR of the G-gene (variations of 3’-UUUUUAA-5’) have been attributed to non-templated extension by polymerase stutter at the TTP sequence [66,67]. Although homologous recombination appears to be very rare in mononegaviruses [68], and we found no evidence of lateral gene transfer, we cannot exclude their involvement in rhabdovirus genome expansion. It is also evident that although there is an overall trend toward an expansion of genome size and complexity in the rhabdoviruses, gene loss is also likely to have occurred periodically throughout the evolution of the family. For example, the ephemerovirus γ proteins appear to have been lost in ARV and OBOV, and the hapavirus PMIPs are entirely absent only from MCOV (Fig. 1). Although our data suggests that gene gain is a more frequent process than gene loss, we acknowledge that, if loss is very frequent, we might not be able to observe it given the available data. This may be resolved in the future with the acquisition of significantly more genomes sampled more closely in time. Indeed, as defective-interfering particles are known to occur commonly in rhabdoviruses, a mechanism for purging redundant sequences appears to be readily available [69–71]. Nevertheless, it is evident that a remarkable capacity for genomic plasticity through the gain and loss of accessory functions has been a central theme of rhabdovirus evolution.

Although our analysis was limited to the *Rhabdoviridae*, similar mechanisms of genome expansion appear to occur in other families of non-segmented (-) ssRNA viruses (*Mononegavirales*). For example, amongst the *Paramyxoviridae* genome length varies by 46.5% from human metapneumovirus (13,113 nt) to Beilong virus (19, 212 nt), and paramyxoviruses also contain novel accessory genes in transcriptional units inserted at various gene junctions [72]. The apparent propensity for genome expansion in mononegaviruses may be due to their discontinuous transcription strategy which generates multiple viral mRNAs. Sequence insertions within and between the individual transcriptional units of mononegaviruses are less likely to disrupt gene expression than in (+) ssRNA viruses in which the genome commonly encodes a single polyprotein which is processed post-translationally.

Finally, this study has also provided an important advance in rhabdovirus taxonomy, allowing the assignment of six new species to existing genera and the assignment of 37 species to seven proposed new genera as well as the identification of six new unassigned species. There are currently no formal criteria for genus demarcation in rhabdoviruses. A system of genetic classification (DEmARC) that allows demarcation of viral taxa based on pairwise evolutionary distances has been proposed and, for picornaviruses, was shown to be comparable to expert-based taxonomic classification [73,74]. However, the application of this approach to the *Rhabdoviridae* would likely require a larger set of sequenced genomes at lower taxonomic levels [75], and would be compromised by extensive rate variation among lineages (as this leads to biases in genetic distance measurements). In the taxonomy of higher organisms, to be descriptively useful, a genus should be monophyletic, reasonably compact, and ecologically, morphologically, or biogeographically distinct [76]. Our assignment of new genera in the *Rhabdoviridae* has been based primarily on the identification of well-supported monophyletic groups using unambiguously aligned regions of the L gene, together with a consideration of common features of genome organisation and known aspects of viral ecology. Genome organisation has proven here to be a useful taxonomic marker as similar arrangements of accessory genes and other conserved elements of genome architecture appear to be the result of significant evolutionary events that provide resolution between the family and species levels. For some of the new genera, host and/or vector associations have also been relatively informative but in many cases, only single isolates of a species are available and else little is known of their ecology. It is likely that the proposed assignments of viruses to genera and the placement of the proposed unassigned species will evolve into a more complete taxonomic description as more viruses are discovered and as ecological data accumulates.

## Materials and Methods

### Viruses

Details of the viruses included in this study, including taxonomic status, sources and dates of isolation, and GenBank accession numbers of genome sequences are given in S1 Table. All but three viruses sequenced in this study were obtained from the World Reference Center for Emerging Viruses and Arboviruses (WRCEVA), located at the University of Texas Medical Branch, Galveston. Of the remaining viruses, FUKV and KOOLV were obtained from the collection held at the CSIRO Australian Animal Health Laboratory, Geelong, and JOIV was obtained from the QIMR collection held at the Queensland University of Technology, Brisbane, and kindly provided by Dr John Aaskov.

### Preparation of viral RNA and next generation sequencing

Viruses sequenced in this study were prepared as described previously [37]. With the exception of HPV, ITAV, CURV, GLOV, INHV, NMV, MEBV, YATV, LDV, GARV, CNTV, IRIRV, RBUV, BARV, LJAV, KEUV, MCOV, SMV, CHOV, PCV and BAV, which were sequenced directly from infected suckling mouse brain, viruses were sequenced from viral preparations grown in BHK-BSR, C6/36 or Vero cells monolayers. Sequencing was performed using either the Illumina HiSeq or MiSeq platforms. Viral RNA was fragmented by incubation at 94°C for 8 min in 19.5 l of fragmentation buffer (Illumina 15016648). A sequencing library was prepared from the sample RNA using an Illumina TruSeq RNA v2 kit following the manufacturer’s protocol. Samples were sequenced using the 2 × 50 paired-end protocol. Reads in fastq format were quality-filtered and any adapter sequences were removed using Trimmomatic software [77]. The *de novo* assembly program ABySS [78] was used to assemble the reads into contigs using several different sets of reads and k values from 20 to 40. The longest contigs were selected and reads were mapped back to the contigs using Bowtie 2 [79] and visualized with the Integrated Genomics Viewer [80] to verify that the assembled contigs were correct. Total reads ranged from 0.5 to 12 million and the percentage of reads mapping to the virus genome in each sample ranged from 0.2% to 33%. Details are available upon request.

### Sequence analysis

Assembly of full genome sequences was performed as previously described [37] and predicted ORFs >30 amino acids in length were identified across each genome using Geneious 7.0.6 (Biomatters Ltd). For each non-canonical ORF >60 amino acids in length, we sought to identify putative homologues by first comparing the protein sequence to the complete non-redundant protein sequence database available on GenBank using the BLASTp and PSI-BLAST search algorithms, as well as to the UniProt20 database using the hidden Markov model alignment-based algorithm HHblits[81]. For these searches, we investigated all matches with an E-value <1. We then created a custom protein database containing all ORFs >60 amino acids in length from our data set (648 proteins) and performed a custom BLAST search to identify homologues within this data set. Here, an E-value of <1e-3 was considered a significant match. Amino acid sequence alignments containing all putative matches to each ORF were then created using Clustal X and evidence of structural and sequence similarity was investigated by visual inspection. Structural predictions for proteins were conducted using Compute pI/MW, SignalP, TMHMM, TmPred, NetNES and NetNGlyc available through the ExPASy Bioinformatics Resource Portal (http://www.expasy.org/).

To quantify the location and extent of variation in genome size in our data set, we compared the average length of each genomic region within and between rhabdovirus genera. For all viruses, we normalized the length of each gene region (from the TI to TTP sequences, inclusively) and intergenic region by dividing by the length of the corresponding L gene, which varied least across the data set (coefficients of variation: N = 0.06, P = 0.12, M = 0.09, G = 0.13, L = 0.01). As there was substantial variability in the proportion of the 5’ and 3’ UTRs that were included in the sequence data set, we considered each genome to begin at the first TI sequence and end at the final TTP sequence for this analysis.

### Phylogenetic analysis

To infer evolutionary relationships among animal rhabdoviruses, we compiled sequences of the L (RNA-dependent RNA polymerase) protein, as this was the most highly conserved protein across the data set. We initially attempted to root the tree using a standard outgroup method. Members of the rhabdovirus genera that infect plants (i.e., *Cytorhabdovirus* and *Nucleorhabdovirus*) were excluded as their sequences were highly divergent. We therefore utilized four members of the genus *Novirhabdovirus (Infectious haematopoietic necrosis virus* ADB93801; *Viral hemorrhagic septicaemia* virus BAH57327; *Hirame rhabdovirus* ACO87999; and *Snakehead rhabdovirus* NP050585) as outgroups. Unfortunately, these novirhabdovirus sequences were also far too divergent (>>1 amino acid change per site under multiple amino acid substitution models; results available on request) to establish a reliable rooting for our data set, as three different basal groups were identified using different models of amino acid substitution, although overall tree topologies were similar among substitution models (results available on request). In addition, the use of the novirhabdoviruses as outgroups resulted in excessive numbers of residues being removed following Gblocks pruning (see below). Based on the observation that most known rhabdoviruses are either insect viruses or replicate in insect vectors, it has been reasonably argued that plant and animal rhabdoviruses may have origins in insects [82]. We therefore selected the rooting scheme that best fit this theory. To this end, we choose one of the two basal clades from the novirhabdovirus-rooted tree, comprising viruses isolated from mosquitoes (i.e., the almendraviruses), as the most divergent group. We then repeated the phylogenetic analysis (procedure described below) excluding the novirhabdoviruses and rooting it on the almendraviruses. Importantly, the choice of outgroup did not influence relationships either between or within the major clades demonstrating strong bootstrap support (BSP ≥ 85).

The alignment used for the final tree inference (i.e., excluding the novirhabdoviruses) was comprised of amino acid sequences aligned using the MUSCLE program [83], with ambiguously aligned regions removed using the Gblocks program with default parameters [84]. This resulted in a final sequence alignment of 100 taxa, 1007 amino acid residues in length. The phylogenetic relationships among these sequences were determined using the maximum likelihood (ML) method available in PhyML 3.0 [85] employing the WAG+Γ model of amino acid substitution and subtree pruning and regrafting (SPR) branch-swapping. The phylogenetic robustness of each node was determined using 1,000 bootstrap replicates and nearest-neighbour branch-swapping.

## Supporting Information

S1 Fig ML phylogenetic tree of 100 rhabdovirus L protein sequences with associated virus genome lengths. (PDF)

S2 Fig (A-K). Amino acid sequence alignments of various hapavirus PMIPs illustrating homologies that provide evidence for gene duplication. (PDF)

S3 Fig (A-Z). Amino acid sequence alignments of various hapavirus PMIPs that illustrate homologies between proteins from different viruses. (PDF)

S4 Fig (A-D). Amino acid sequence alignments of GLOV and MANV U1x proteins with other hapavirus PMIPs. (PDF)

S5 Fig (A-C). Amino acid sequence alignments of the U2 and U3 proteins of JOIV, the U1 and P proteins of CHOV and SMV, and the U3 and G proteins of CURV and IRIRV. (PDF)

S6 Fig Small transmembrane proteins encoded in the genomes of sripuviruses and the curioviruses. (PDF)

S7 Fig (A, B). Small hydrophobic proteins encoded in the genomes of tupaviruses, several unassigned viruses and sripuviruses. (PDF)

S8 Fig Sequence of the predicted class I transmembrane glycoprotein encoded in the MCOV genome. (PDF)

S9 Fig (A-D). Amino acid sequence alignments of small accessory proteins encoded in the genomes of ephemeroviruses. (PDF)

S10 Fig (A-D). Amino acid sequence alignments of the U1, U1x proteins, U3x and U4x proteins of the curioviruses, and of the RBUV U2 protein with the ITAV U1 protein. (PDF)

S11 Fig (A, B). Amino acid sequence alignments of the U1 and U2 proteins of tibroviruses. (PDF)

S12 Fig (A-E). Analysis of the potential ribosomal frame-shift sites in the sequence overlap regions of curioviruses and some hapaviruses. (PDF)

S1 Table Rhabdoviruses for which genome sequences have been used in this study. (PDF)

S2 Table Characteristics of possible animal rhabdovirus accessory proteins encoded in ORFs ≥ 180 nt. (PDF)

S1 Text Assignment of rhabdoviruses to existing genera and proposed new genera. (PDF)
