# Supplementary material for: Evolution of Genome Size and Complexity in the Rhabdoviridae
Source: PLoS Pathog. 2015 Feb 13;11(2):e1004664. doi: 10.1371/journal.ppat.1004664 (PMC4334499; doi:10.1371/journal.ppat.1004664)

Figure S1. ML phylogenetic tree of 100 rhabdovirus L protein sequences from Figure 2 with associated virus genome lengths. Where a genome sequence was incomplete (the first transcription initiation sequence to the final transcription termination sequence, inclusive) no genome length is shown. The scale bar indicates genome length in nucleotides.

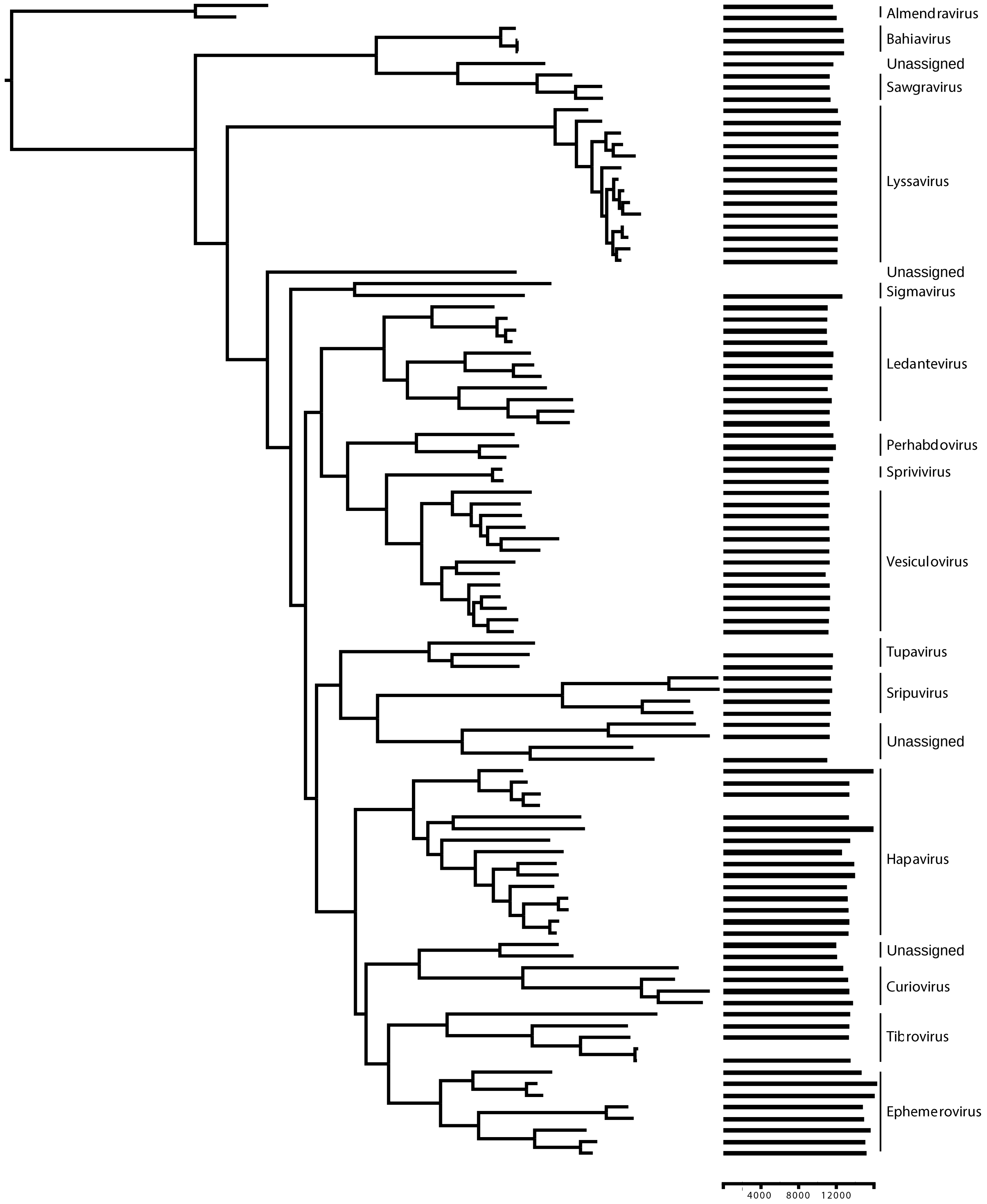

Supplement: S1 Fig — (PDF) [file ppat.1004664.s001.pdf]
