# Supplementary material for: Evolution of Genome Size and Complexity in the Rhabdoviridae
Source: PLoS Pathog. 2015 Feb 13;11(2):e1004664. doi: 10.1371/journal.ppat.1004664 (PMC4334499; doi:10.1371/journal.ppat.1004664)
Supplement: S2 Fig — (PDF) [file ppat.1004664.s002.pdf]

Figure S2. Amino acid sequence alignments (Clustal X) of various hapavirus PMIPs illustrating homologies that provide evidence for gene duplication. Identical amino acids are shaded.

A

```
WONV_U1     MEYQFLKKSSFGPQLDTVYVRDN------HMLFEGNLALIWEDEISERDLLLMLKEEISKFPNYQKYSSIYKIGVGILLSKSKYDFVWPD
WONV_U2     MATDENFDAKLAWVVESLDFSPSPRDDPVNFVISTKIDVDFPSSFDEVELLMHIRQEMKKNKEWTKSGSFMGLCAGIALSHSMLVPTEGL
WONV_U3     MATSDNPR-----LTRRVYFRLD----------------WYGDNLKDVDVVKLFKLTIESEEILDPIKEMIIEDLIKTCRKSFYDFVWPT
            *  :        : .  .                : ..:.: :::  ::  :..        .:          :*    .

WONV_U1     KSYLISGITDIINFPNIQRCPWDPQEDRIKIDTCGIWQGKRYNLSLNLYFSQADPRLGRPIWESWYSSFNSRPPFMRFEIETVSDYLGFG
WONV_U2     RKRLVGDFMGVLNIPLVPDQGTDYIILNTTSYNLDLNMWSEIKLSYTFFVCRGNGNVTKRIDTTWYANQPDRPEYLTFDLLTVSVLYGFD
WONV_U3     HHRLVGGSAFFGPCP-SELVDLLTDDVSIKLDETGMYNKSPFTLVMEVDYNLDEEANN---CESQNPN-NNRPSHLQF------------
            :  *:..   .   *                 .    .:   .  .*   .     :          :  ..  .** .: *

WONV_U1     ELVHG-------
WONV_U2     DWFVSPLVNYND
WONV_U3     ------------
```

B

```
ORV_U1      MEY------QFLKKISRGPQLDTVYVRDNHMLFEGNLAFIWEDDLSERELLLLLKSEISKLPNYQKYSSIYKVGIGLLLSKSKYDFVWPD
ORV_U2      MATSENFDSKLAWVVESLEFHPNPRDDPVNFVLSVKIDVDFPSDMDEINLLIHIRQELKKNKMWTQRGTFMGLCAGIGLSHSMFVPSDPL
ORV_U3      MAT---------------TNLNKRLTRRVYFRLD-----WYGDNLKDVDVIKLFKLTIESEDVLELVKDMMVEDLIRTCRKSFYDFVWPT
            *                  .        : :.      : .::.: :::  ::  :..          :          :* :    *

ORV_U1      KNYLISGTSDIVNFPNIQYCPWDPQEDRVRVDKCGLWQEKRYNLSLNLYFSYADPRLGRTIWESWYSELNSRPPFMRFEIETVSDYLGFG
ORV_U2      RRRLMGEFNGVLNIPLVPSVGDDYIILNTTSYNLDLESWSNIKLSYTFFICRGNGKVTKRIDTSWYSGQPARKEEYTFDLLTISVLYGFD
ORV_U3      HHRLVGGSAFFGPCP-ENLIELLKDDISIKLDETGMFRKSPFTLTMEIDYNLDED----TKECESQSPNNSRPPHLQF------------
            :. *:.    .   *                     : .:    .  .*:  :     :           .  *   :*     *

ORV_U1      ELVHH---------
ORV_U2      DWFVSPLVNYVEDP
ORV_U3      --------------
```

C

```
PCV_U1      MEYQFLKKTSRGPQLDTVYVRDN------HMLFEGNLVLIWEDDLTEREVLLLLKNEIVKFPNFKKYSNIYKLGVGLLLSKSKYDFVWPD
PCV_U2      MATNENFDSKLAWVVESLEFHPNPRDDPVNFVISARIDVEFPSNFDEVELLMHILQNLKRNKMWPQKGSFLGICAGLSLSHSSFVPSDPL
PCV_U3      MATTN----KNRRLTRRVYFRLD----------------WYGDNIRDVDVIKLFKLTIESEDLLEIVKDMMIEDLIKTCRKSFYDFVWPT
            *      ..       : .: :                : .:: : :::  :    :          .:          :* :    *

PCV_U1      KNYLISGMTDIVNFPNVQFCPWDPQEDRVKVDTCGTWQDKRFNLSLNLYFTYADPSLGRTIWESWYSELNGRPINMRFELETVSDYIGFG
PCV_U2      KKRLIGDFMGVVNIPLVPSVGNDYIILNTTSYNLDLESWSEIKLTYNFFICRGNGNVTKRIDTAWYGGQPKRGEDFTFDLLTVAVLYGFD
PCV_U3      HHRLVGGSAFFGPCP-EHLIDLLSDNISIKLDETGMFHKSPFTLIMEIDYNLDEDAN----ECESQSPNNNRPSHLQF------------
            :: *:..   .   *                 .    .    . :.*  ::     :                .    *  .: *

PCV_U1      ELVHM-------
PCV_U2      DWFASPLIDRDD
PCV_U3      ------------
```

D

```
FLAV_U1     MDLYINIGITFHYNNSYLDNDSLNWIISR-MIYDCVRESGIPGDVAAFAINMAWEHVDISFDSDKITHGYCWIQEAVSLPGTPKDLDKLN
FLAV_U2     MNLQIHGYISFVLPRS-SFTRRNLWCLQKGTVSELRRVAGITQDVSGVICSMLFSRLDFDLTEDDKIVSEVNLIESYMFPQRFNNVVNLH
FLAV_U3     MDCTFNLSAFFYLPD-YLYKKPVIDRLEWEIISWMRENYNMSTELCALTTTFLMSQVYPLAKDQDVLYLCGELRANVSFFKRSKHNQYPG
            *:  ::     *           .      :.   :     .  .:. ::...  .:  ..:      .::         :     :    :.

FLAV_U1     NTFSSRGNFFIQGDEALGEVEYVLFVAEPTLQEGR-PWNMLWHP----IFVDPQAYHVKRNPDVVAYKFGFQHLIYP-----
FLAV_U2     INVPSLKYTMVVEGKAVG-IHLMVRIDTVS-QIGRTLYTALWG---KRRVKNSMGRTLEEDGKKFGFQYLFE-MVYVPPPLN
FLAV_U3     IKWISENPSFVLGDYVCK-VSICGRVSFPGRPGGQTPWE-IWYSSTRSKIPKEMRREIEDAAHSYHFEYL---LDY------
             .  *    ::  . .    :      :       *.  :  :*        . .     ::.  .   :::     : *
```

E

```
HPV_U1      MDLYIHLGITFHYNGSYLDNDNLNWIISRMIYDCVRESGVPGDVAAFAINLAWEHVDICFDSGNITHGYCWLQEAVSLPGTPKDLDKLNN
HPV_U2      MNLQIHGYLSFILPRSIFTRRNLWCIQKGTVNELRRIAGITQDVSGLVCSMLFSRLDFDLTEDDKILSEVNLIENYMFPQRFNNVINLHI
HPV_U3      MNCTFNLSTFFYLPEYLYKKSVIDRLEWEIISWMRDNYNISTELCALTTTFLMSQVYPLTKDQDVLYLCGELRANVSFFKRSKHIQHPGI
            *:  ::     *           .  :  :     :     .   .:. ::..:. .:   .::      . :          *     :     :.* :

HPV_U1      TFSSRGSFFIQGDEDLGEVEYIFFVAEPTLQEGH-PWDVVWHP-MFVDPQAYHIKRNPDVVAYKFRFQHL-IHP---
HPV_U2      NVPSLKYTMVVEGKAVG-IHLILRIDTINQI-GRTLYTAMWGKRRMKNSMGRTLEEDGKRFGFQYLFEMVYIAPPLN
HPV_U3      KWISESPSFVLGDHVCK-VSICGRVSFPGRPGGQTPWEI-WYS-TIRSKIPKEM-----------------------
             .  *    ::  ..    :       :       *:  :   *     : .     :
```

F

```
MQOV_U1     M-EFFVSLGLSFSSTKKNITLEDIDLMIKKMVVRTHQIHGLPVDLGGLLINLLWEHTDFRTDCRGNLMGYGYLEASLSTPGTLVQVDLMD
MQOV_U2     MMNLHIIGRVEFSLPAALSLTPNIWKIQKQNVSEYRRLAGLTQDVAGLAMSFLYSKLRPRLIPGGLIAFVGDYNYSTRFPNRFANVRNLR
MQOV_U3     MI-LQIQLSIHVDVPAGKYDARYARRLAFYLVNRVAQENNIPRDIAGIAVSFLMSQVSLIHTSTDFDYLCGSIDVNLDIP---SNARAQV
            *  : :   : .  .               :    * .  :  .:. *:.*: :.:* .:         .      *  : .   *    :..
```

```
MQOV_U1     KTFCIHDGWEHDDLGIIDQVDYKVCVYPSPMEDPVPIMEY-WIPVDVSYG-NYLRRNPDIVANLYGFKHLISLCY------
MQOV_U2     STLQDSH-FIIQISGFNLDVKFSMSLFT--QETMIG-MDYHLVYGEDDYQTNMAHRTLDQDAKTFGFGYVMQVIPMPNFLN
MQOV_U3     PCLREL--ITINSPVFIGDETIHPTLWGSI---SYPALGTGVRPWEAWYTDRRCFIPSNLRLEIEDLAYDFKFEYILD---
              :        :   :   :       ::          :        :   *  .       :   .  .: . :..
```

G

```
MOSV_U1     MKLFINAGLTFNYYLPELPKDVLHEIISIMITRCVTMSGFPEDLAAFAVNWTWEQTQLCYYDFDVSHGYSWIQDDIDLPGDIKSDQPILK
MOSV_U2     MNLAISGSVKFVLPRESFTRRNLWLIQKASTTEYRRRAGITQDVAGMVCSFLFSKIKFRLTEEDKIECIGMACTCVMFPARFHNVFRLHI
MOSV_U3     MNLAINLSMSFKLQENWYTKEVIDRLEWEIISWMRDNHHLSTEVAAIVTTFLISQVSPLYLDNGEYYMTSELRVNVSFMKRSRHVQYPGT
            *:* *. .:.*         .:  :  :      :         . ::*.:. .:  .: .     : .      .      : :    :

MOSV_U1     EYRIRTEFPIGIDRNTGEVEFFIYINTQINRDAPHWSNVWWPKLTDPLANYLFRNPDQVASRYGFLHMLYT--------
MOSV_U2     QLRD-GRFTMVIDGKAIGVALSASLNTRNTMDGIRYEQEWG-EAYYPYSP-SGRTLEQDAKRFGFEYLFDMIQTPRVPN
MOSV_U3     TWIL-ESPLFSLAGKIIKVCFAGRITYPGGSGGQTPWETWY-TSVRSKIP---KELRREIEDAAHSYNFEYLLEP----
                      : :  :    * :   :.     ..    : *       .       :   :  .  .. : :
```

H

```
KAMV_U1     MKLFINAGLTFNYYLPELPKDVLHEIISIMITRCVTMSGFPEDLAAFAVNWTWEQTQLCYYDFDVSHGYSWIQDDIELPGDIKSDQPVLK
KAMV_U2     MNLAISGSVKFVLPRESFTRRNLWLIQKASTTEYRRRAGITQDVAGMVCSFLFSKIKFRLTEEDKIECIGMACTCVMFPARFHNVFRLHI
KAMV_U3     MNLAINLSMSFKLQENWYTKEVIDRLEWEIVSWMRDNHRLSTEVAAIVTTFLISQMSPLYLDGGEYYMTSELRVNLSFMRKSRHTQYPGT
            *:* *. .:.*         .:  :  :      :       :. ::*.:. .:  .: .     : .      .      : :    :

KAMV_U1     EYRIRTEFPIGIDRNTGEVEFFIYINTQINRDAPHWSSVWWPKLMDPLA-NYLFRNPDQVASKYGFLH---MLYT-----
KAMV_U2     QLRD-GRFTMVIDGKAIGVALSASLNTRNTMDGIRYEQEWG-EAYYPYSP--SGRTLEQDAKRFGFEYLFDMIQTPRVPN
KAMV_U3     TWIL-ENPLFSLAGKIIKVCFAGRITYPGGPGGQTPWETWY-TSVRSKIPRELRREIEDAAHSYNFEYLLEY--------
                   .  : :  :    * :   :.     ..    . *       .        *   :: *   :.* :
```

I

```
LJV_U1      MGSNI--YLKSMVFV---------IGPKPLTFDLLKFIIRKIPKISQDYQVNCLGKLAIGLAYQRSEFDVSKNSIQ--DGMFEGIIYFPW
LJV_U2      MDFNLDQEEKSHPLFFNVEIHIGYRGPYFDSNDLLTQIDKMIESRPSSSNHTDLIRLAIGLGLAHCVHSQITPTLWNLNGSFMGVLKLP-
LJV_U3      M--------STQKAYAFVSGHFEYKSFHFDKTEFCKALIDLIKTFTQDPVKVQWICLLSTIAALKGKILFKGDNTWGVRIYLECNLDLKG
            *                 :     ..   .   . :: . :   * . ...         *   :.  :         .        :   : :

LJV_U1      RSFPNLNSLDYSYKLRLTNYCDIYHIHIKIKASKSTERG-----FTIWETWDRCQN--NFLPDPSLEYSSDHLGFSDLIH
LJV_U2      -DYPVNPTVHTVVGQEITDGCGLLYPDISVYSKIYIMLGNESKVQDIITHWYTGEGGVCYEYDFSLPTVAGALKFEHLFL
LJV_U3      ---KWNLPRVEVIKEDYCSTGSPFMSQVCGWLAVNIKNGSR---PFLRKKHYSPDYREDHHSDISLGEFLRQAGLGGMIP
                .          .  .    .:          *        :        :     .  * **         :  ::
```

J

```
LJAV_U1     MDYFLHGGISIHKVPGSVTKEDIHYIMMKVINDLIHDLSMPHDLAGAAIALILSNVG-YRDMSDGSVEGEGYIQEGVSFTKPSQHPELCN
LJAV_U2     MNLDVNCVIRFELPKQLFTQSNLLCIEGAYVRYAQRNYRLNHDLSAIVINTAFSWLD-MWPKNDELVQCYAVLFTDFLIPRRLRGSRDYE
LJAV_U3     MDISVNLNLSFDIPTGLYSKVVLDRLEWEIITWLKDNYRVSVEISAIVINLLFAHIYPIGPPVNHILTLQSKIHDVVSFQK--RSLRQQY
            *:  ::  : :.       :.  :  :     :     :  :  :::. .*   :: :        :  :    . :    . . :  :  .

LJAV_U1     RNWEHYGHHYINREDGSFFEYFI--FMSRPLIFIGKPYIELWSCITFPHNYHKMTLSPDLLALEYDFAHMICHM-------
LJAV_U2     IDWRIDNFN--IKLKETNVPLHLHLGIRTPMMPSATMISEVWGKRGRNPMPLKKEWSLAFKAEVYRWDYIFNVLQDPIILN
LJAV_U3     IGRQVINADPFFRLDGYFCEVKIRGFVTYPSPTIGRRIWELWYGPPREKVPITMKREIEDSAEKYNWMYLIEYW-------
                 .  .  .   : .        :   :   *    .    *:*                  *   * : :::
```

K

```
MANV_U1     METFLHGGITLFCNSILVPKTLLHEIILKITNDLVHDCGMPPDLASAISTLLLSNTLFKYLSDGTVEGTGFIQDGVSYKGETKWIDQKGKN
MANV_U2     MDLNVGCYIDFTFPNNLFDLKTLELIEGACIRWCQRHINLNKDLIAIIINLAFSQVEFRPAQNQKVYGRSEVNNYLELQNFLRGSKDYFID
MANV_U3     MNISAGVSLSFNLPKELYKKEVLDRLEWNLVLWFKDTYHVSVEIASIIITLLFARLYPQYTEDNMVHLVSEIHDIISFDHRSRREQYPGTA
            *:       : :    .          *   :                :    :  . *    ::.        .    *    . ::: :. .

MANV_U1     WNKTGIHDLTDVSGSFFEYFIFCSEPAIFEGKPFEELWATQDSHHPYFKQKMNLDVYSFEYGFNHLLHD--------
MANV_U2     WAKHDFTMKLTSAEIPIHINFYILPYRGGLLRTLDQAWGRNYKSTMLRIPEWQLEYKAHLYNYSYMLTRSTELPELN
MANV_U3     LIGEKAMFKLDFYWCTINMGGFVTYPSPISGKKIWELWYGDHRRHIKPALRREIEDASEKYNYVYLIEYW-------
                              :.             :  . *                 ::.   :    .:    :
```
