# Supplementary material for: Evolution of Genome Size and Complexity in the Rhabdoviridae
Source: PLoS Pathog. 2015 Feb 13;11(2):e1004664. doi: 10.1371/journal.ppat.1004664 (PMC4334499; doi:10.1371/journal.ppat.1004664)
Supplement: S3 Fig — (PDF) [file ppat.1004664.s003.pdf]

Figure S3. Amino acid sequence alignments (Clustal X) of various hapavirus PMIPs that illustrate homologies between proteins from different viruses, providing evidence that duplicated genes share a common ancestor. Identical amino acids are shaded.

A

```
WONV_U1    MEYQFLKKSSFGPQLDTVYVRDN------HMLFEGNLALIWEDEISERDLLLMLKEEISKFPNYQKYSSIYKIGVGILLSKSKYDFVWPD
ORV_U2     MATSENFDSKLAWVVESLEFHPNPRDDPVNFVLSVKIDVDFPSDMDEINLLIHIRQELKKNKMWTQRGTFMGLCAGIGLSHSMFVPSDPL
PCV_U2     MATNENFDSKLAWVVESLEFHPNPRDDPVNFVISARIDVEFPSNFDEVELLMHILQNLKRNKMWPQKGSFLGICAGLSLSHSSFVPSDPL
           *  .   .*.:.  :::: .: *      ::::. .: : : .::.* :**: : :::.:   : : .::  : .*: **:* :    *

WONV_U1    KSYLISGITDIINFPNIQRCPWDPQEDRIKIDTCGIWQGKRYNLSLNLYFSQADPRLGRPIWESWYSSFNSRPPFMRFEIETVSDYLGFG
ORV_U2     RRRLMGEFNGVLNIPLVPSVGDDYIILNTTSYNLDLESWSNIKLSYTFFICRGNGKVTKRIDTSWYSGQPARKEEYTFDLLTISVLYGFD
PCV_U2     KKRLIGDFMGVVNIPLVPSVGNDYIILNTTSYNLDLESWSEIKLTYNFFICRGNGNVTKRIDTAWYGGQPKRGEDFTFDLLTVAVLYGFD
           :  *:. : .::*:* :     *    . .  . .: . .. :*: .:::.:.: .: : *  :**..   *     *:: *::   **.

WONV_U1    ELVHG---------
ORV_U2     DWFVSPLVNYVEDP
PCV_U2     DWFASPLIDRDD--
           : . .
```

B

```
WONV_U2    MATDENFDAKLAWVVESLDFSPSPRDDPVNFVISTKIDVDFPSSFDEVELLMHIRQEMKKNKEWTKSGSFMGLCAGIALSHSMLVPTEGL
ORV_U1     M--EYQFLKKISRGPQ-LDT-VYVRDN--HMLFEGNLAFIWEDDLSERELLLLLKSEISKLPNYQKYSSIYKVGIGLLLSKSKYDFVWPD
PCV_U1     M--EYQFLKKTSRGPQ-LDT-VYVRDN--HMLFEGNLVLIWEDDLTEREVLLLLKNEIVKFPNFKKYSNIYKLGVGLLLSKSKYDFVWPD
           *  : :*  * :   : **     **:  ::::. :: . : ..: * *:*: ::.*: *  :: * ..:  :  *: **:*    .

WONV_U2    RKRLVGDFMGVLNIPLVPDQGTDYIILNTTSYNLDLNMWSEIKLSYTFFVCRGNGNVTKRIDTTWYANQPDRPEYLTFDLLTVSVLYGFD
ORV_U1     KNYLISGTSDIVNFPNIQYCPWDPQEDRVRVDKCGLWQEKRYNLSLNLYFSYADPRLGRTIWESWYSELNSRPPFMRFEIETVSDYLGFG
PCV_U1     KNYLISGMTDIVNFPNVQFCPWDPQEDRVKVDTCGTWQDKRFNLSLNLYFTYADPSLGRTIWESWYSELNGRPINMRFELETVSDYIGFG
           :: *:..  .::*:* :     *    ..   . .    .. :** .::.  .:  : : *  :**::  .**  : *:: ***   **.

WONV_U2    DWFVSPLVNYND
ORV_U1     ELVHH-------
PCV_U1     ELVHM-------
           : .
```

C

```
WONV_U3    MAT------SDNPRLT----RRVYFRLD-----WYGDNLKDVDVVKLFKLTIESEEILDPIKEMIIEDLIKTCRKSFYDFVWPTHHRLVG
ORV_U1     MEYQFLKKISRGPQLDTVYVRDNHMLFEGNLAFIWEDDLSERELLLLLKSEISKLPNYQKYSSIYKVGIGLLLSKSKYDFVWPDKNYLIS
PCV_U1     MEYQFLKKTSRGPQLDTVYVRDNHMLFEGNLVLIWEDDLTEREVLLLLKNEIVKFPNFKKYSNIYKLGVGLLLSKSKYDFVWPDKNYLIS
           *        * .*:*     *  :: ::      : *:*.: ::: *:*  * .    .  ..:   .:     ** ****** :: *:.

WONV_U3    GSAFFGPCP-SELVDLLTDDVSIKLDETGMYNKSPFTLVMEVDYNL-DEEANN--CESQNPNNN-RPSHLQF-----------------
ORV_U1     GTSDIVNFPNIQYCPWDPQEDRVRVDKCGLWQEKRYNLSLNLYFSYADPRLGRTIWESWYSELNSRPPFMRFEIETVSDYLGFGELVHH
PCV_U1     GMTDIVNFPNVQFCPWDPQEDRVKVDTCGTWQDKRFNLSLNLYFTYADPSLGRTIWESWYSELNGRPINMRFELETVSDYIGFGELVHM
           * : :   *  :     .::  :::*  * ::.. :.* ::: :.  :   .    **  .:  **  ::*                *
```

D

```
WONV_U1    MEYQFLKKSSFGPQLDTVYVRDNHMLFEGNLALIWEDEISERDLLLMLKEEISKFPNYQKYSSIYKIGVGILLSKSKYDFVWPDKSYLIS
ORV_U3     MAT---------TNLNKRLTRRVYFRLD-----WYGDNLKDVDVIKLFKLTIESEDVLELVKDMMVEDLIRTCRKSFYDFVWPTHHRLVG
PCV_U3     MAT---------TNKNRRLTRRVYFRLD-----WYGDNIRDVDVIKLFKLTIESEDLLEIVKDMMIEDLIKTCRKSFYDFVWPTHHRLVG
           *           .: :   .*  :: ::      : *:: : *:: ::*  *..    :  ..:   .:     ** ****** :  *:.

WONV_U1    GITDIINFPNIQRCPWDPQEDRIKIDTCGIWQGKRYNLSLNLYFSQADPRLGRPIWESWYSSFNSRPPFMRFEIETVSDYLGFGELVHG
ORV_U3     GSAFFGPCP-ENLIELLKDDISIKLDETGMFRKSPFTLTMEIDYNLDEDTK---ECESQ-SPNNSRPPHLQF-----------------
PCV_U3     GSAFFGPCP-EHLIDLLSDNISIKLDETGMFHKSPFTLIMEIDYNLDEDAN---ECESQ-SPNNNRPSHLQF-----------------
           * : :   *  :      ::  **:*  *::: . :.* ::: :.  :        **  *. *.**..::*
```

E

```
PCV_U1     MEYQFLKKTSRGPQLDTVYVRDNHMLFEGNLVLIWEDDLTEREVLLLLKNEIVKFPNFKKYSNIYKLGVGLLLSKSKYDFVWPDKNYLIS
ORV_U3     M-----ATTN----LNKRLTRRVYFRLD-----WYGDNLKDVDVIKLFKLTIESEDVLELVKDMMVEDLIRTCRKSFYDFVWPTHHRLVG
           *    : .*.    *:.  .*  :: ::      : *:*.: :*: *:*  * .   ::  .::   .:     ** ****** :: *:.

PCV_U1     GMTDIVNFPNVQFCPWDPQEDRVKVDTCGTWQDKRFNLSLNLYFTYADPSLGRTIWESWYSELNGRPINMRFELETVSDYIGFGELVHM
ORV_U3     GSAFFGPCP-ENLIELLKDDISIKLDETGMFRKSPFTLTMEIDYNLDEDTKECESQS----PNNSRPPHLQF-----------------
           * : :   *  ::     ::  :*:*  * ::.. *.*:::: :.  : :      .      *.** :::*
```

F

```
PCV_U1     MEYQFLKKTSRGPQLDTVYVRDN------HMLFEGNLVLIWEDDLTEREVLLLLKNEIVKFPNFKKYSNIYKLGVGLLLSKSKYDFVWPD
ORV_U2     MATSENFDSKLAWVVESLEFHPNPRDDPVNFVLSVKIDVDFPSDMDEINLLIHIRQELKKNKMWTQRGTFMGLCAGIGLSHSMFVPSDPL
           *  .   .:. .  :::: .: *      ::::. :: : : .*: * ::*: :::*: *   :.: ..:  * .*: **:* :    *

PCV_U1     KNYLISGMTDIVNFPNVQFCPWDPQEDRVKVDTCGTWQDKRFNLSLNLYFTYADPSLGRTIWESWYSELNGRPINMRFELETVSDYIGFG
ORV_U2     RRRLMGEFNGVLNIPLVPSVGDDYIILNTTSYNLDLESWSNIKLSYTFFICRGNGKVTKRIDTSWYSGQPARKEEYTFDLLTISVLYGFD
           :. *:. :..::*:* *     *    ...  . .  . ..::** .:::  .: .: : *  ****   .*  :  *:* *:*   **.

PCV_U1     E-----LVHM----
ORV_U2     DWFVSPLVNYVEDP
           :     **.
```

G

```
PCV_U2     MATNENFDSKLAWVVESLEFHPNPRDDPVNFVISARIDVEFPSNFDEVELLMHILQNLKRNKMWPQKGSFLGICAGLGLSHSSFVPSDPL
ORV_U2     MATSENFDSKLAWVVESLEFHPNPRDDPVNFVLSVKIDVDFPSDMDEINLLIHIRQELKKNKMWTQRGTFMGLCAGIGLSHSMFVPSDPL
PCV_U3     MATTNKNRRLTRRVYFRLDWYGDN-------IRDVDVIKLFKLTIESEDLLEIVKDMMIEDLIKTCRKSFYDFVW-------------PT
ORV_U3     MATTNLNKRLTRRVYFRLDWYGDN-------LKDVDVIKLFKLTIESEDVLELVKDMMVEDLIRTCRKSFYDFVW-------------PT
           ***.:        *   *::: :        :  ..:   *   ::. ::*  : : : .: : . : :* .:               *

PCV_U2     KKRLIG--DFMGVVNIPLVPSVGNDYIILNTTSYNLDLESWSEIKLTYNFFICRGNGNVTKRIDTAWYGGQPKRGEDFTFDLLTVAVLYG
ORV_U2     RRRLMG--EFNGVLNIPLVPSVGDDYIILNTTSYNLDLESWSNIKLSYTFFICRGNGKVTKRIDTSWYSGQPARKEEYTFDLLTISVLYG
PCV_U3     HHRLVGGSAFFGPCPEHLIDLLSDN---------------IS-IKLDETGMFHKSPFTLIMEIDYNLDEDANECESQSPNNNRPSHLQF-
ORV_U3     HHRLVGGSAFFGPCPENLIELLKDD---------------IS-IKLDETGMFRKSPFTLTMEIDYNLDEDTKECESQSPNNSRPPHLQF-
           ::**:*   * *     *:  : ::                *.***  . :: :.  .:  .**     .     .: . :  .  : :

PCV_U2     FDDWFASPLIDRDD--
ORV_U2     FDDWFVSPLVNYVEDP
PCV_U3     ----------------
ORV_U3     ----------------
```

H

```
LJAV_U3    MDISVNLNLSFDIPTGLYSKVVLDRLEWEIITWLKDNYRVSVEISAIVINLLFAHIYPIGPPVNHILTLQSKIHDVVSFQKRSLRQQYIG
MANV_U3    MNISAGVSLSFNLPKELYKKEVLDRLEWNLVLWFKDTYHVSVEIASIIITLLFARLYPQ-YTEDNMVHLVSEIHDIISFDHRSRREQYPG
           *:**..:.***::*. **.* *******::: *:**.*:*****::*:*.****::**   . :::: * *:***::**::** *:** *

LJAV_U3    RQVINADPFFRLDGYFCEVKIRGFVTYPSPTIGRRIWELWYGPPREKVPITMKREIEDSAEKYNWMYLIEYW
MANV_U3    TALIGEKAMFKLDFYWCTINMGGFVTYPSPISGKKIWELWYGDHRRHIKPALRREIEDASEKYNYVYLIEYW
             :*. ..:*:** *:* ::: ********  *::*******  *.::  :::*****::****::******
```

I

```
LJAV_U2    MNLDVNCVIRFELPKQLFTQSNLLCIEGAYVRYAQRNYRLNHDLSAIVINTAFSWLDMWPKNDELVQCYAVLFTDFLIPRRLRGSRDYEI
MANV_U3    MNISAGVSLSFNLPKELYKKEVLDRLEWNLVLWFKDTYHVSVEIASIIITLLFARLYPQYTEDNMVHLVSEIHDIISFDHRSRREQYPGT
           **:...  : *:***:*:.:. *  :*   * : : .*::. ::::*:*.  *: *    .:*::*:  : :.  : : :* * .:

LJAV_U2    DWRIDNFNIKLKETNVPLHLHLGIRTPMMPSATMISEVWGKRGRNPMPLKKEWSLAFKAEVYRWDYIFNVLQDPIILN
MANV_U3    ALIGEKAMFKLDFYWCTINMGGFVTYPSPISGKKIWELWYGDHRRHIKPALRREIEDASEKYNYVYLIEYW-------
               ::  :**.    .:::   :  *   *.. * *:*    *. :    . .:   :* *.: *:::
```

J

```
LJAV_U2    M------NLDVNCVIR-----------FELPKQLFTQSNLLCIEGAYVRYAQRNYRLNHDLSAIVINTAFSWLDMWPKNDELVQCYAVLF
MANV_U1x   MFILLSTDLIISCMIKMDFYYINLYLEFSLPKSAYCYAALIHIEKRLQDIISKKYKI---LSGIISGWAISNLEVVPGNYGLVECQSIIQ
           *      :* :.*:*:           *.***. :  : *: **       .::*::   **.*: . *:* *:: * *  **:* :::

LJAV_U2    TDFLIPRRLRGSRDYEIDWRIDNFNIKLKETNVPLHLHLGIRTPMMPSATMISEVWGKRGRNPMPLKKEWSLAFKAEVYRW-DYIFNVLQ
MANV_U1x   DAIQIKFDPTSVCNDLVVNK--RFELKVLQWVIPIKLLLEVNRSHFPIGSRLTSLWGRRGDSFMIHDEMWDIKTVANRLGFADAI-----
             : *     .  :  :  :  .*::*: :  :*::* * :. . :* .: ::.:**:** . *  .: *.:   *:   : * *

LJAV_U2    DPIILN
MANV_U1x   ------
```

K

```
LJAV_U3    MDISVNLNLSFDIPTGLYSKVVLDRLEWEIITWLKDNYRVSVEISAIVINLLFAHIYPIGPPVNHILTLQSKIHDVVSFQ--KRSLRQQY
MANV_U1    METFLHGGITLFCNSILVPKTLLHEIILKITNDLVHDCGMPPDLASAISTLLLSNTL-FKYLSDGTVEGTGFIQDGVSYKGETKWIDQKG
           *:  :: .:::   : * .*.:*..:  :* . * .:  :. :::: : .**:::   :    :  :   . *:* **::  .: : *:

LJAV_U3    IGRQVINADPFFRLDGYFCEVKIRGFVTYPSPTIGRRIWELWYGPPREKVPITMKREIEDSAEKYNWMYLIEYW
MANV_U1    KNWNKTGIHDLTDVSGSFFEYFI--FCSEPAIFEGKPFEELWATQDSHHPYFKQKMNLDVYSFEYGFNHLLHD-
            . :  . . :  :.* * *  *  * : *:   *: : ***     .:  :. * :::  : :*.: :*:.
```

L

```
LJAV_U1    MDYFLHGGISIHKVPGSVTKEDIHYIMMKVINDLIHDLSMPHDLAGAAIALILSNVGYRDMSDGSVEGEGYIQEGVSFTKPSQHPELCNR
HPV_U1     MDLYIHLGITFHYNGSYLDNDNLNWIISRMIYDCVRESGVPGDVAAFAINLAWEHVDICFDSGNITHGYCWLQEAVSLPGTPKDLDKLNN
MOSV_U1    MKLFINAGLTFNYYLPELPKDVLHEIISIMITRCVTMSGFPEDLAAFAVNWTWEQTQLCYYDFDVSHGYSWIQDDIDLPGDIKSDQPILK
KAMV_U1    MKLFINAGLTFNYYLPELPKDVLHEIISIMITRCVTMSGFPEDLAAFAVNWTWEQTQLCYYDFDVSHGYSWIQDDIELPGDIKSDQPVLK
FLAV_U1    MDLYINIGITFHYNNSYLDNDSLNWIISRMIYDCVRESGIPGDVAAFAINMAWEHVDISFDSDKITHGYCWIQEAVSLPGTPKDLDKLNN
MQOV_U1    MEFFVSLGLSFSSTKKNITLEDIDLMIKKMVVRTHQIHGLPVDLGGLLINLLWEHTDFRTDCRGNLMGYGYLEASLSTPGTLVQVDLMDK
           *. ::  *:::      :  : :. ::  ::       ..* *:..  :    .:.           *  *::  :. .      :   .

LJAV_U1    NWEHYGH-HYINREDGS-FFEYFIFMSRPLIFIGKPYIELWSCITFPHNYHKMTLSPDLLALEYDFAHMICHM-
HPV_U1     TFSSRGS-FFIQGDEDLGEVEYIFFVAEPTLQEGHPWDVVWHPMFVDPQAYHIKRNPDVVAYKFRFQHLIHP--
MOSV_U1    EYRIRTE-FPIGIDRNTGEVEFFIYINTQINRDAPHWSNVWWPKLTDPLANYLFRNPDQVASRYGFLHMLYT--
KAMV_U1    EYRIRTE-FPIGIDRNTGEVEFFIYINTQINRDAPHWSSVWWPKLMDPLANYLFRNPDQVASKYGFLHMLYT--
FLAV_U1    TFSSRGN-FFIQGDEALGEVEYVLFVAEPTLQEGRPWNMLWHPIFVDPQAYHVKRNPDVVAYKFGFQHLIYP--
MQOV_U1    TFCIHDG-WEHDDLGIIDQVDYKVCVYPSPMEDPVPIMEYWIPVDV-SYGNYLRRNPDIVANLYGFKHLISLCY
            :         . .    :.:: .            :   *           :  .** :*  : * *::
```

M

```
MANV_U2    MDLNVGCYIDFTFPNNLFDLKTLELIEGACIRWCQRHINLNKDLIAIIINLAFSQVEFRPAQNQKVYGRSEVNNYLELQNFLRGSKDYFI
HPV_U3     MNCTFNLSTFFYLPEYLYKKSVIDRLEWEIISWMRDNYNISTELCALTTTFLMSQVYPLTKDQDVLYLCGELRANVSFFKRSKHIQHPGI
FLAV_U3    MDCTFNLSAFFYLPDYLYKKPVIDRLEWEIISWMRENYNMSTELCALTTTFLMSQVYPLAKDQDVLYLCGELRANVSFFKRSKHNQYPGI
MOSV_U3    MNLAINLSMSFKLQENWYTKEVIDRLEWEIISWMRDNHHLSTEVAAIVTTFLISQVSPLYLDNGEYYMTSELRVNVSFMKRSRHVQYPGT
KAMV_U3    MNLAINLSMSFKLQENWYTKEVIDRLEWEIVSWMRDNHRLSTEVAAIVTTFLISQMSPLYLDGGEYYMTSELRVNLSFMRKSRHTQYPGT
           *:  ..    * : :  :   .:: :*   : * : : .:..:: *:  .: :**:     :    *  .*:.  :.: .  :  :

MANV_U2    DWAKHDFTMKLTSAEIPIHINFYILPYRGGLLRTLDQAWGRNYKSTMLRIPEWQLEYKAHLYNYSYMLTRSTELPELN------------
```

```
HPV_U3     KWISESPSFVLGDHVCKVSICGRVSFPGRPGGQTPWEIWYSTIRSKIPKEMRREIEDAAHSYNFEYLLDY--------------------
FLAV_U3    KWISENPSFVLGDYVCKVSICGRVSFPGRPGGQTPWEIWYSSTRSKIPKEMRREIEDAAHSYHFEYLLDY--------------------
MOSV_U3    TWILESPLFSLAGKIIKVCFAGRITYPGGSGGQTPWETWYTSVRSKIPKELRREIEDAAHSYNFEYLLEPGVRPWEAWYTDRRCFIPSNL
KAMV_U3    TWILENPLFSLAGKIIKVCFAGRITYPGGPGGQTPWETWYTSVRSKIPRELRREIEDAAHSYNFEYLLEY--------------------
            *  ..  : * .    : :   :        :*  : *  . :*.: :  . ::*  ** *::.*:*

MANV_U2    ------------------
HPV_U3     ------------------
FLAV_U3    ------------------
MOSV_U3    RLEIEDLAYDFKFEYILD
KAMV_U3    ------------------
```

N

```
MANV_U3    M-NISAGVSLSFNLPKELYKKEVLDRLEWNLVLWFKDTYHVSVEIASIIITLLFARLYPQYTEDNMVHLVSEIHDIISFDHRSRREQYPG
HPV_U2     M-NLQIHGYLSFILPRSIFTRRNLWCIQKGTVNELRRIAGITQDVSGLVCSMLFSRLDFDLTEDDKILSEVNLIENYMFPQRFNNVINLH
FLAV_U2    M-NLQIHGYISFVLPRSSFTRRNLWCLQKGTVSELRRVAGITQDVSGVICSMLFSRLDFDLTEDDKIVSEVNLIESYMFPQRFNNVVNLH
MOSV_U2    M-NLAISGSVKFVLPRESFTRRNLWLIQKASTTEYRRRAGITQDVAGMVCSFLFSKIKFRLTEEDKIECIGMACTCVMFPARFHNVFRLH
KAMV_U2    M-NLAISGSVKFVLPRESFTRRNLWLIQKASTTEYRRRAGITQDVAGMVCSFLFSKIKFRLTEEDKIECIGMACTCVMFPARFHNVFRLH
MQOV_U2    MMNLHIIGRVEFSLPAALSLTPNIWKIQKQNVSEYRRLAGLTQDVAGLAMSFLYSKLRPRLIPGGLIAFVGDYNYSTRFPNRFANVRNLR
           * *:     :.* **        :  ::   .   :    :: :::.:  ::*::::       . :           *  *  .

MANV_U3    TALIGEKAMFKLDFYWCTINMGGFVTYPSPISGKKIWELWYGDHRRHIKPALRREIEDASEKYNYVYLIEYW-------
HPV_U2     INVPSLKYTMVVEGKAVGIHLILRIDTINQIGR-TLYTAMWGKRRMKNSMG--RTLEEDGKRFGFQYLFEMVYIAPPLN
FLAV_U2    INVPSLKYTMVVEGKAVGIHLMVRIDTVSQIGR-TLYTALWGKRRVKNSMG--RTLEEDGKKFGFQYLFEMVYVPPPLN
MOSV_U2    IQLRDGRFTMVIDGKAIGVALSASLNTRNTMDG-IRYEQEWGEAYYPYSPSG-RTLEQDAKRFGFEYLFDMIQTPRVPN
KAMV_U2    IQLRDGRFTMVIDGKAIGVALSASLNTRNTMDG-IRYEQEWGEAYYPYSPSG-RTLEQDAKRFGFEYLFDMIQTPRVPN
MQOV_U2    STLQDSHFIIQISGFNLDVKFSMSLFTQETMIG-MDYHLVYGEDDYQTNMAH-RTLDQDAKTFGFGYVMQVIPMPNFLN
             : . :  : :.     : :   :   . :     :   :*.     . .  * ::: .: :.: *:::
```

O

```
MANV_U3    MNISAGVSLSFNLPKELYKKEVLDRLEWNLVLWFKDTYHVSVEIASIIITLLFARLYPQYTEDNMVHLVSEIHDIISFDHRSRREQYPGT
HPV_U3     MNCTFNLSTFFYLPEYLYKKSVIDRLEWEIISWMRDNYNISTELCALTTTFLMSQVYPLTKDQDVLYLCGELRANVSFFKRSKHIQHPGI
FLAV_U3    MDCTFNLSAFFYLPDYLYKKPVIDRLEWEIISWMRENYNMSTELCALTTTFLMSQVYPLAKDQDVLYLCGELRANVSFFKRSKHNQYPGI
MOSV_U3    MNLAINLSMSFKLQENWYTKEVIDRLEWEIISWMRDNHHLSTEVAAIVTTFLISQVSPLYLDNGEYYMTSELRVNVSFMKRSRHVQYPGT
KAMV_U3    MNLAINLSMSFKLQENWYTKEVIDRLEWEIVSWMRDNHRLSTEVAAIVTTFLISQMSPLYLDGGEYYMTSELRVNLSFMRKSRHTQYPGT
MQOV_U3    MILQIQLSIHVDVPAGKYDARYARRLAFYLVNRVAQENNIPRDIAGIAVSFLMSQVSLIHTSTDFDYLCGSIDVNLDIPSNARAQVPCLR
           *     :*  . :    *      ** : ::  . :  .:. ::..:  ::*::::     . .  :: ..:   :.:  .::

MANV_U3    ALIGEKAMFKLDFYWCTINMGGFVTYPSPISGKKIWELWYGDHRRHIKPALRREIEDASEKYNYVYLIEYW
HPV_U3     KWISESPSFVLGDHVCKVSICGRVSFPGRPGGQTPWEIWYSTIRSKIPKEMRREIEDAAHSYNFEYLLDY-
FLAV_U3    KWISENPSFVLGDYVCKVSICGRVSFPGRPGGQTPWEIWYSSTRSKIPKEMRREIEDAAHSYHFEYLLDY-
MOSV_U3    TWILESPLFSLAGKIIKVCFAGRITYPGGSGGQTPWETWYTSVRSKIPKELRREIEDAAHSYNFEYLLEP-
KAMV_U3    TWILENPLFSLAGKIIKVCFAGRITYPGGPGGQTPWETWYTSVRSKIPRELRREIEDAAHSYNFEYLLEY-
MQOV_U3    ELITINSPVFIGDETIHPTLWGSISYPALGTGVRPWEAWYTDRRCFIPSNLRLEIEDLAYDFKFEYILD--
             *  .. . :        : * :::*.   *   ** **   *  *   :* **** : .::: *:::
```

P

```
MANV_U2    MDLNVGCYIDFTFPNNLFDLKTLELIEGACIRWCQRHINLNKDLIAIIINLAFSQVEFRPAQNQKVYGRSEVNNYLELQNFLRGSKDYFI
HPV_U1     MDLYIHLGITFHYNGSYLDNDNLNWIISRMIYDCVRESGVPGDVAAFAINLAWEHVDICFDSGNITHGYCWLQEAVSLPGTPKDLDKLNN
FLAV_U1    MDLYINIGITFHYNNSYLDNDSLNWIISRMIYDCVRESGIPGDVAAFAINMAWEHVDISFDSDKITHGYCWIQEAVSLPGTPKDLDKLNN
           *** :   * * : .. :* ..*: * .  *  * *. .:  *: *: **:*:.:*::   ..: .:* . ::: :.* .  :. ..

MANV_U2    DWAKHDFTMKLTSAEIPIHINFYILPYRGGLLRTLDQAWGRNYKSTMLRIPEWQLEYKAHLYNYSYMLTRSTELPELN
HPV_U1     TFSSRG-SFFIQGDEDLGEVEYIFFVAEPTLQE--GHPWDVVWHPMFVDPQAYHIKRNPDVVAYKFRFQHLIHP----
FLAV_U1    TFSSRG-NFFIQGDEALGEVEYVLFVAEPTLQE--GRPWNMLWHPIFVDPQAYHVKRNPDVVAYKFGFQHLIYP----
            ::.:. .: : . *   .::: ::  .  * .  .:.*.  ::. ::    :::: :..:  *.: : :
```

Q

```
MANV_U1    METFLHGGITLFCNSILVPKTLLHEIILKITNDLVHDCGMPPDLASAISTLLLSNTLFKYLSDGTVEGTGFIQDGVSYKGETKWIDQKGK
MOSV_U3    MNLAINLSMSFKLQENWYTKEVIDRLEWEIISWMRDNHHLSTEVAAIVTTFLISQVSPLYLDNGEYYMTSELRVNVSFMKRSRHVQYPGT
           *:  :: .:::  :.   .* ::..:  :* . : .:  :..::*: ::*:*:*:.   **.:*    *. :: .**:  .::  ::  *.

MANV_U1    NWNKTGIHDLTDVSGSFFEYFIF--CSEPAIFEGKPFEELWATQ-DSHHPYFKQKMNLDVYSFEYGFNHLLHD
MOSV_U3    TWILESP--LFSLAGKIIKVCFAGRITYPGGSGGQTPWETWYTSVRSKIPKELRREIED-AAHSYNFEYLLEP
           .*   .   * .::*.:::  :    : *.   *:.  * * *.  *: * : : :::* :..*.*::**.
```

R

```
MANV_U2    MD----------------------------LNVGCYIDFTFPNNLFDLKTLELIEGACIRWCQRHINLNKDLIAIIINLAFSQVEFRPAQ
ORV_U2     MATSENFDSKLAWVVESLEFHPNPRDDPVNFVLSVKIDVDFP-SDMDEINLLIHIRQELKKNKMWTQR-GTFMGLCAGIGLSHSMFVPSD
PCV_U2     MATNENFDSKLAWVVESLEFHPNPRDDPVNFVISARIDVEFP-SNFDEVELLMHILQNLKRNKMWPQK-GSFLGICAGLSLSHSSFVPSD
WONV_U2    MATDENFDAKLAWVVESLDFSPSPRDDPVNFVISTKIDVDFP-SSFDEVELLMHIRQEMKKNKEWTKS-GSFMGLCAGIALSHSMLVPTE
LJV_U2     MD-----------------FNLDQEEKSHPLFFNVEIHIGYRGPYFDSNDLLTQIDKMIESRPSSSNH-TDLIRLAIGLGLAHCVHSQIT
           *                             : ..  *.. :    :*   *       :.      :    :: :  .:.:::

MANV_U2    NQKVYGRSEVNNYLELQNFLRGSKDYFIDWAKHDFTMKLTSAEIPIHINFYILPYRGGLLRTLDQAWGRNYKSTMLRIPEWQLEYKAHLY
ORV_U2     PLRRRLMGEFNGVLNIPLVPSVGDDYIILNTTSYNLDLESWSNIKLSYTFFICRGNGKVTKRIDTSWYSG-QPARKEEYTFDLLTISVLY
PCV_U2     PLKKRLIGDFMGVVNIPLVPSVGNDYIILNTTSYNLDLESWSEIKLTYNFFICRGNGNVTKRIDTAWYGG-QPKRGEDFTFDLLTVAVLY
WONV_U2    GLRKRLVGDFMGVLNIPLVPDQGTDYIILNTTSYNLDLNMWSEIKLSYTFFVCRGNGNVTKRIDTTWYAN-QPDRPEYLTFDLLTVSVLY
LJV_U2     PTLWNLNGSFMGVLKLPDYPVNPTVHTVVGQEITDGCGLLYPDISVYSKIYIMLGNESKVQDIITHWYTG-EGGVCYEYDFSLPTVAGAL
                  ... . :::         : :             .:* :  .:::   .    : :   *  . :        :.*   :
```

S

```
LJAV_U2    MNLDVNCVIRFELPKQLFTQSNLLCIEGAYVRYAQRNYRLNHDLSAIVINTAFSWLDMWPKNDELVQCYAVLFTDFLIPRRLRGSRDYEI
MOSV_U3    MNLAINLSMSFKLQENWYTKEVIDRLEWEIISWMRDNHHLSTEVAAIVTTFLISQVSPLYLDNGEYYMTSELRVNVSFMKRSRHVQYPGT
KAMV_U3    MNLAINLSMSFKLQENWYTKEVIDRLEWEIVSWMRDNHRLSTEVAAIVTTFLISQMSPLYLDGGEYYMTSELRVNLSFMRKSRHTQYPGT
HPV_U3     MNCTFNLSTFFYLPEYLYKKSVIDRLEWEIISWMRDNYNISTELCALTTTFLMSQVYPLTKDQDVLYLCGELRANVSFFKRSKHIQHPGI
FLAV_U3    MDCTFNLSAFFYLPDYLYKKPVIDRLEWEIISWMRENYNMSTELCALTTTFLMSQVYPLAKDQDVLYLCGELRANVSFFKRSKHNQYPGI
MANV_U3    MNISAGVSLSFNLPKELYKKEVLDRLEWNLVLWFKDTYHVSVEIASIIITLLFARLYPQYTEDNMVHLVSEIHDIISFDHRSRREQYPGT
MQOV_U3    MILQIQLSIHVDVPAGKYDARYARRLAFYLVNRVAQENNIPRDIAGIAVSFLMSQVSLIHTSTDFDYLCGSIDVNLDIPSNARAQVPCLR
           *         . :    :       :    :       .:  ::..:  .  :: :     .       . :   . :  . :

LJAV_U2    DWRIDNFNIKLKETNVPLHLHLGIRTPMMPSATMISEVWGKRGRNPMPLKKEWSLAFKAEVYRWDYIFNVLQDPIILN
MOSV_U3    TWILESPLFSLAGKIIKVCFAGRITYPGGSGGQTPWETWYTSVRSKIPKELRREIEDAAHSYNFEYLLEP--------
KAMV_U3    TWILENPLFSLAGKIIKVCFAGRITYPGGPGGQTPWETWYTSVRSKIPRELRREIEDAAHSYNFEYLLEY--------
HPV_U3     KWISESPSFVLGDHVCKVSICGRVSFPGRPGGQTPWEIWYSTIRSKIPKEMRREIEDAAHSYNFEYLLDY--------
FLAV_U3    KWISENPSFVLGDYVCKVSICGRVSFPGRPGGQTPWEIWYSSTRSKIPKEMRREIEDAAHSYHFEYLLDY--------
MANV_U3    ALIGEKAMFKLDFYWCTINMGGFVTYPSPISGKKIWELWYGDHRRHIKPALRREIEDASEKYNYVYLIEYW-------
MQOV_U3    ELITINSPVFIGDETIHPTLWGSISYPALGTGVRPWEAWYTDRRCFIPSNLRLEIEDLAYDFKFEYILD---------
                .  . :        :   :  *    .    * *    *  :    . .:   :  :.: *:::
```

T

```
LJAV_U1    MDYFLHGGISIHKVPGSVTKEDIHYIMMKVINDLIHDLSMPHDLAGAAIALILSNVGYRDMSDGSVEGEGYIQEGVSFTKPSQHPELCNR
MQOV_U3    MILQIQLSIHVDVPAGKYDARYARRLAFYLVNRVAQENNIPRDIAGIAVSFLMSQVSLIHTSTDFDYLCGSIDVNLDIPSNARAQVPCLR
           *   :: .* :.  .*.   .  : : : ::* : :: .:*:*:** *:::::*:*.  . * .     * *: .:.:.. ::    * *

LJAV_U1    NWEHYGH-HYINREDGS-FFEYFIFMSRPLIFIGKPYIELWSCITFPHNYHKMTLSPDLLALEYDFAHMICHM-
MQOV_U3    ELITINSPVFI-GDETIHPTLWGSISYPALGTGVRPW-EAWYTDRRCFIPSNLRLEIEDLAYDFKFEYILD---
           :    .   :* .::      :  :   .*    :*: * *          :: *. : ** ::.* :::
```

U

```
LJAV_U1    MDYFLHGGISIHKVPGSV-TKEDIHYIMMKVINDLIHDLSMPHDLAGAAIALILSNVGYRDMSDGSVEGEGYIQEGVSFTKPSQHPELCN
HPV_U2     MNLQIHGYLSFI-LPRSIFTRRNLWCIQKGTVNELRRIAGITQDVSGLVCSMLFSRLDFDLTEDDKILSEVNLIENYMFPQRFNNVINLH
           *:  :** :*:  :* *: *:.::  *    :*:* :  .:.:*::* . ::::*.:.:.  .*..: .*  : *.  *.:  ::    :

LJAV_U1    RNWEHYGHHYINREDGSF-FEYFIFMSRPLIFIGKPYIELWSCITFPHNYHKMTLSPDLLALEYDFAHMICHM-------
HPV_U2     INVPS-LKYTMVVEGKAVGIHLILRIDTINQIGRTLYTAMWGKRRM-KN--SMGRTLEEDGKRFGFQYLFEMVYIAPPLN
            *     :  :  *. :. :. :: :.    :  . *  :*.    : :*  .*  : :  . .:.* :::  :
```

V

```
LJAV_U1    MDYFLHGGISIHKVPGSVTKEDIHYIMMKVINDLIHDLSMPHDLAGAAIALILSNVGYRDMSDGSV-EGEGYIQEGVSFTKPSQHPELCN
HPV_U3     MNCTFNLSTFFYLPEYLYKKSVIDRLEWEIISWMRDNYNISTELCALTTTFLMSQV-YPLTKDQDVLYLCGELRANVSFFKRSKHIQHPG
           *:  :: .  ::      .*. *. :  ::*. : .: .:. :*.. : ::::*:* *   .* .*:   * :: .*** * *:* :  .

LJAV_U1    RNWEHYGHHYINREDGSFFEYFIFMSRPLIFIGKPYIELW-SCITFPHNYHKMTLSPDLLALEYDFAHMICHM
HPV_U3     IKWISESPSFVLGDHVCKVSICGRVSFPGRPGGQTPWEIWYSTIR-SKIPKEMRREIEDAAHSYNFEYLLDY-
            :*   .  ::  :. . ..    :* *    *:.  *:* * *  .:  ::*  . :  * .*:* ::: :
```

W

```
PCV_U3     M-----------------------ATTNKNRRLTRRVYFRLDWYGDNIRDVDVIKLFKLTIESEDLLEIVKDMMIEDLIKTCRKS--FYD
LJAV_U2    MNLDVNCVIRFELPKQLFTQSNLLCIEGAYVRYAQRN-YRLNHDLSAIVINTAFSWLDMWPKNDELVQCYAVLFTDFLIPRRLRGSRDYE
           *                      :.  .   * ::*  :**:   .  *    .:. :.:  :.::*::    :: : **    :.   *:

PCV_U3     FVWPTHHRLVGGSAFFGPCPEHL--IDLLSDNISIKLDETGMFHKSPFTLIMEIDYNLDEDANECESQSPNNNRPSHLQF
LJAV_U2    IDWRIDNFNIKLKETNVPLHLHLGIRTPMMPSATMISEVWGKRGRNPMPLKKEWSLAFKAEVYRWDYIFNVLQDPIILN-
           : *  .:  :  .    *   **     :  . ::  :  *   :.*:.*  * .  :. :. . :      : *  *:
```

X

```
LJV_U1     MGSNIYLKS---------------------MVFVIGPKPLTFDLLKFIIRKIPKISQDYQVNCLGKLAIGLAYQRSEFDVS---KNSIQD
WONV_U1    MEYQFLKKSSFGPQLDTVYVRDNHMLFEGNLALIWEDEISERDLLLMLKEEISKFPNYQKYSSIYKIGVGILLSKSKYDFVWPDKSYLIS
ORV_U1     MEYQFLKKISRGPQLDTVYVRDNHMLFEGNLAFIWEDDLSERELLLLLKSEISKLPNYQKYSSIYKVGIGLLLSKSKYDFVWPDKNYLIS
PCV_U1     MEYQFLKKTSRGPQLDTVYVRDNHMLFEGNLVLIWEDDLTEREVLLLLKNEIVKFPNFKKYSNIYKLGVGLLLSKSKYDFVWPDKNYLIS
           *  ::  *                      :.::   .    ::* ::  :* *:.:  : . : *:.:*:  .:*::*.    *. : .

LJV_U1     GMFEGIIYFPWRSFPNLNSLDYSYKLRLTN-YCD-IYHIHIKIKASKSTER-GFTIWETWDRCQNNFLPDP--SLEYSSDHLGFSDLIH-
WONV_U1    GITD-IINFPNIQRCPWDPQEDRIKIDTCGIWQGKRYNLSLNLYFSQADPRLGRPIWESWYSSFNSRPPFMRFEIETVSDYLGFGELVHG
ORV_U1     GTSD-IVNFPNIQYCPWDPQEDRVRVDKCGLWQEKRYNLSLNLYFSYADPRLGRTIWESWYSELNSRPPFMRFEIETVSDYLGFGELVHH
PCV_U1     GMTD-IVNFPNVQFCPWDPQEDRVKVDTCGTWQDKRFNLSLNLYFTYADPSLGRTIWESWYSELNGRPINMRFELETVSDYIGFGELVHM
           *  : *: **  .    :. :   ::   . :    ::: :::  : :    * .***:*    *.       .:*  **::**.:*:*
```

Y

```
LJV_U2     M-----------------DFNLDQEEKSHPLFFNVEIHIGYRGPYFDSNDLLTQIDKMIESRPSSSNHTDLIRLAIGLGLAHCVHSQITP
WONV_U2    MATDENFDAKLAWVVESLDFSPSPRDDPVNFVISTKIDVDFP-SSFDEVELLMHIRQEMKKNKEWTKSGSFMGLCAGIALSHSMLVPTEG
ORV_U2     MATSENFDSKLAWVVESLEFHPNPRDDPVNFVLSVKIDVDFP-SDMDEINLLIHIRQELKKNKMWTQRGTFMGLCAGIGLSHSMFVPSDP
PCV_U2     MATNENFDSKLAWVVESLEFHPNPRDDPVNFVISARIDVEFP-SNFDEVELLMHILQNLKRNKMWPQKGSFLGICAGLSLSHSSFVPSDP
           *                 :*  . .:..  :.:...*.: :  . :*. :** :* : :: .   .:   :: :. *:.*:*.

LJV_U2     TLWNLNGSFMGVLKLPDYPVNPTVHTVVGQEITDGCGLLYPDISVYSKIYIMLGNESKVQDIITHWYTGEGGVCYEYDFSLPTVAGALKF
WONV_U2    LRKRLVGDFMGVLNIPLVPDQGTDYIILNTTSYNLDLNMWSEIKLSYTFFVCRGNGNVTKRIDTTWYANQPDRPEYLTFDLLTVSVLYGF
ORV_U2     LRRRLMGEFNGVLNIPLVPSVGDDYIILNTTSYNLDLESWSNIKLSYTFFICRGNGKVTKRIDTSWYSGQPARKEEYTFDLLTISVLYGF
PCV_U2     LKKRLIGDFMGVVNIPLVPSVGNDYIILNTTSYNLDLESWSEIKLTYNFFICRGNGNVTKRIDTAWYGGQPKRGEDFTFDLLTVAVLYGF
              .* *.* **:::*  *     : ::.    :     :.:*.:  .:::  ** . .: * * ** .:        *.* *::    *

LJV_U2     EHLFL----------
WONV_U2    DDWFVSPLVNYND--
ORV_U2     DDWFVSPLVNYVEDP
PCV_U2     DDWFASPLIDRDD--
```

```
          :. *
```

Z

```
LJV_U3    MSTQKAYAFVSGHFEYKSFHFDKTEFCKALIDLIKTFTQDPVKVQWICLLSTIAALKGKILFKGDNTWGVRIYLECNLDLKGKWNLPRVE
WONV_U3   MATSD-NPRLTRRVYFRLDWYGDNLKDVDVVKLFKLTIESEEILDPIKEMIIEDLIKTCRKSFYDFVWPTHHRLVGGSAFFGPCPSELVD
ORV_U3    MATTNLNKRLTRRVYFRLDWYGDNLKDVDVIKLFKLTIESEDVLELVKDMMVEDLIRTCRKSFYDFVWPTHHRLVGGSAFFGPCPENLIE
PCV_U3    MATTNKNRRLTRRVYFRLDWYGDNIRDVDVIKLFKLTIESEDLLEIVKDMMIEDLIKTCRKSFYDFVWPTHHRLVGGSAFFGPCPEHLID
          *:* .    :: :. ::   :...     ::.*:*   :.   :: :  :     ::       * .* .:  *  .  : *      ::

LJV_U3    VIKEDYCSTGSPFMSQVCGWLAVNIKNGSRPFLRKKHYSPDYREDHHSDISLGEFLRQAGLGGMIP----
WONV_U3   LLTDD-----------------VSIKLDETGMYNKSPFTLVMEVDYNLDEEANNCESQNPNNNRPSHLQF
ORV_U3    LLKDD-----------------ISIKLDETGMFRKSPFTLTMEIDYNLDEDTKECESQSPNNSRPPHLQF
PCV_U3    LLSDN-----------------ISIKLDETGMFHKSPFTLIMEIDYNLDEDANECESQSPNNNRPSHLQF
          ::.::                 :.** ..  : .*. ::   . *:: * .  :   *   ..  .
```
