# Supplementary material for: Evolution of Genome Size and Complexity in the Rhabdoviridae
Source: PLoS Pathog. 2015 Feb 13;11(2):e1004664. doi: 10.1371/journal.ppat.1004664 (PMC4334499; doi:10.1371/journal.ppat.1004664)
Supplement: S4 Fig — (PDF) [file ppat.1004664.s004.pdf]

Figure S4. Amino acid sequence alignments (Clustal X) of GLOV and MANV U1x proteins encoded in ORFs overlapping the U1 ORF in the first transcriptional unit located in the region between the P and M genes. The alignments illustrate homologies between the U1x proteins and with selected other hapavirus PMIPs, providing evidence that they have also arisen by gene duplication. Identical amino acids are shaded.

A

```
MANV_U1x   MFILLSTDLIISCMIKMDFYYINLYLEFSLPKSAYCYAALIHIEKRLQDIISKKYKI---LSGIISGWAISNLEVVPGNYGLVECQSIIQ
MANV_U1    ----------------METF-LHGGITLFCNSILVPKTLLHEIILKITNDLVHDCGMPPDLASAISTLLLSNTLFKYLSDGTVEGTGFIQ
MANV_U2    ----------------MDLN-VGCYIDFTFPNNLFDLKTLELIEGACIRWCQRHINLNKDLIAIIINLAFSQVEFRPAQNQKVYGRSEVN
MANV_U3    ----------------MNIS-AGVSLSFNLPKELYKKEVLDRLEWNLVLWFKDTYHVSVEIASIIITLLFARLYPQYTEDNMVHLVSEIH
                           *:       : :   .       *  :             :   : . *    ::.      .   *   . ::

MANV_U1x   DAIQIKFDPTSVCN--DLVVNKRFELKVLQWVIPIKLLLEVNRSHFPIGSRLTSLWGRRGDSFMIHDEMWDIKTVANRLGFADAI-----
MANV_U1    DGVSYKGETKWIDQKGKNWNKTGIHDLTDVSGSFFEYFIFCSEPAIFEGKPFEELWATQDSHHPYFKQKMNLDVYSFEYGFNHLLHD---
MANV_U2    NYLELQNFLRGSKDYFIDWAKHDFTMKLTSAEIPIHINFYILPYRGGLLRTLDQAWGRNYKSTMLRIPEWQLEYKAHLYNYSYMLTRSTE
MANV_U3    DIISFDHRSRREQYPGTALIGEKAMFKLDFYWCTINMGGFVTYPSPISGKKIWELWYGDHRRHIKPALRREIEDASEKYNYVYLIEYW--
           : :. .                            :.               : . *              ::.  :   .:   :

MANV_U1x   -----
MANV_U1    -----
MANV_U2    LPELN
MANV_U3    -----
```

B

```
MANV_U1x  MFILLSTDLIISCMIKMDFYYINLYLEFSLPKSAYCYAALIHIEKRLQDIISKKYKI---LSGIISGWAISNLEVVPGNYGLVECQSIIQ
LJAV_U2   M------NLDVNCVIR-----------FELPKQLFTQSNLLCIEGAYVRYAQRNYRLNHDLSAIVINTAFSWLDMWPKNDELVQCYAVLF
          *      :* :.*:*:           *.***. :  : *: **       .::*::   **.*: . *:* *:: * *  **:* :::

MANV_U1x  DAIQIKFDPTSVCNDLVVNK--RFELKVLQWVIPIKLLLEVNRSHFPIGSRLTSLWGRRGDSFMIHDEMWDIKTVANRLGFADAI-----
LJAV_U2   TDFLIPRRLRGSRDYEIDWRIDNFNIKLKETNVPLHLHLGIRTPMMPSATMISEVWGKRGRNPMPLKKEWSLAFKAEVYRW-DYIFNVLQ
            : *     .  :  :  :  .*::*: :  :*::* * :. . :* .: ::.:**:** . *  .: *.:   *:   : * *

MANV_U1x  ------
LJAV_U2   DPIILN
```

C

```
GLOV_U1x  -------------MKTRMYGFLRIDLDPLIVYHRNAWELIFGGINEFLSSTRLLWQKEQALLTGLLVSALEFD--HYSAEKCSAFAHIVT
MANV_U1x  MFILLSTDLIISCMIKMDFYYINLYLEFSLPKSAYCYAALIHIEKRLQDIISKKYKILSGIISGWAISNLEVVPGNYGLVECQSIIQDAI
                       * .  : ::.: *:  :     .:  ::   :.: .     ::  ..:::*  :* **.   :*.  :*.:: : .

GLOV_U1x  LLDSDSTNPGSIPNHYVSDSFHVEILGKTARVMIQLDIRMEEG-VGETLWTIHSRISNQQPFTALRIPLMKLIK-LTLSS--
MANV_U1x  QIKFDPTS--VCNDLVVNKRFELKVLQWVIPIKLLLEVNRSHFPIGSRLTSLWGRRGDSFMIHDEMWDIKTVANRLGFADAI
           :. *.*.     :  *.. *.:::*     : : *::. ..  :*. * :: .* .:.  :    : .: : * ::.
```

D

```
GLOV_U1x  MKTRMYGFLRIDLDPLIVYHRNAWELIFGGINEFLSSTRLLWQKEQALLTGLLVSALEFDHYSAEKCSAFAHIVT--LLDSDSTNPGSIP
HPV_U2    MNLQIHGYLSFILPRSIFTRRNLWCIQKGTVNE-LRRIAGITQDVSGLVCSMLFSRLDFDLTEDDKILSEVNLIENYMFPQRFNNVINLH
          *: :::*:: : *   *. :** * :  * :** *     : *: ..*: .:*.* *:**  . :*  :  :::   :: .  .*  .:

GLOV_U1x  NHYVSDSFHVEILGKTARVMIQLDIRMEEGVGETLWT---------IHSRISNQQPFTALRIPLMKLIKLTLSS--
HPV_U2    INVPSLKYTMVVEGKAVGIHLILRIDTINQIGRTLYTAMWGKRRMKNSMGRTLEEDGKRFGFQYLFEMVYIAPPLN
           :  * .: : : **:. : : * *   :  *.**:*              : ::  . : :  :  :    ..
```
