# Supplementary material for: Evolution of Genome Size and Complexity in the Rhabdoviridae
Source: PLoS Pathog. 2015 Feb 13;11(2):e1004664. doi: 10.1371/journal.ppat.1004664 (PMC4334499; doi:10.1371/journal.ppat.1004664)

Figure S6. Small transmembrane proteins encoded in the genomes of the sripuviruses (SRIV, NIAV, SMV and CHOV Gx proteins) and the curioviruses (CURV and IRIRV U2 proteins and RBUV U3 protein). Homologous sequences are aligned with transmembrane (TM) domains indicated. A schematic illustration depicts the predicted membrane topologies of sripuvirus Gx proteins (A), CURV and IRIRV U2 proteins (B) and RBUV U3 protein (C). Amino acid sequence alignments were conducted using Clustal X.

Sripuvirus

TM domain

```
SRIV_Gx     MESFSLFVELFRLLLIMYFTTPLKRIFIGIQLIIVVCGVLYGVLGYLTHLQVESLYNFPAIQATMIFLDTVVTKLSGFQSVL
NIAV_Gx     MDYFFLLVEFSRLLLVMFFTTPLKKIFIGIQLIIVVFGALYAVLASLTPLQVGSLFQYPQIQATMIFLDSVVTKLNGFQSVL
SMV_Gx      MDLSCWLLELFKLLMAVFFISPVKRIFIGTQLTILVLGALLGLATSILGTVQMSSSLSHIVTLSISLMDTAVTKLSGFRSVL
CHOV_Gx     MDLSCWLSELFKLLMAIFFISPLKRIFIGTQLIILVFGALLGLAINIPDTVQMSSSVYHIVTQAISLMDTAVTRLSGFQSVL
            *:    : *: :**: ::* :*:*:**** ** *:* *.* .:   :      *      :  :: ::*:.**:*.**:***

SRIV_Gx     KHGIGALMLRNTSGQSQ
NIAV_Gx     KRGIGALMSSSTSDQ--
SMV_Gx      NRGIGQLILNSISESCQ
CHOV_Gx     RRGIGQLMLNNISGSCQ
            .:*** *:  . * .
```

Curiovirus

TM domain

```
CURV_U2    MKNTMEAKAVVAGILLLSSLILFPTL---DAQALTILKRTCGDGMMKRVPCKLMDSVENGISARGSCLIFCSSKKKIKGKDG
IRIRV_U2   MKKTMGAKGRLACIFIG--MVFFSYLRESDQQGFTYIKRTCGDKMMRVIPCNLMDSVKTLTGPKGKCLVFCSSKRKIKGRDG
           ** ** **. :* *::   :::*. *   * *.:* :****** **: :**:*****:.  ..:*.**:*****:****:**

CURV_U2    RMRNLCLTGDPRADEIIKCREQILKKKTEI
IRIRV_U2   K-QDLCLTGDPRSDEVMKCKDKISGTKSP-
           : ::********:**::**:::*  .*:
```

TM domain

```
RBUV_U3    MSSIDSGVMTTRSKRSSGRFMGPINSKLALSVFVLILVRITVTRGYQLATKGCDEYNYSYIVDCVEGSYFGLCKIKTLGDVC

RBUV_U3    HWYGLDNWKAEVCHVYLNRYHDDEFANLSLDSDRGCEEVQEYLARGQSSNIL
```

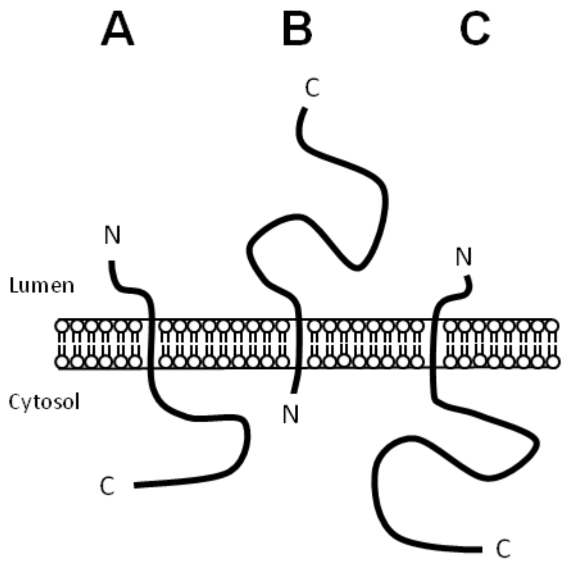

Supplement: S6 Fig — (PDF) [file ppat.1004664.s006.pdf]
