# Supplementary material for: Evolution of Genome Size and Complexity in the Rhabdoviridae
Source: PLoS Pathog. 2015 Feb 13;11(2):e1004664. doi: 10.1371/journal.ppat.1004664 (PMC4334499; doi:10.1371/journal.ppat.1004664)
Supplement: S7 Fig — (PDF) [file ppat.1004664.s007.pdf]

Figure S7. Small hydrophobic proteins encoded in the genomes of A) tupaviruses (DURV, KLAV, TUPV U1 proteins) and unassigned viruses (GARV, SUNV, KWAV and OVRV U1 proteins), and B) sripuviruses (CHOV, SMV, NIAV and SRIV Mx proteins). Sequence alignments (Clustal X) in which conserved amino acids are shaded illustrate that the proteins all have a relatively high proportion of hydrophobic residues (L, I, V, M, F, Y, W) but fall into three homologous groups.

A

```
DURV_U1    MYVELLVLGLFF-LCGQRIFLFGLVYLLGRFNLLWFVFDAIMFCLWVFCKNLPSEVLRLT
KLAV_U1    MLGEILIAGICY-LLGRRFFWGYLAYIAGQYNLLHYPLMIIQFLLWLLFYNAPYQLWGLI
TUPV_U1    MITTLIIIGAAF-LVGPRTFKFVLAYLLGYYNAFGPPLQIVQFMVWLIIIYFPKKFFSLG
SUNV_U1    MI-LLVVLILFFGMLYKRLSFLMMAYLLGYYNVFGSVITYGSFFIWYLFYYLPSKWMGAG
GARV_U1    MIALFLLLTLLVMVLRPRYVEWILFYMLGHYNVGLNALYNVNFLFWYLFCDIPSRFLN--
           *   :::      :   *     : *: *  :*     :    * .* :    * .

DURV_U1    GDWLKPSWESFQEE-----------MYSS-----
KLAV_U1    WSTFQSSFDEFNNPGEAAISPLDLPIYRRLGQK-
TUPV_U1    WYFCHDAFSSYFGDPNGGQLPVSTKFHSLTDMID
SUNV_U1    FAAIVESYNKEYAE------------WVSIE---
GARV_U1    -NVFGDMIEKYYQD--------------------
                   ..
```

```
KWAV_U1    MAGWKLLFVLLIVLYWHNPEGVTSLMKSSLNIMETILAEPIRKVVSFFTPP--CPPCPQC
OVRV_U1    M-FWKVFFALVLFSYWNNPDVAS---RTATTIFDMMLLA-TRYIASYILPASACPPCPEV
           *  **::*.*::. **:**: .:   ::: .*:: :*    * :.*:: *.  *****:

KWAV_U1    LVKTP
OVRV_U1    HAPFP
            .  *
```

B

```
CHOV_Mx    MIILPIEMFAPFIVIAFKRIRKIIIKFLLAT-CANLNVQG--EIKERFYLYVTGMWEIYR
SMV_Mx     MIVFPFEMFVPAIVIFLKKFRLITIKLLLAT-CADFNLRG--DIKERFYDWVMGVWGVYK
NIAV_Mx    MIVLSVDYFYIPILWILKLLRNWALRLCIGSYCVDSGIEGASPVTSQVISKCQQILRQAR
SRIV_Mx    MNLISMESLYIPVTLFLRTIRRWMLKLLYSSFVVDARIDSYGQFYSQLICKVQKIIEQMR
           * ::..: :   :   :: :*   :::  .:  .:  : .   . .:.      :    :

CHOV_Mx    HGRKQDFQALLSEELREIRYDP
SMV_Mx     NGRRSEYLDLLSEQLIEVVIEP
NIAV_Mx    DRNLERWRELANEALLDLFPD-
SRIV_Mx    CRERESWRELSDKAMNEIIE--
             . . :  * .: : ::
```
