# Supplementary material for: Evolution of Genome Size and Complexity in the Rhabdoviridae
Source: PLoS Pathog. 2015 Feb 13;11(2):e1004664. doi: 10.1371/journal.ppat.1004664 (PMC4334499; doi:10.1371/journal.ppat.1004664)
Supplement: S8 Fig — (PDF) [file ppat.1004664.s008.pdf]

Figure S8. Sequence of the predicted class I transmembrane glycoprotein encoded in the MCOV genome. The predicted signal peptide and transmembrane (TM) domains are shaded in grey and two predicted N-glycosylation sites are indicated in bold and underlined. The protein contains only a single cysteine residue (shaded black) in the predicted ectodomain.

Signal peptide

```
MYLKTLVLLCGIGVIQSVTQDDRRSKINLLPFLKLEWLDDLRKPVQDVVMDSQAVFKEIFPNILREMQAIFQKID
REISTKLFDPSREAFQKMIEGTDRIIKQTDELFTKKIPEEFNKMSREIARSTQESINMYKRFEKDFKDKIIEPGS
HAIGSVRQEITSSVLMLKNNMSTSISRMTWNIGNEIKSLKMGLVNEKILSLGDQINEKVIKKTDQVLTIFKDTLN
NKVSPLFQKLSQIDFKGSVDQVNQLIAATKGNVVTPVTVLIQSVKQLIENYSKSINNYLMPTATKILDQVSDGTK
TVFDLMSTSSKKVHDCILEIQASLSTKVIPVFEGFANYVTAISVALKPVLENWKDSIEQAFYIIGYNINQVINQF
NIYFKDINWNSWIQNTKTWITETYLSGKAALFDFIKSASEYSFKAIKVIKIIAIVLTIITLGIVVIWIINKVMII
SRWCCPNNS
```

TM domain
