# Supplementary material for: Evolution of Genome Size and Complexity in the Rhabdoviridae
Source: PLoS Pathog. 2015 Feb 13;11(2):e1004664. doi: 10.1371/journal.ppat.1004664 (PMC4334499; doi:10.1371/journal.ppat.1004664)
Supplement: S9 Fig — (PDF) [file ppat.1004664.s009.pdf]

Figure S9. Amino acid sequence alignments (Clustal X) of small accessory proteins encoded in the genomes of ephemeroviruses. A) α2 proteins. B) β proteins. C) γ proteins. D) δ proteins. Identical amino acids are shaded in a hierarchy of light to dark shading from the top sequence (BEFV) downwards to highlight the relationships between the viruses. The poor alignment of the YATV U2 protein with the ephemerovirus α2 proteins is shown.

A

```
BEFV_α2    MFG--YMEISVRVEIGKQNSR-----IHKLELWKLMEEGLHTLMKEEKLDIMLKEEANFGFCRWLNTRGNWLYLEDMRKPILIEFQNFFN
BRMV_α2    MFG--YIEIKIILDEKIKKDV-----VHKLELWRLTEEGLFDLMAKGNLNSVLKEEANFGFCRWLNTKGNWLNKSEMRKPIIIEFQNLFD
KIMV_α2    MAA---------------EEL-----LTNLSLSRSWDKGLIDEMKVA-LSRKINQIPNW-ICSEDNIKVDIFYVGHMKGKAKIDYFEL--
ARV_α2     MPF-------------------WV--EVRINNLDEGDISALENNFTQNFNNLIDQNKEIIKYIIQKVKTQSYLEGKLHP---FYYINLID
OBOV_α2    MSS-------------------WV--DVRLKGVKKSDIDWFIFRMRESKSKSEIKNREIIIYCLDQIKKEYSQTEDLPA---FYYIKLKT
KOTV_α2    MSS-------------------------KFEL-DIINKTKYNITCESLIQLLFENYKRLGFNTYDKLLLQCLKTIGEENKGLIMYRGFSS
KOOLV_α2   MSS------------------- -----KFEL-DIINKTKYNISCESLIQILIENYKKIGFNTYDKMLLHCLKLIGNDNKGLLMYKGFSS
YATV_U2    MDIIGTGSILLVYNGNVPIDYGWFLFKLQQELYTYDQGYTLYCILRSILNNHYTVYGDGIEKGYRDLTSTIPYTEELMRSGRGGTHTWED
           *                           . .     :            .               .

BEFV_α2    CLNYPSRVYKLTVQNNDYKLGSIRDLRIKLFFF--
BRMV_α2    CPSSKAKVYKTKIANREYKLGSIKEMTIKLFFF--
KIMV_α2    -----KKSIRPKLHN----LGSLDMLEICLFWTQI
ARV_α2     TNDLHKPCFLTKFGLKSRFTREEKGYIVIEGHYH-
OBOV_α2    PNSLCKSCSLIKISKLSK-SDNLTRYVLFEIHHHI
KOTV_α2    FHGKMKSVILIPIEG-SVKLDINNDILTILYRYNS
KOOLV_α2   FHGHMKNVILIPIEG-SVKLEIDNDTLTILYRYNS
YATV_U2    IIGIPLNDGKVYLYAKLTWTIPKKKPWYKKIFQ--
                       .
```

B

```
BEFV_β     MDFIRCHVAMQIINFKA-LEIDKRSLLGILVIKNIKNLH-------RSNQLLTRLSDLMVPSVIHNGEFVMRNDKSDKLWIFVGESWASL
BRMV_β     MDFIRCHVAIQIINFRA-LEIDKRSLLEILVIKSIKNLH-------RSNQVLTRLSDLMIPSAIHNGEFVMRNDKSNKLWIFVGESWASL
KIMV_β     MEFSRCKLSLQIINFKP-IEIERRALLGLLAVKSIKTLH-------RGNQMITRLSEILIPSAIVNGEFVVRNDKKNGYWIFVGEAYGAI
ARV_β      MEVIRVKINLCFYAESK-LDIMPNHVLGVIIIDNIRSIY-------GEEGLIAYCMERLVPLCMYKGERTVYN---HGRWKLVTHLSYLI
OBOV_β     MEVIKVKVDLCFYAESN-LDIMPNHVLGLILIDNIRTMY-------GEEGLITYCMERLVPLCMYKGEKKIYN---HGRWKMVTHFSYLI
KOTV_β     MRSLVYGVDIRLMIYGIGREMLNFAILHKIIRQNIAWTYGIDMRGDNEKEEKEKALNFLLKCWDQLGPSIYINNQKLLQDKIIGQEKLTT
KOOLV_β    MRSSIYGVDIRVMIYGIGKELVNFAILHKIIRQNIAWTYGTDFGSDGDKKDRMRSLDFFLKCWDQLGPSIYINNQMLLQSKITGQEKLTT
YATV_β     MSGKLYSIHMEVNIYPNDEPFNSYNQIYELCIQALERSR-------APIGLIKVFKQFGFTALLCSDIKTELNDLGVTKYKGVVDGYGEF
           *      : : .        :     :  :  . :                     :  .      .     *           .

BEFV_β     DL-EDLNGVR--DNVFNISKTVPLLIQGEEYGVIDLSIKVEPRGLRFLKRSSEIDIC----DIPKKVRVVPT-
BRMV_β     DF-EDLSGVR--ERISSINKTVPLLIQGEEYGAIDISMNIEVRGIRFLKRPNEMDIC----DIPRKLRVISA-
KIMV_β     DS-DDLRGVR--EKVMKISRTIPLIITGEEYGAIDLDITLEDSEIIFLKRKNELDIC----DLPRKMRFLH--
ARV_β      DRPTNPMLLR--IKEREIEEVMSVHIMDENVGQLYFKLELQQSDFNFLKHKLDPAIK-AEGEPPKKVTLI---
OBOV_β     DKPANPMLLR--IKEREIEEVLSIYILEENVGQLYFKLEIQQSDLNFLKHKLESSSKSADGEPPRKVTII---
KOTV_β     RGIIDVEYTRPIEEYYFYNRIFPIENSGGLLCHVGVEIEGILLQFDCPKRSRERDDC----PIPFKVRIIE--
KOOLV_β    RGIIDIEFSRPVEDYYFYNRIFPVENSAGLLCHIGIEIEGVLIQFDCPKRPRGKDDC----PIPFKIRIIE--
YATV_β     YLTTYEEGVI-----EDVSTLLYLQEGGKLRAHAWILLRAHHVKAGSFKR-KLLDSC----PIPPKVIVLEGP
                             .  . :           . :          *:             * *: .:
```

C

```
BEFV_γ     MDLKFRCLIKNVADGRAGEIIVEECLEIIEQKYLRLMTIDLKEIRSSMFDQES-NPWVYVFGKIYISGLMGRAIGKRMVKRGTYRIKEGE
BRMV_γ     MDLKFRCLIKNVADGRAGEIIVQECLDVIENKYLRLVSFDFKEIRSSIFDQES-NPWVYVFGKIYASGMMGRIIGSRMVKRGTYRIKEGE
KIMV_γ     MELKFRCLLRNVNDCAAGERIVFEVLELIERKFMRLIAIDILDIRSSSFDRESHNPWVYVFGQIQVTGILGRVIGRRMIGRGSYKLKSHE
KOTV_γ     MISKGEIMIVNHDYTPLSKLELIFELEKILDLSERHR--PIIEVVQKLIKNDWINLGVHRF--TYKIPLKG-----MYQIKGCWEIKMN-
KOOLV_γ    MSSKGEIMLVNHDYNPISKLEVIYELEKIFELSERHR--PIIETLQKVIKADWINPGVHRY--MYKLSLKG-----VYHIKGSWEIKSN-
YATV_γ     MALLLSGVLT--KSDFQTDNQLFDFVEKIVEETTRLAIKNSIWWSGLQFTQIEREVMVGMNWLVYIDNEESDRVDYSFWFGVPDSVGWRM
           *      ::         .  :   :: *     *             :     :  *            .              :

BEFV_γ     LINHFEGVHIHFYKDIEKIFHSIRV---
BRMV_γ     LINHFETVHIHFYKDLGKIFNHKYS---
KIMV_γ     LQGHFEDVYIHFYEDIKRMFRSYY----
KOTV_γ     LNIPINGRIITLDTDIINQM--------
KOOLV_γ    INIPISGKIITLVLDIINQI--------
YATV_γ     FRNPNTRCIASNILENRQSYCFIQYFSQ
           :             :  .
```

D

```
KOTV_δ     MALIKIEGEVGTNEVRTELVITVIKEVNSIIMDILQCIGIPFQPDKMLTRRDIKMELDPEDHAVCFIRIEKSWETECDRVGKLSFLENLG
KOOLV_δ    MALIMVEGEVGSSEASTGLVVTLVKEVNSLIMDILQCLDIPFEPGQMISRKDTTMEVDFEDRSVCFIKIKKEWEVESIKVGTLSFIEELR
           **** :*****:.*. * **:*::*****:*******:.***:*.:*::*:* .**:* **::****:*:*.**.*. :**.***:*:*

KOTV_δ     GRGNFSYSGNMKLEISPKL
KOOLV_δ    GKGYFSYNGSMKLDILPKP
           *:* ***.*.***:* **
```
