# Supplementary material for: Evolution of Genome Size and Complexity in the Rhabdoviridae
Source: PLoS Pathog. 2015 Feb 13;11(2):e1004664. doi: 10.1371/journal.ppat.1004664 (PMC4334499; doi:10.1371/journal.ppat.1004664)
Supplement: S10 Fig — (PDF) [file ppat.1004664.s010.pdf]

Figure S10. Amino acid sequence alignments (Clustal X) of: A) U1 proteins, B) U1x proteins and C) U3x and U4x proteins, of the curioviruses CURV, RBUV and IRIRV; and D) RBUV U2 protein with the ITAV U1 protein. Identical amino acids are shaded. Domains containing concentrations of acidic residues (D, E) in the U1 proteins and basic residues (K, R, H) in the U3x and U4x proteins of CURV, RBUV and IRIRV are shown in bold and underlined.

A

```
CURV_U1        MVPSKTLGERMLVDLLECHCLVEEEETENERLYICTGDPDAIGDLFARACVWLVNLEALGCFSPHWDLDHEEPGRLVISVFP
RBUV_U1        MVPSKTLGERILTELLESHALLEEEEFEAERVYICRGNPDQIGALYARACVWLVNLEAINLLSSNWDLDHEEPGKLVVTIFA
IRIRV_U1       MVPSKTLGEKILTDLLEAYCLIIEDEWEEERVYTCEGPPDKVGDLYARTCVWLVNLEALGLITKNWELDHEEPGRLVIKIYC
               *********::*.:***.:.*: *:* * **:* * * ** :* *:**:*********:. :: :*:*******:**:.::
```

B

```
IRIRV_U1x      MKSLADWLSKSTVDGPVFITKMFKEENGLIDFYLDLYLEVVPIG--LEVRFCRSVVEPLHVIFIISP-------
RBUV_U1x       MKSLGSWLSQSSHDVPIYIIKWFEKEEDVVDFYIDLFLNAQEVSKEIVVKFHRSVIDPLYVIFEISSGPLSQHP
CURV_U1x       MKSLGDWLFQYSLDRSLFVTRMFESEEEFLDFYVDLYIACKSVNESIHVTVTRSLIDPFHVIFRIWQT------
               ****..** : : * .::: : *:.*: .:***:**::    :.  : * . **:::*::*** *
```

C

```
CURV_U3x       MIKIRKEGSSKDDFLFYQKVGERIQNIFPDAINLKVHRSQDGVFILDLTWPPGVSVLLVPRRLKTVRRFVSERPGRELFLLG
RBUV_U4x       MIRLVKSKETMEDYLFYRNLGERLVDMFPELFFLSITRSDNGVVSLDLNWRKGTPILLIPRRLKTQRRFVSYRPGRDIYLVS
IRIRV_U3x      MIKIRKTQENRDEFSLYQRFGERLVSLFPDSFDFYIKREEGGTIALTLTWPPGLPVLIIPRRLKTSRRFIVYRPGRDLFVIA
               **:: *  .. ::: :*:..***: .:**: : : : *.:.*.. * *.*  * .:*::****** ***:  ****:::::.

CURV_U3x       DLLYNKMGLKRSQIDTSYEMIHGGKWAIISIYG
RBUV_U4x       DFLYGKVGLKKSQIDCTYSLIMKGKAAIISIHG
IRIRV_U3x      NLLYNVMGLKKSQIDMRYEMINEGKWAIVTLYG
               ::**. :***:****  *.:*  ** **::::*
```

D

```
RBUV_U2        MKVVFIIYTLLFSHLNSSEIFDDEESSCDGNE-LEK-LQCMLDSLNSG---GVLESVPQTSTSPQGVGAIISGIEKPSFFLQ
ITAV_U1        MD-----PPSYW--VNGARPVLLRESYPVGEQFIFRGFTSLLGSLNKANIVNMLEHLQKFASSPIYYKIEDSILEEGAFFVT
               *.      .  :  :*.:. .  .**   *:: : : : .:*.***..   .:** : : ::**       * :*: :**:

RBUV_U2        LFLPFAMAFRDSIV
ITAV_U1        ILDPQNPQHSSV--
               :: *    . .
```
