# Supplementary material for: Evolution of Genome Size and Complexity in the Rhabdoviridae
Source: PLoS Pathog. 2015 Feb 13;11(2):e1004664. doi: 10.1371/journal.ppat.1004664 (PMC4334499; doi:10.1371/journal.ppat.1004664)
Supplement: S11 Fig — (PDF) [file ppat.1004664.s011.pdf]

**Figure S11.** Amino acid sequence alignments (Clustal X) of A) the U1 proteins and B) the U2 proteins of the tibroviruses TIBV, BAV, SWBV, CPV and BASV. Identical amino acids are shaded to highlight the relationships between the viruses.

## A

```
TIBV_U1     MEQ------------TWMITCKVEFRSSVMFELDYEDTVLNLLQALPTNQTNP------AKFIINSLTIPLAVQNAFQNKDFWR---
BAV_U1      MEQ------------TWMTSCKIEFRSSVMFELDYEETVLNLLQALPTNQTNP------AKFIINSLTIPLAVQNAFQNKDFWR---
SWBV_U1     MDQ------------TWMVTVTVEVRSSCLFSLKLDDTILNLIQCLPTNVVNP------SKFIINSLIIPLAMRDCFNKMNCWQ---
CPV_U1      MAS------------TWAVSVTAAVSSVFLRELSCHETMLNIVKSLPVSQSDI------VHHYLNTVGIGPAIADCFRNIKLWE---
BASV_U1     MQHEKNYTKDQNPDHRKTSTGITPRRATMAFHPMLCRLELSVSAAPPASPIDATLLRSLITSVLNSSWVDLGGNTANEKVLCAIGLI
                               :      :            *.:  . *..  :           :*:  :  .    . .:

TIBV_U1     ---------DPTGSKGEFHLVIKVILDGLENNENDWADSITCHWLDQRSGFYVMVDSTFVGHKLS--GEIMCTVDEKICECIE-KFA
BAV_U1      ---------DPTGSKGESHLVIKVILDGLENNENDWADSITCHWLDQRSGFYVMVDLTFVGHKLS--GEIMCTVDEKICDCIE-KFA
SWBV_U1     ---------DPTGSKGSHTAVIKVRLDGLNNSEHDWAESLTCHWLDKSTGFYVMADVTFVGHKLE--CEVDCSMTETDCDMIS-KFA
CPV_U1      ---------SPHKSIGNRTVVFKLRSQIGQPSEKDWSDSITLHWLDKRTGTYTMVDLDFVVYCIN--DEIHGYITQYDLDNID-PYL
BASV_U1     EAFCRERVIPPTSNSFNTSVTYHIMVEDLDPDDLGNIQLINKPLLSLEGDLKVLGSYQLTFQTIPGHSEPR-SMTDNGIYHSDSPFF
                      *  .  .   . ::  :  : .: .  : :.   *.   .  .: .  .   :    *      :        : :

TIBV_U1     NIIP-----NKYNYYHHTNYRFHIQRYRVNISIG---------
BAV_U1      NIIP-----TKYNYYHHTNYQFHIQRYRVNISIG---------
SWBV_U1     SFIP-----INYNKFHHSKYTLIIEKYRVNIKAP---------
CPV_U1      VLFE-----EQGNRITKRKLDIGIFFGELHINEGTEV------
BASV_U1     QIALGHALLGTGKIYDHITRALRVAPITIAPEKRKEPLSSYMV
             :          :   : .  : :    :  .
```

## B

```
TIBV_U2     MAT---------QA-HLLVSYYYDISSEGVPGVPINTICHGLEFDYMGN--NTTDDLEKVFLGSILRADLRGS--GYYSYIRLVNEG
BAV_U2      MAT---------QT-HLLVSYYYDISSDGVPGVPINTICHGLEFDHMGN--NTTDDLEKVFLGSILRADLRGS--GYYSYIRLINEG
SWBV_U2     MPV---------HTTYVLLSYYYDIESEDVPGLPISTICHSLDYNREGT--SHHDDLEKVFLASAIKTDLKGS--SYFAYIRLVNEG
CPV_U2      MAV---------QKRYYSISYQYDISLQNLPGLPISTLVNSLDFNESNK--RLSNDQEKVLFSAALTNDLHGS--LNFGYDRLANRG
BASV_U2     MQSSKKQSTELTKMDKISPSYYQVTLKIQSSGSSNDCISRERFYHKIMRRFNPRHHALGIYLGGEIIMQMIEENISYIPSNKCWKVS
            *           :      **         .* . . : .   :.         ..   : :.. :  ::  .        :  : .

TIBV_U2     KICLKIAFDQSLNWTLSGEKEFKSDFRVGSGTISVTFKCYWLRVSEKIWKGSLYKFDTTKNPQVLVYRLLEPKKQR------------
BAV_U2      KICLKIAFDQSLNWTLSGEKEFKSEFRVGSGTISVTFKCYWLRVSEKIWKGSLYKFDTTKNPQVLVYRLLEPKRTK-VRRTFYASKA-
SWBV_U2     KICLKLNYDNTVNWIKGGDNSFTTEFNVGQGSVRLKAKFYWLNVSEKIWKSSIYKLDVTRNPDMMVYKNIIPKRASGYRRAFYASKAA
CPV_U2      VIVVKIMVDNSIQWMDVGDYVGSAYCNVGPGSAKFEIQLNWVRMSQKVWKSSVARVDLTKVPELQIYRHITVKKCKRGKKFFMMSVD-
BCASV_U2    GLFSLRLKNLDLLQTTGRTSFTIIDKHIFSADLIISGYIEYQKVMRHEWELGAKKFVIN---SISGHPNLKVREDSLGNKYLKYKDI-
             :      :  :              .:  .   .     : .: .: *: .  :.  .   .:  :  :  :.
```
