# Supplementary material for: Evolution of Genome Size and Complexity in the Rhabdoviridae
Source: PLoS Pathog. 2015 Feb 13;11(2):e1004664. doi: 10.1371/journal.ppat.1004664 (PMC4334499; doi:10.1371/journal.ppat.1004664)
Supplement: S12 Fig — (PDF) [file ppat.1004664.s012.pdf]

Figure S12. Analysis of the potential ribosomal frame-shift (RFS) sites in the sequence overlap regions in the G gene transcriptional units of curioviruses (MSV, BGV, HARV) and some hapaviruses (WONV, ORV, PCV). A and B. Overlap region in A) MSV and B) WONV showing nucleotide and translated amino acid sequences in two reading frames. The potential RFS sites are double underlined and the TTP sequences are single underlined. The shaded amino acid sequences illustrate possible read-through proteins. C. Comparison of potential 'slippery' sequences with the known RFS in HIV-1 *gag-pol*. D and E. Alignment of predicted membrane-spanning proteins encoded in the second overlapping ORFs in the G gene transcriptional units of D) curioviruses and E) some hapaviruses. The predicted initiation codons of independently expressed proteins (bold and underlined), the potential RFSs that would allow a read-through extension of the G proteins (light grey shading) and predicted membrane-spanning regions (dark grey shading) are shown. Amino acid sequence alignments were conducted in Clustal X.

A

```
gtatttcataataatgaaacagatgatgaaagagcaatagagatgatggaatattctgacacaccgagaaccctgcgtcccattc
 V  F  H  N  N  E  T  D  D  E  R  A  I  E  M  M  E  Y  S  D  T  P  R  T  L  R  P  I
                                                               *  W  N  I  L  T  H  R  E  P  C  V  P  F
ctgattcactgccagaaccccaagaagaaactacaagaaacatgtcgcatagtttttcaataggtaaatttgtgttaaaactg
P  D  S  L  P  E  P  Q  E  E  T  T  R  N  M  S  H  S  F  F  N  R  *
 L  I  H  C  Q  N  P  K  K  K  L  Q  E  T  C  R  I  V  F  S  I  G  K  F  V  L  K  L
gtcttgttcatttctccattaatcattataattgtcttctttattctccttaataagaaacatctaatacctatttctataccg

 V  L  F  I  S  P  L  I  I  I  I  V  F  F  I  L  L  N  K  K  H  L  I  P  I  S  I  P

gatataaatcaaactacagtaaaaatggttcccgaatcttaaatataaaaatgtaaaggatttaaagttcatgaaaaaaa

 D  I  N  Q  T  T  V  K  M  V  P  E  S  *
```

B

```
atgaagaacctctcaagctcggtgggagacggtcacatttggttaaaaatccatttttttgacaatgggatttagtattaatttt
   E  E  P  L  K  L  G  G  R  R  S  H  L  V  K  N  P  F  F  D  N  G  I  *
 M  K  N  L  S  S  S  V  G  D  G  H  I  W  L  K  I  H  F  L  T  M  G  F  S  I  N  F
gatccaataattaataaattcagggaattccagaccaatattaatcacaatattaatgaacaactggataagttgaagatggtc

 D  P  I  I  N  K  F  R  E  F  Q  T  N  I  N  H  N  I  N  E  Q  L  D  K  L  K  M  V
tggattaatttgggatctcatattaaatattggtttattattataattagtatattaactatattattcatattatttcttttg

 W  I  N  L  G  S  H  I  K  Y  W  F  I  I  I  I  S  I  L  T  I  L  F  I  L  F  L  L
attaagataactaagcttattttaaattgtaagaagatattttcctgttgttgtaatgtctgttgtaaaaagaggcctaaggtt

 I  K  I  T  K  L  I  L  N  C  K  K  I  F  S  C  C  C  N  V  C  C  K  K  R  P  K  V
gacattaggtcaaaggagaaagtaaaggttttctcgatattaccttgaaaaaaa

 D  I  R  S  K  E  K  V  K  V  F  S  I  L  P  *
```

C

HARV	UAGUUUUUUCAAUCGUUAA	WONV	CCAUUUUUUGACAAUGGGA
BGV	UAGUUUUUUCAAUCGUUAA	ORDV	CCUUUUUUUGAUCAUGGGA
MSV	UAGUUUUUUCAAUAGGUAA	PCV	CCUUUUUUUGAUCAUGGGA

HIV-1 UAAUUUUUUAGGGAAGAUC

D

```
HARV_Gx     W*NILTLLGHCDQSLMPQHQILSQDPPQQPIVFSIVKKISYLILFSIPLIVIIIFFVLLDRKHLKYIPIKUW
BGV_Gx      W*NILTLPGHCDRFLMPQHQULSQDPPQQPIVFSUVKKVGYIILFSIPLIVIIVFFVLLDRKHLKYIPLKAW
MSV_Gx      W*NILUHREPCVPFLIHCQNPKKKLQEUCRIVFSIGKFVLKLVLFISPLIIIIVFFILLNKKHLIPISIPDI
            * ***     *   *:  .:  ..      ****  * :  ::**  ***:**:**:**::***  *.:

HARV_Gx     NUSIIEVKESH
BGV_Gx      NUSIIUIKEPY
MSV_Gx      NQUUVKMVPES
            *: : :
```

E

```
ORV_U4      TKNLSSSVRGRQTWCAILFLIMGFSINFDPIIDGFREFQQNINGDIDDQLDKIKIIWTNLGTHIKYWFILII
PCV_U4      MRSLFSSGRGVTSR*KILFLIMGFSINFDPIINKFREFQTNINNNINEQLDKIKIIWANLGTHIKYWFILII
WONV_U4     MKNLSSSVGDGHIWLKIHFLTMGFSINFDPIINKFREFQTNINHNINEQLDKLKMVWINLGSHIKYWFIIII
             : * **  .      * ** ***********: ***** *** :*::****:*::* ***:*******:**

ORV_U4      SILIVLAILFLLIKITRLILNCKKIFSCCCDLCCKEK-TSKQRREDKIKVFSILP
PCV_U4      SILIIILAVLFLLIKITRLILNCKKIFSCCCSWCCKKQ-KTQRRKDDKVKIFSITP
WONV_U4     SILTILFILFLLIKITKLILNCKKIFSCCCNVCCKKRPKVDIRSKEKVKVFS---
            *** :* :********:*************. ***:: . . * .:*:*:**
```
