# Supplementary material for: Evolution of Genome Size and Complexity in the Rhabdoviridae
Source: PLoS Pathog. 2015 Feb 13;11(2):e1004664. doi: 10.1371/journal.ppat.1004664 (PMC4334499; doi:10.1371/journal.ppat.1004664)
Supplement: S1 Table — (PDF) [file ppat.1004664.s013.pdf]

Supplementary Table S1. Rhabdoviruses for which genome sequences have been used in this study.

Virus	Strain	Genus	Location of first isolation	Year of isolation	Source species	Other known hosts and vectors (isolation or prevalent antibody)	Genome size (nt)	GenBank accession
Arboretum (ABTV)	LO-121	*Almendravirus**	Puerto Almendras, Loreto, Peru	2009	*Psorophora albigenu* (mosquitoes)		11492	KC994644
Puerto Almendras (PTAMV)	LO-39	*Almendravirus**	Puerto Almendras, Loreto, Peru	2009	*Ochlerotatus fulvus* (mosquitoes)		11876	KF534749
Muir Springs (MSV)	76V-23524	*Bahiavirus**	Fort Morgan, Colorado, USA	1976	*Aedes spp.* (mosquitoes)		12580	KM204990§
Bahia Grande (BGV)	TB4-1054	*Bahiavirus**	Brownsville, Texas, USA	1974	*Aedes solicitans* (mosquitoes)		12639	KM205018§
Harlingen (HARV)	PV01-3828	*Bahiavirus**	Harlingen, Texas, USA	2001	*Culex salinarius.* (mosquitoes)		12626	KM205003§
Moussa (MOUV)	D24	unassigned	Taï National Park, Côte d'Ivoire	2004	*Culex decens* (mosquitoes)		11526	FJ985749
New Minto (NMV)	579	*Sawgravirus**	New Minto, Alaska	1972	*Haemaphysalis leporispalustris* (ticks) removed from *Lepus americanus* (hare)		11156+	KM205009§
Connecticut (CNTV)	Ar1152-78	*Sawgravirus**	Lyma, Connecticut, USA	1978	*Ixodes dentatus* (ticks)	*Sylvilagus floridanus* (rabbit)ˣ	11169+	KM205020§
Sawgrass (SAWV)	64A-1247	*Sawgravirus**	Tampa Bay, Florida	1964	*Dermacentor variabilis* (ticks) from racoon	*Haemaphysalis leporispalustris* (ticks)	11216	KM205013§
Ikoma (IKOV)	RV2508	*Lyssavirus*	Serengeti National Park, Tanzania	2009	*Civettictis civetta* (African civet)		11902	JX193798
West Caucasian bat (WCBV)		*Lyssavirus*	Krasnodar, West Caucasus	2001	*Miniopterus schreibersii* (common long-fingered bat)		12278	EF614258
Shimoni bat (SBV)		*Lyssavirus*	Kenya	2009	*Hipposideros commersoni* (Commerson's leaf-nosed bat)		12045	GU170201
Lagos bat (LBV)	0406SEN	*Lyssavirus*	Senegal	1985	*Eidolon helvum* (fruit bat)	*Rousettus aegyptiacus*, *Eidolon helvum*, *Epomophorus wahlbergi*, *Micropterus pusillus*, *Nycteris cambiensis* (bats); *Canis lupus familiaris* (dogs); *Felis cattus* (cats); *Atilax paludinosus* (mongoose)	12016	NC020807
Mokola (MOKV)	RV1035	*Lyssavirus*	Zimbabwe	1982	*Felis cattus* (domestic cat)	*Crocidura spp.* (shrews); *Canis lupus familiaris* (dogs); *Homo sapiens* (humans); *Lophuromys sikapusi* (rodents)	11939	NC006429
Rabies (RABV)	HN10	*Lyssavirus*	Hunan, China	2006	*Homo sapiens*	Raccoons; skunks; foxes; coyotes; bats; domesticated dogs and cats; humans, etc.	11932	EU643590
Aravan (ARAV)		*Lyssavirus*	Aravan, Kyrgyzstan	1991	*Myotis blythi* (lesser mouse-eared bat)		11918	EF614259

Khujand (KHUV)		*Lyssavirus*	Khujand, Tajikstan	2001	*Myotis mystacinus* (whiskered bat)		11903	EF614261
European bat lyssavirus 2 (EBLV2)	RV1333	*Lyssavirus*	United Kingdom	2002	*Homo sapiens* (human)	*Myotis daubentonii. M. dasycneme* (bats)	11930	NC009528
Australian bat lyssavirus (ABLV)		*Lyssavirus*	Queensland, Australia	1996	*Saccolaimus flaviventris* (yellow bellied sheath tail bat)	*Pteropus alecto, P. poliocephalus, P. scapulatus, P. conspicillatus* (bats); *Homo sapiens* (humans)	11918	AF081020
Irkut (IRKV)		*Lyssavirus*	Irkutsk, Eastern Siberia	2002	*Murina leucogaster* (greater tube-nosed bat)		11980	EF614260
Ozernoe (OZEV)		*Lyssavirus*	Russia	2007	*Homo sapiens* (human)		11980	FJ905105
Duvenhage (DUVV)	86132SA	*Lyssavirus*	South Africa	1971	*Homo sapiens* (human)	*Miniopterus schreibersi, Nycteris thebaica* (bats)	11976	NC020810
European bat lyssavirus 1 (EBLV1)	9395GER	*Lyssavirus*	Germany	1968	*Eptesicus serotinus* (serotine bat)	*Eptesicus isabellinus, Myotis myotis, M. natterei*ˣ (bats); *Ovis aries* (sheep); *Felis cattus* (cats); *Martes foina* (Marten); *Homo sapiens* (humans)	11966	NC009527
North Creek (NORCV)#	954	unassigned	Ballina, NSW	1997	*Culex sitiens* (mosquitoes)		partial	KF360973
Drosophila melanogaster sigmavirus (DMelSV)	HAP23	*Sigmavirus*	France	unknown	*Drosophila melanogaster* (flies)		12390+	GQ375258
Drosophila obscura sigmavirus (DObSV)	10A	*Sigmavirus*	United Kingdom	2007	*Drosophila obscura* (flies)		12676+	NC022580
Nkolbisson (NKOV)	YM 31-65	*Ledantevirus**	Nkolbisson, Cameroon	1965	*Eretmapodites leucopus* (mosquitoes)	*Eretmapodites chrysogaster*, *Eretmapodites* sp., *Culex telesilla*, *Aedes mutilus/argenteopunctatus*, *Aedes cumminsi* (mosquitoes); *Homo sapiens* (humans)	10942+	KM205017§
Nishimuro (NISV)		*Ledantevirus**	Nishimuro, Japan		*Sus scrofa* (wild boar)		10881+	AB609604
Barur (BARV)	6235	*Ledantevirus**	Barur, India	1962	*Rattus rattus wroughtoni* (rat)	*Haemaphysalis intermedia* (ticks collected from goats); *Rhipicephalus pulchellus* (ticks collected from camels); mixed pool of mosquitoes; fleas collected from white-toothed shrew (*Crocidura occidentalis*)	10853+	KM204983§
Fukuoka (FUKV)	FUK-11	*Ledantevirus**	Fukuoka, Kyushu, Japan	1982	*Culicoides punctatus (midges)*	*Bos indicus* (cattle); *Culex tritaeniorhynchus (mosquitoes)*	10863	KM205001§
Kern Canyon (KCV)	M03790	*Ledantevirus**	Kern County, California USA	1956	*Myotis yumanensis* (vesper bat)		11528+	KM204992§
Keuraliba (KEUV)	DakAnD5314	*Ledantevirus**	Saboya, Senegal	1968	*Tatera kempi* (gerbil)	*Mastomys* sp., *Taterillus* sp. (rodents)ˣ	11457+	KM205021§

Le Dantec (LDV)	DakHD763	*Ledantevirus**	Le Dantec Hospital, Senegal	1965	*Homo sapiens*		11450+	KM205006§
Mount Elgon bat (MEBV)	BP846	*Ledantevirus**	Kimilili-Bungoma, Kenya	1964	*Rhinolophus hilderbrandtii eloquens* (horseshoe bat)		10941+	KM205026§
Oita (OITAV)	296-1972	*Ledantevirus**	Oita, Japan	1972	*Rhinolophus cornutus* (little Japanese horseshoe bat)		11355+	KM204998§
Fikirini (FKRV)	KEN352	*Ledantevirus**	Shimoni region, South-eastern Kenya	2011	*Hipposideros vittatus* (striped leaf-nosed bat)		11139+	KC676792
Kolente (KOLEV)	DakArK7292	*Ledantevirus**	Kindia region, Guinea	1985	*Amblyomma variegatum* (ticks)	*Hipposideros jonesi* (roundleaf bat)	11120	KC984953
Siniperca chuatsi rhabdovirus (SCRV)		*Perhabdovirus*	China	unknown	*Siniperca chuatsi* (mandarin fish)		11545	NC008514
Eel virus X (EVEX)	CVI153311	*Perhabdovirus*	Netherlands	1992	*Anguilla anguilla* (European eel)		11806	NC022581
Perch rhabdovirus (PRV)		*Perhabdovirus*	France	1980	*Perca fluviatilis* (European perch)		11487+	NC020803
Pike fry rhabdovirus (PFRV)	F4	*Sprivivirus*	France	1972	*Esox lucius* (northern pike)		11097	FJ872827
Spring viremia of carp (SVCV)	S/30	*Sprivivirus*	Croatia	1969	*Cyprinus carpio* (common carp)		11019	NC002803
Radi (RADIV)	ISS Phl-166	*Vesiculovirus*	Radi, Tuscany, Italy	1982	*Phelbotomus perfiliewi* (sandflies)		11068+	KM205024§
Jurona (JURV)	BeAr40578	*Vesiculovirus*	Para, Brazil	1962	*Haemagogus* sp. (mosquitoes)		11121+	KM204996§
Malpais Spring (MSPV)	85-488NM	*Vesiculovirus*	Malpais Lava flow, New Mexico, USA	1985	*Aedes campestris* (mosquitoes)	*Psorophora signipennis* (mosquitoes)	11019	KC412247
Isfahan (ISFV)	91026-167	*Vesiculovirus*	Dormian Village, Isfahan, Iran	1975	*Phlebotomus papatasi* (sandfly)	*Hyalomma asiaticum* (ticks), *Aedes caspius* (mosquitoes); *Homo sapiens* (humans)ˣ; *Rhombomys optimus* (rodents)ˣ	11088	NC020806
Perinet (PERV)	DakArMg802	*Vesiculovirus*	Perinet, Madagascar	1978	*Culex antennatus* (mosquitoes)	*Culex quinquefasciatus*, *Mansonia uniformis*, *Anopheles coustani*, *Anopheles fusicolor* (mosquitoes); *Phlebotomus berentiensis* (sandflies)	11103+	HM566195
Chandipura (CHNV)	CIN0451	*Vesiculovirus*	Gujarat, India	2004	*Homo sapiens* (human)	*Phlebotomus* sp. (sandflies); *Atelerix spiculus*, *A. albiventris* (hedgehogs); camelsˣ; *Equus africanus asinus* , *Equus ferus* (equids)ˣ; *Capra aegagrus hircus* (goats)ˣ; *Bubalis bubalis* (buffalo)ˣ;*Bos taurus* (cattle)ˣ; *Ovis aries* (sheep)ˣ; *Macaca mulatta* (monkeys)ˣ	11120	GU212856

VS New Jersey (VSNJV)	NJ89GAS	*Vesiculovirus*	Georgia, USA	1989	sandflies	Horses; cattle; pigs; humans; *Culex nigripalpis*, *Mansonia indubitans* (mosquitoes); *Musca domestica*, *Musca autumnalis*, Chloropidae, Anthomyidae, Simuliidae (flies); *Culicoides varripennis*, *Culicoides stellifer*, *Culicoides selfia* (sandflies)	11123	JX121110
Carajas (CARV)	BeAr411391	*Vesiculovirus*	Maraba, Para, Brazil	1983	*Lutzomyia* spp. (sandflies)		10716+	KM205015§
Maraba (MARAV)	BeAr411459	*Vesiculovirus*	Maraba, Para, Brazil	1983	*Lutzomyia* spp. (sandflies)		11135	HQ660076
Morreton (MORV)	CoAr191048	*Vesiculovirus*	Durania, Colombia	1986	*Lutzomyia* spp. (sandflies)		11181+	KM205007§
VS Indiana (VSIV)	98COE	*Vesiculovirus*	Colorado, USA	1998	*Equis ferus caballus* (horse)	*Lutzomyia trapidoi*, *Lutzomyia* sp. (sandflies), *Aedes* sp. (mosquitoes); *Homo sapiens* (humans); *Bos taurus* (cattle); *Sus scrofa* (swine)	11161	AF473864
VS Alagoas (VSAV)	Indiana 3	*Vesiculovirus*	Brazil	1964	*Equus caballis/Equus asinus* (mule)	*Homo sapiens* (humans)ˣ, *Equus ferus* (horses)ˣ, monkeysˣ, batsˣ, *Didelphus marsupialis* (possums)ˣ, *Coendou* sp. (porcupines)ˣ, *Canis lupus familiaris* (dogs)ˣ	11070	EU373658
Cocal (COCV)	TRVL40233	*Vesiculovirus*	Trinidad	1961	*Gigantolaelaps* sp. (mites - collected from *Oryzomys laticeps velutinus* - rodent)	*Heteromys anomalus*, *Oryzomys* sp.ˣ, *Zygodontomys brevicauda*ˣ (rodents); sentinel mice; *Equus ferus* (horses); *Culex portesi* (mosquitoes)	11003	EU373657
Durham (DURV)	CC228-C5	*Tupavirus*	Durham county, North Carolina, USA	2005	*Fulica americana* (bird)		11092+	FJ952155
Klamath (KLAV)	M-1056	*Tupavirus**	Klamath Falls, Oregon, USA	1962	*Microtus montanus* (vole)	*Clethrionomys rutilus*, *Microtus oeconomus* (voles)	11478+	KM204999§
Tupaia (TUPV)		*Tupavirus*	Thailand (imported to Germany)	unknown	*Tupaia belangeri* (tree shrew)		11440	NC007020
Chaco (CHOV)	BeAn42217	*Sripuvirus**	Para, Brazil	1962	*Ameiva ameiva ameiva* (lizard)	*Kentropyx calcaratus* (lizard)	11397+	KM205000§
Sena Madureira (SMV)	BeAn303197	*Sripuvirus**	Acre, Brazil	1976	*Ameiva ameiva ameiva* (lizard)		11422+	KM205004§
Niakha (NIAV)	DakArD88909	*Sripuvirus**	Niakha, Senegal	1992	*Phlebotomus duboscqi* and *Sergentomyia* spp. (sandflies)		11124	KC585008
Sripur (SRIV)	733646	*Sripuvirus**	Sripur, India	1973	*Sergentomiya spp.* (sandflies)		11290+	KM205023§
Kwatta (KWAV)	A-57	unassigned	Paramaribo, Surinam	1964	*Culex* sp. (mosquitoes)	Human; *Presbytis entellus, Macaca radiata* (monkeys)*; Rattus rattus wroughtoni, Rattus blanfordi,* (rodents)*;* *Sancus murinus* (shrews); *Rhinolophus rouxi, Haemaphysalis spinigera, H. turturis,* (ticks)	11211+	KM204985§

Oak Vale (OVRV)	K13965	unassigned	Queensland, Australia	1993	Pool of *Anopheles annulipes* and *Culex edwardsi* (mosquitoes)	*Sus scrofa* (swine)ˣ	11220	JF705877
Garba (GARV)	DakAnB439a	unassigned	Garba, Central African Republic	1970	*Corythornis cristata* (bird)	*Nectarinia pulchella* (birds)	10821+	KM204982§
Sunguru (SUNV)	UG#41	unassigned	Arua, Uganda	2011	*Gallus gallus domesticus* (chicken)		11056	KF395226
La Joya (LJV)	J-134	*Hapavirus**	Pacora, Panama	1958	*Culex dunni* (mosquitoes)	*Callomys callosus*, *Oryzomys* sp., *Mus* sp. (rodents)	15721	KM204986§
Wongabel (WONV)	CS264	*Hapavirus**	Atherton Tablelands, Queensland, Australia	1979	*Culicoides austropalpalis* (midges)	Sea birds (species not specified)ˣ	13196	NC011639
Ord River (ORV)	OR1023	*Hapavirus**	Kunnanurra, Western Australia	1976	*Culex annulirostris* (mosquitoes)		13189+	KM205025§
Parry Creek (PCV)	OR189	*Hapavirus**	Kunnanurra, Western Australia	1973	*Culex annulirostris* (mosquitoes)		13205+	KM204988§
Joinjakaka (JOIV)	AusMK7937	*Hapavirus**	Joinjakaka, Sepik District, Papua-New Guinea	1966	Culicine spp. (mosquitoes)	*Bos taurus* (cattle)ˣ	13155	KM205016§
Ngaingan (NGAV)	MRM14556	*Hapavirus**	Gulf of Carpentaria, Queensland, Australia	1970	*Culicoides spp* (midges)	*Bos taurus* (cattle)ˣ; macropodsˣ	15764	NC013955
Marco (MCOV)	BeAn40290	*Hapavirus**	Utinga Forest, Brazil	1962	*Ameiva ameiva ameiva* (lizard)			KM205005§
Gray Lodge (GLOV)	BFN3187	*Hapavirus**	Butte, California, USA	1971	*Culex tarsalis* (mosquitoes)		12403	KM205022§
Landjia (LJAV)	DakAnB769d	*Hapavirus**	Landjia, Central African Republic	1970	*Riparia paludicola* (bird)		13695+	KM205010§
Manitoba (MANV)	Mn936-77	*Hapavirus**	Morris, Manitoba, Canada	1977	*Culex tarsalis* (mosquitoes)		13784+	KM205008§
Mosqueiro (MQOV)	BeAr185559	*Hapavirus**	Belem, Brazil	1970	*Culex portesi* (mosquitoes)	*Wyeomia* sp., *Mansonia* sp. (mosquitoes)	12957	KM205014§
Flanders (FLAV)	61-7484	*Hapavirus**	Flanders, New York, USA	1961	*Culiseta melanura* (mosquitoes)	*Culex pipiens, Culex tarsalis, Culex salinarius* (mosquitoes); *Seiurus aurocapillus*, *Sturnus vulgaris* (birds)	13038	KM205002§
Hart Park (HPV)	AR7C	*Hapavirus**	Hart Park, California, USA	1955	*Culex tarsalis* (mosquitoes)	*Culex restuans, Culex nigripalpus, Culex pipiens, Culiseta melanura* (mosquitoes); *Xanthocephalus xantho, Passer domesticus, Agelaius tricolor, Carpodacus mexicanus, Seiurus aurocapillus* (birds)	13104	KM205011§
Kamese (KAMV)	MP6186	*Hapavirus**	Moulokota Uganda	1967	*Culex annulioris* (mosquitoes)	*Homo sapiens* (humans)ˣ; *Culex pruina, Culex tigripes* (mosquitoes)	13209	KM204989§

Mossuril (MOSV)	SAAr1995	*Hapavirus**	Lumbo, Mozambique	1959	*Culex sitiens* (mosquitoes)	*Homo sapiens* (humans)[x]; *Papio cynocephalus* (baboons)[x]; *Andropadus virens, Coliuspasser macrourus* (birds)	13106+	KM204993§
Aruac (ARUV)	TRVL9223	unassigned	Melaju Forest, Trinidad	1955	*Trichoprosopon theobaldi* (mosquitoes)	*Wyeomyia* sp., *Psorophora ferox*, *Phoniomyia* sp., *Culex* sp., *Sabethes chloropterus* (mosquitoes); birds[x]	11906+	KM204987§
Inhangapi (INHV)	BeAr177325	unassigned	Catu forest, Belem, Brasil	1969	*Lutzomyia flaviscutellata* (sandflies)	*Proechimys guyannensis*[x], *Oryzomys capito goeldii*, *Coendou* sp. (rodents)[x]	12026	KM204991§
Itacaiunas (ITAV)	BeAr427036	*Curiovirus**	Serra Norte, Para, Brazil	1984	*Culicoides sp* (midges)		12536+	KM204984§
Iriri (IRIRV)	BeAr408005	*Curiovirus**	Altamira, Para, Brasil	1982	*Lutzomyia spp.* (sandflies)		13070	KM204995§
Curionopolis (CURV)	BeAr440009	*Curiovirus**	Serra Norte, Para, Brazil	1985	*Culicoides sp.* (midges)	*Cebus apella* (monkeys)[x]; *Nasua nasua* (coatis)[x]	13170	KM204994§
Rochambeau (RBUV)	CaAr16102	*Curiovirus**	Paramana, French Guiana	1973	*Coquillettidia albicosta* (mosquitoes)		13593	KM205012§
Bas Congo (BASV)		*Tibrovirus**	Mangala, Democratic Republic of Congo	2009	*Homo sapiens* (human)		11892+	JX297815
Coastal Plains (CPV)	DPP53	*Tibrovirus*	Coastal Plains, Northern Territory, Australia	1981	*Bos taurus* (cattle)	*Bubalis bubalis* (buffalo)[x]	13203	GQ294473
Sweetwater Branch (SWBV)	UF-11	*Tibrovirus**	Key West, Florida, USA	1982	*Culicoides insignis* (midges)	*Bos taurus* (cattle)[x]	13141+	KM204997§
Bivens Arm (BAV)	UF-10	*Tibrovirus**	Key West, Florida, USA	1982	*Culicoides insignis* (midges)	*Bos taurus* (cattle)[x]	13288+	KM205019§
Tibrogargan (TIBV)	CS132	*Tibrovirus*	Peachester, Queensland, Australia	1976	*Culicoides brevitarsis* (midges)	*Bos taurus* (cattle)[x]; *Bubalis bubalis* (buffalo)[x]	13298	NC020804
Yata (YATV)	DakArB2181	*Ephemerovirus**	Birao, Central African Republic	1969	*Mansonia uniformis* (mosquitoes)		14479	KM085030§
Koolpinyah (KOOLV)	DPP833/819	*Ephemerovirus**	Berrimah, Northern Territory, Australia	1986	*Bos taurus* (cattle)		16133	KM085029§
Kotonkan (KOTV)	IbAr23380	*Ephemerovirus*	Ibadan, Nigeria	1967	*Culicoides spp* (midges)	*Bos taurus* (cattle); *Athelerix albiventris* (hedgehogs)[x]; *Cricetomys gambianus* (rodents)[x]; *Equus ferus* (horse)[x]; *Ovis aries* (sheep)[x]; *Bubulcus ibis* (birds)[x]; *Homo sapiens* (humans)[x]	15870	HM474855
Adelaide River (ARV)	DPP61	*Ephemerovirus*	Tortilla Flat, Northern Territory, Australia	1981	*Bos taurus* (cattle)		14627	JN935380
Obodhiang (OBOV)	SudAr1154-64	*Ephemerovirus*	Malakal, Sudan	1963	*Mansonia uniformis* (mosquitoes)		14717	HM856902

Kimberley (KIMV)	CS368	*Ephemerovirus*	Tortilla Flat, Northern Territory, Australia	1980	*Bos taurus* (cattle)	*Culicoides brevitarsis* (midges); *Mansonia uniformis*, *Culex annulirostris* (mosquitoes);	15442	JQ941664
Bovine ephemeral fever (BEFV)	CS1865	*Ephemerovirus*	Kowanyama, Queensland, Australia	1969	*Bos taurus* (cattle)	*Anopheles bancroftii* (mosquitoes); *Culicoides, brevitarsis* and *Culicoides coarctacus* (midges); *Bos indicus* and *Bos javanicus* (cattle); *Bubalus bubalis* (buffalo); many wild African ruminant species[×]	14900	NC002526
Berrimah (BRMV)	DPP63	*Ephemerovirus*	Berrimah, Northern Territory, Australia	1981	*Bos taurus* (cattle)		15024	HM461974

* Taxonomic assignments proposed in this paper.
# Partial L gene sequences; included only for phylogenetic analysis.
+ Extreme terminal sequences not determined.
× Based on sero-neutralisation or complement fixation data only
na - not applicable.
§ Sequenced in this study.
