# Supplementary material for: Evolution of Genome Size and Complexity in the Rhabdoviridae
Source: PLoS Pathog. 2015 Feb 13;11(2):e1004664. doi: 10.1371/journal.ppat.1004664 (PMC4334499; doi:10.1371/journal.ppat.1004664)
Supplement: S2 Table — (PDF) [file ppat.1004664.s014.pdf]

Supplementary Table S2. Characteristics of possible animal rhabdovirus accessory proteins encoded in ORFs ≥ 180 nt.

Virus	Protein	Size (kDa)*	Homologues	Features	Kozak context[#]	ORF location	Expression probability[¶]
Almendravirus							
ABTV	U1	8.9	PTAMV U1	transmembrane domain; viroporin-like	GUAAUGG (s)	independent transcription	H
PTAMV	U1	9.5	ABTV U1	transmembrane domain; viroporin-like	AAUAUGG (s)	independent transcription	H
Bahiavirus							
MSV	Gx	5.8	BGV,HARV Gx	transmembrane domain	na	overlapping - possible RFS	H
HARV	Nx	7.6	BGV Nx	cysteine-rich	CUUAUGG (m)	alternative - distal	M
	Gx	6.1	MSV, BGV Gx	transmembrane domain	CUGAUGC (w)	overlapping - possible RFS	H
BGV	Nx	7.6	HARV Nx	cysteine-rich	CUUAUGG (m)	alternative - distal	M
	Gx	6.0	MSV, HARV Gx	transmembrane domain	CUGAUGC (w)	overlapping - possible RFS	H
**Unassigned**							
MOUV	none						
Sawgravirus							
NMV	Lx	10.4	none	none	GUUAUGU (m)	alternative - distal	L
CNTV	Nx	9.3	none	none	ACAAUGG (s)	alternative - proximal	H
	Px	12.4	none	highly basic	AGCAUGC (m)	alternative - central	H
	Gx	15.8	none	basic; ser- pro-rich N-terminal domain	UGUAUGC (w)	alternative - proximal	H
	Gy	7.5	none	none	GGAAUGC (m)	alternative - central	M
	Lx	6.7	none	basic	UAUAUGG (m)	alternative - distal	L
SAWV	Gx	7.6	none	pro-rich	CAUAUGA (w)	alternative - proximal	L
	Lx	10.8	none	none	GUCAUGC (m)	alternative - central	L
Lyssavirus							
IKOV	none						
WCBV	Mx	8.4	none	basic	GUGAUGA (s)	alternative - proximal	H
	Gx	7.1	none	basic, cysteine-rich	UGCAUGA (m)	consecutive	L
SBV	none						
LBV	Nx	9.1	none	basic	CUUAUGA (m)	alternative - central	M
	Mx	6.8	none	basic	ACAAUGC (m)	overlapping	L
MOKV	Gx	6.7	none	none	GGGAUGU (m)	alternative - distal	L
RABV	none						
ARAV	Nx	8.0	none	basic	CUGAUGA (m)	alternative - proximal	M
	Lx	7.1	none	highly basic	GGGAUGC (m)	alternative - distal	L
KHUV	Gx	8.5	ABLV, DUVV, EBLV2, IRKV, OZEV Gx	highly basic	UGGAUGC (w)	alternative - central	M
EBLV2	Nx	7.1	none	highly basic	UGAAUGC (w)	alternative - distal	L
	Gx	6.9	ABLV, DUVV, IRKV, KHUV, OZEV Gx	basic	UAAAUGG (m)	alternative - central	M
ABLV	Gx	8.4	DUVV, IRKV, OZEV, EBLV2, KHUV Gx	basic	CUUAUGA (m)	alternative - central	M
	Lx	80	EBLV1 Lx	highly basic	UGUAUGA (m)	alternative - central	M
	Ly	8.3	none	basic	GAGAUGA (s)	alternative - central	M
IRKV	Gx	9.0	ABLV, DUVV, EBLV2, OZEV, KHUV Gx	basic	CCUAUGA (m)	alternative - central	M
OZEV	Gx	8.9	ABLV, DUVV, IRKV, KHUV, EBLV2 Gx	highly basic	UGGAUGC (w)	alternative - central	M
DUVV	Gx	7.6	ABLV, OZEV, IRKV, KHUV, EBLV2 Gx	basic	UGGAUGC (w)	alternative - central	M
	Lx	8.6	none	basic	AAGAUGA (s)	alternative - central	L
EBLV1	Px	6.9	none	highly basic	GAGAUGG (s)	alternative - central	M
	Gx	7.2	none	none	GGGAUGC (m)	alternative - proximal	M
	Lx	8.0	ABLV Lx	highly basic	UGUAUGA (m)	alternative - central	M

Sigmavirus

DMeSV	U1 (X)	33.7	DObSV X	signal peptide	ACAAUGU (m)	independent transcription	H
	Lx	9.9	none	basic	GUCAUGU (m)	alternative - central	L
	Ly	10.8	none	basic; signal peptide	UGAAUGG (m)	alternative - distal	M
DObSV	U1 (X)	36.9	DMeSV X	signal peptide	AAAAUGU (m)	independent transcription	H

Ledantevirus

NKOV	none						
NISV	Nx	8.6	none	none	AGUAUGA (s)	alternative - proximal	H
BARV	Gx	8.5	none	basic	CACAUGA (m)	alternative - central	M
	Lx	6.8	none	basic	UUGAUGA (w)	alternative - central	L
FUKV	Mx	10.4	none	double-membrane-spanning; basic	GCAAUGA (s)	alternative - proximal	H
KCV	U1	8.8	none	none	AAGAUGU (m)	independent transcription	H
KEUV	U1	7.3	LDV U1	none	AAGAUGU (m)	independent transcription	H
	Gx	7.3	none	basic	GCAAUGG (s)	alternative - central	H
LDV	U1	7.4	KEUV U1	none	AAGAUGU (m)	independent transcription	H
	Nx	7.3	none	highly basic	UCGAUGU (w)	alternative - central	**L**
MEBV	None						
OITV	Lx	11.1	none	basic	UUCAUGG (m)	alternative - central	M
FRKV	Gx	10.8	none	none	AGGAUGA (s)	alternative - central	M
	Gy	9.1	none	basic	UGGAUGC (w)	alternative - distal	L
	Lx	7.4	none	acidic	AAUAUGU (m)	alternative - distal	L
KOLEV	Px	7.7	none	highly basic	ACGAUGA (s)	alternative - proximal	H
	Lx	8.5	none	highly basic	GUCAUGC (m)	alternative - central	L

Perhabdovirus

SCRV	Px	8.5	none	basic	GAAAUGG (s)	alternative - proximal	H
	Lx	13.4	none	none	CAAAUGC (w)	alternative - central	L
EVEX	Px	7.7	none	highly basic	AAGAUGG (s)	alternative - central	H
	Lx	9.5	none	acidic	GGCAUGG (s)	alternative - distal	M
PRV	Lx	7.7	none	none	CGAAUGG (m)	alternative - proximal	M

Sprivivirus

PFRV	Lx	7.7	none	none	CGAAUGG (m)	alternative - proximal	M
SVCV	none						

Vesiculovirus

RADV	Nx	11.0	none	transmembrane domain; highly basic	AGAAUGC (m)	alternative - distal	M
	Gx	7.9	none	basic	GGUAUGG (s)	alternative - proximal	H
	Gy	10.4	none	acidic	AGAAUGC (m)	alternative - distal	L
JURV	Px	8.3	none	highly basic	UGAAUGA (m)	alternative - proximal	M
	Lx	7.5	none	basic	AUUAUGC (m)	alternative - central	L
MSPV	Nx	7.2	none	highly basic	UAUAUGA (m)	alternative - proximal	M
	Mx	7.4	none	basic	GACAUGG (s)	alternative - distal	M
	Gx	9.3	none	transmembrane domain; highly basic	UUGAUGA (m)	alternative - central	M
ISFV	Nx	11.2	none	highly basic	CUUAUGC (w)	alternative - distal	L
	Lx	7.1	none	acidic	AUUAUGG (m)	alternative - proximal	M
PERV	Lx	8.3	none	highly basic	CGAAUGC (w)	alternative - proximal	L
	Ly	7.3	none	none	GAGAUGC (m)	alternative - central	L
	Lz	7.7	none	basic	CGCAUGU (w)	alternative - central	L
CHNV	Px	10.0	none	transmembrane domain; highly basic	AAGAUGA (s)	alternative - proximal	H
	Lx	6.9	none	highly acidic	UUUAUGG (m)	alternative - distal	L
VSNJV	Px	8.0	none	highly basic	CUUAUGA (m)	alternative - proximal	H

	Lx	7.7	none	transmembrane domain; basic	UGCAUGG (m)	alternative - central	M
CARV	Nx	6.7	none	basic	AUUAUGC (m)	alternative - central	M
	Gx	8.7	none	highly basic	UAGAUGA (m)	alternative - central	M
	Lx	7.4	none	none	UGGAUGG (m)	alternative - distal	L
MARAV	Gx	13.9	none	highly basic	CGGAUGC (w)	alternative - central	L
MORV	Gx	7.0	none	highly basic	AAAAUGG (s)	alternative - central	M
	Lx	7.7	none	basic	UAGAUGG (m)	alternative - distal	L
VSIV	Nx	7.8	none	none	UCAAUGA (m)	alternative - central	M
	Px	7.9	none	highly basic	UAGAUGA (m)	alternative - proximal	H
	Gx	7.1	none	none	ACCAUGU (m)	alternative - central	M
VSAV	Lx	8.0	none	None	GGCAUGC (m)	alternative - distal	L
COCV	Nx	7.9	none	none	AAGAUGU (m)	alternative - proximal	M
	Ny	7.7	none	basic	UUUAUGA (m)	alternative - central	L
	Px	11.5	none	highly basic	UGGAUGA (m)	alternative - proximal	H
	Py	8.0	none	highly basic	CTGAUGU (w)	alternative - distal	L
	Mx	7.5	none	basic	CAUAUGA (m)	alternative - central	M
	Lx	9.4	none	highly basic	AUUAUGA (s)	alternative - central	M
	Ly	6.7	none	basic	UUCAUGU (w)	alternative - distal	L
	Lz	7.9	none	basic	AGAAUGG (s)	alternative - distal	M
	Lzz	7.0	none	acidic	AACAUGG (s)	alternative - distal	M

Tupavirus

DURV	Px	15.7	KLAV Px; TUPV Px	none	AUUAUGA (s)	alternative - proximal	H
	U1 (SH)	9.2	KLAV U1; TUPV U1; GARV U1; SUNV U1	small hydrophobic	AGGAUGU (m)	independent transcription	H
	Gx	10.7	none	basic	UGAAUGU (w)	alternative - central	
KLAV	Px	24.1	DURV Px; TUPV Px	basic	AUUAUGA (s)	alternative - proximal	H
	U1 (SH)	10.8	DURV U1; TUPV U1; GARV U1; SUNV U1	small hydrophobic	GCGAUGC (w)	independent transcription	H
	Gx	8.0	OVRV Gy	highly basic	UGUAUGA (m)	alternative - distal	L
	U2	6.5	none	basic	AUCAUGA (s)	independent transcription	H
TUPV	Px	25.8	DURV Px; KLAV Px	basic	GGUAUGA (s)	alternative - proximal	H
	Mx	8.2	none	highly basic	CCAAUGA (m)	alternative - proximal	M
	U1 (SH)	10.6	DURV U1; KLAV U1; GARV U1; SUNV U1	small hydrophobic	AAGAUGA (s)	independent transcription	H

Sripuvirus

CHOV	U1	13.6	SEMAV U1; CHOV P; SMV P	highly acidic	AAAAUGG (s)	independent transcription	H
	Mx	9.5	SRIV Mx; NAIV Mx; SMV Mx	small hydrophobic	UAAAUGA (m)	consecutive - TURBS	H
	Gx	10.9	SRIV Gx; NAIV Gx SMV Gx;	transmembrane domain	AAUAUGG (s)	alternative - proximal	H
SMV	U1	13.7	CHOV U1; SMV P; CHOV P	highly acidic	AACAUGU (m)	independent transcription	H
	Mx	10.7	NIAV Mx; CHOV Mx; SMV Mx	small hydrophobic	GAAAUGA (s)	consecutive - TURBS	H
	Gx	10.7	SRIV Gx; CHOV Gx; NAIV Gx	transmembrane domain	AGUAUGG (s)	alternative - proximal	H
NIAV	Mx	9.4	SRIV Mx; CHOV Mx; SMV Mx	small hydrophobic	ACCAUGA (s)	consecutive - TURBS	H
	Gx	10.8	SRIV Gx; CHOV Gx; SMV Gx	transmembrane domain	GAUAUGG (s)	alternative - proximal	H
	Lx	6.4	none	highly basic	GCGAUGU (m)	alternative - proximal	L
SRIV	Nx	7.3	none	phe-trp-rich C-terminal domain	UCUAUGG (m)	alternative - proximal	M
	Ny	7.6	none	none	UAAAUGC (w)	alternative - distal	L
	U1	8.0	none	phe-tyr-rich domain	AAAAUGU (m)	independent transcription	H
	Px	21.9	none	highly basic	CAGAUGA (m)	alternative - proximal	H
	Mx	9.7	NIAV Mx; CHOV Mx; SMV Mx	small hydrophobic	ACCAUGA (s)	consecutive - TURBS	H
	Gx	11.1	NAIV Gx; CHOV Gx; SMV Gx	transmembrane domain	GUUAUGG (s)	alternative - proximal	H

Unassigned

KWAV	Nx	9.2	none	none	CAGAUGC (w)	alternative - proximal	L
	Px	17.6	OVRV Px	pro-rich C-terminal domain	UGGAUG (w)	alternative - proximal	H
	U1 (SH)	7.1	OVRV U1	small hydrophobic	ACAAUGG (s)	independent transcription	H
OVRV	Nx	13.2	none	none	GGCAUGU (m)	alternative - distal	M

	Px	10.4	KWAV Px	none	AAGAUGA (s)	alternative - proximal	
	Py	5.8	none	none	AGGAUGG (s)	alternative - distal	M
	U1 (SH)	6.8	KWAV U1	small hydrophobic	AAGAUGU (m)	independent transcription	H
	Gx	9.8	none	none	UCUAUGU (w)	alternative - proximal	L
	Gy	7.5	none	basic	AAGAUGU (m)	alternative - central	M
	Gz	6.6	none	highly basic	GACAUGG (s)	alternative - distal	M
GARV	Px	12.4	SUNV Px	none	AGAAUGA (s)	alternative - proximal	H
	U1 (SH)	8.7	DURV U1; KLAVU1; TUPV U1; SUNV U1	small hydrophobic	AUCAUGA (s)	independent transcription	H
SUNV	Px (C)	15.3	GARV Px	acidic	UUGAUGA (m)	alternative - proximal	H
	U1 (SH)	9.3	DURV U1; KLAVU1; TUPV U1; GARV U1	small hydrophobic	ACCAUGA (s)	independent transcription	H

Hapavirus

LJV	U1	17.5	other hapavirus PMIPs§	none	AAAAUGG (s)	independent transcription	H
	U2	18.9	other hapavirus PMIPs	acidic	AGUAUGG (s)	independent transcription	H
	U3	17.9	other hapavirus PMIPs	basic	AUCAUGU (m)	independent transcription	H
	Mx	7.6	none	none	UACAUGA (m)	alternative - distal	M
	U4	13.9	none	acidic	AUCAUGC (m)	independent transcription	H
	U5	18.2	none	none	AUCAUGG (s)	independent transcription	H
	Gx	15.6	none	highly basic	AUCAUGA (s)	alternative - proximal	H
	U6	14.1	none	transmembrane domain; viroporin-like	AUCAUGG (s)	independent transcription	H
	U7	37.5	none	none	AUCAUGG (s)	independent transcription	H
	U7x	7.9	none	double-membrane-spanning	GCAAUGA (s)	alternative - distal	M
WONV	Nx (U4)	5.8	ORV, PCV Nx	highly acidic	AUGAUGC (m)	overlapping; possible RFS	H
	U1	21.2	other hapavirus PMIPs	acidic	AAGAUGG (s)	independent transcription	H
	U2	21.9	other hapavirus PMIPs	acidic	AUCAUGG (s)	independent transcription	H
	U3	16.5	other hapavirus PMIPs	acidic	AUCAUGG (s)	independent transcription	H
	Gx	7.4	none	none	CACAUGG (m)	alternative - central	L
	Gy (U5)	14.6	ORV, PCV Gx	transmembrane domain; viroporin-like	UAUAUGA (m)	overlapping; possible RFS	H
ORV	Nx	8.8	WONV, PCV Nx	acidic	CCAAUGC (w)	overlapping; possible RFS	H
	U1	21.4	other hapavirus PMIPs	acidic	ACCAUGG (s)	independent transcription	H
	U2	22.3	other hapavirus PMIPs	acidic	AUCAUGG (s)	independent transcription	H
	U3	16.8	other hapavirus PMIPs	acidic	AUCAUGG (s)	independent transcription	H
	Gx	12.3	PCV Gx; WONV Gy	transmembrane domain; viroporin-like	AUCAUGG (s)	overlapping; possible RFS	H
PCV	Nx	5.8	WONV, ORDV Nx	acidic	UGAAUGA (m)	overlapping; possible RFS	H
	U1	21.2	other hapavirus PMIPs	acidic	AUCAUGG (s)	independent transcription	H
	U2	21.8	other hapavirus PMIPs	acidic	AUCAUGG (s)	independent transcription	H
	U3	16.8	other hapavirus PMIPs	acidic	AUUAUGG (s)	independent transcription	H
	Gx	12.4	ORV Gx; WONV Gy	transmembrane domain; viroporin-like	AUCAUGG (s)	overlapping; possible RFS	H
JOIV	U1	18.7	none	none	AUAAUGG (s)	independent transcription	H
	Gx	14.0	none	transmembrane domain; viroporin-like	CACAUGG (m)	overlapping	H
	U2	15.9	JOIV U3	basic	ATTAUGG (s)	independent transcription	H
	U3	16.0	JOIV U2	basic	GUCAUGG (s)	independent transcription	H
NGAV	Px	7.7	none	basic	UUGAUGC (w)	alternative - proximal	M
	U1	15.8	none	none	AUCAUGG (s)	independent transcription	H
	U1x (U2)	15.2	none	none	GAGAUGU (m)	overlapping	M
	U2 (U3)	17.1	none	acidic	UCAAUGG (m)	independent transcription	H
	Mx	10.9	none	none	AGUAUGC (m)	alternative - proximal	M
	U3 (U4)	9.7	none	acidic; phe-tyr-rich	UCAAUGC (w)	independent transcription	H
	Gns	65.0	NGAV G	class I transmembrane glycoprotein	AACAUGU (m)	independent transcription	H
	U4 (U5)	12.6	none	none	AUCAUGG (s)	independent transcription	H
	U4x (U6)	13.0	none	transmembrane domain; viroporin-like	GAAAUGG (s)	consecutive	H
	U5 (U7)	18.2	none	basic	AAUAUGG (s)	independent transcription	H
MCOV	Nx	7.6	none	basic; central TM domain	UAAAUGU (w)	alternative - central	M
	Ny	7.7	none	highly basic	AGGAUGG (s)	overlapping ORF	H
	U1	52.5	none	class I transmembrane glycoprotein	AUCAUGU (m)	independent transcription	H

	U2	11.2	none	tyr-rich and highly acidic regions in C-terminal domain	AAAAUGG (s)	independent transcription	H
GLOV	Px	11.5	none	basic	UUGAUGA (m)	alternative - proximal	M
	U1	17.3	other hapavirus PMIPs	acidic	AUCAUGC (m)	independent transcription	H
	U1x	17.4	other hapavirus PMIPs	none	AGAAUGA (s)	consecutive - TURBS	H
	Gx	12.7	MANV, HPV, LJAV, PCV Gx; KAMV FLAV Gy; INHV ARV U1; TIBV, BAV, SWBV, BASV U3	transmembrane domain; viroporin-like	CUAAUGG (m)	consecutive - TURBS	H
LJAV	Px	9.3	MANV Px	transmembrane domain; highly basic	ACAAUGA (s)	alternative - proximal	H
	U1	18.6	other hapavirus PMIPs	acidic	AAGAUGG (s)	independent transcription	H
	U1x	6.9	none	basic	CAAAUGU (w)	alternative - central	L
	U2	20.0	other hapavirus PMIPs	basic	AUCAUGA (s)	independent transcription	H
	U3	19.3	other hapavirus PMIPs	none	UCAAUGG (m)	independent transcription	H
	Gx	17.6	MANV, ORV Gx; KAMV, MOSV, WONV Gy; INHV U1; SWBV U3	transmembrane domain; viroporin-like	GUAAUGA (s)	consecutive - TURBS	H
	U4	17.8	none	basic	AUCAUGG (s)	independent transcription	H
MANV	Nx	7.9	none	basic	UAGAUGG (m)	alternative - central	M
	Ny	9.3	none	transmembrane domain; highly basic	AUCAUGG (s)	overlapping	H
	Px	10.5	LJAV Px	highly basic	AAGAUGA (s)	alternative - proximal	H
	U1	18.2	other hapavirus PMIPs	acidic	AAUAUGG (s)	independent transcription	H
	U1x	19.5	other hapavirus PMIPs	none	UGGAUGU (w)	overlapping; possible RFS	H
	U1y	8.6	none	none	ACUAUGG (s)	alternative - distal	H
	U2	20.0	other hapavirus PMIPs	none	AUAAUGG (s)	independent transcription	H
	U3	19.2	other hapavirus PMIPs	none	AUCAUGA (s)	independent transcription	H
	Gx	13.3	LJAV, HPV Gx; KAMV, MOSV Gy	transmembrane domain; viroporin-like	ACAAUGA (s)	consecutive - TURBS	H
MQOV	Px	9.1	none	highly basic	AAGAUGA (s)	alternative - proximal	H
	U1	18.6	other hapavirus PMIPs	acidic	AAAAUGG (s)	independent transcription	H
	U1x	8.6	none	basic	GAUAUGG (s)	alternative - central	H
	U2	19.2	other hapavirus PMIPs	basic	AUCAUGA (s)	independent transcription	H
	U3	18.1	other hapavirus PMIPs	acidic	AUCAUGA (s)	independent transcription	H
	Gx	7.5	motif shared with sripuvirus Gx	highly basic	UUGAUGG (m)	alternative - proximal	M
	Gy	11.5	MOSV, KAMV, FLAV Gy; HPV, MANV Gx	transmembrane domain; viroporin-like	CUGAUGG (m)	consecutive - TURBS	H
FLAV	Nx	8.5	HPV Nx	basic	CAAAUGC (w)	alternative - distal	M
	U1	18.6	other hapavirus PMIPs	acidic	AAAAUGG (s)	independent transcription	H
	U2	19.0	other hapavirus PMIPs	basic	AAAAUGA (s)	independent transcription	H
	U3	18.9	other hapavirus PMIPs	none	AUCAUGG (s)	independent transcription	H
	Gx	8.5	KAMV Gx	basic	UAAAUGG (m)	alternative - central	M
	Gy	13.8	KAMV, MQOV, MOSV Gy; HPV, MANV, GLOV Gx; INHV, ARV U1; ITAV U2	transmembrane domain; viroporin-like	UUAAUGG (m)	consecutive - TURBS	H
	Lx	7.8	none	basic	ACAAUGC (m)	alternative - distal	L
HPV	Nx	8.9	FLAV Nx	highly basic	AUUAUGA (s)	alternative - distal	M
	U1	18.6	other hapavirus PMIPs	acidic	AAAAUGG (s)	independent transcription	H
	U2	19.2	other hapavirus PMIPs	basic	AGAAUGA (s)	independent transcription	H
	U3	18.9	other hapavirus PMIPs	none	AUCAUGA (s)	independent transcription	H
	U3x	7.2	none	basic	CAAAUGU (w)	alternative - central	L
	Gx	13.6	FLAV, KAMV, MOSV, MQOV Gy; MANV, ORV Gx; BAV U3; INHV U3	transmembrane domain; viroporin-like	CUAAUGG (w)	consecutive - TURBS	H
KAMV	U1	19.0	other hapavirus PMIPs	acidic	AUAAUGA (s)	independent transcription	H
	U2	19.2	other hapavirus PMIPs	basic	AGAAUGA (s)	independent transcription	H
	U2x	11.3	MOSV U2x	basic	AAGAUGU (m)	alternative - proximal	H
	U3	18.8	hapavirus PMIPs	none	AUCAUGA (s)	independent transcription	H
	Gx	8.1	FLAV Gx	signal peptide (possibly secreted)	AAGAUGU (m)	alternative - central	L
	Gy	12.2	FLAV, MOSV, MQOV Gy; HPV, MANV, LJAV Gx; ITAV U2; TIBV, SWBV, BASV U3	transmembrane domain; viroporin-like	CUGAUGG (m)	consecutive - TURBS	H
	Lx	7.8	none	highly basic	GGUAUGU (m)	alternative - central	L
	Ly	7.2	none	highly basic	CACAUGG (w)	alternative - distal	L
MOSV	U1	19.0	other hapavirus PMIPs	acidic	AAAAUGA (s)	independent transcription	H
	U2	19.2	other hapavirus PMIPs	basic	AUCAUGA (s)	independent transcription	H

	U2x	11.6	KAMV U2x	none	AGGAUGU (m)	alternative - proximal	H
	U3	18.7	other hapavirus PMIPs	none	AUCAUGA (s)	independent transcription	H
	Gx	9.3	none	basic	AAAATGG (s)	alternative - distal	M
	Gy	12.3	KAMV, FLAV, MQOV Gy; HPV, MANV, LJAV Gx; TIBV, SWBV U3	transmembrane domain; viroporin-like	CUAAUGG (m)	consecutive - TURBS	H
	Lx	7.5	none	none	GGAAUGU (m)		

Unassigned

ARUV	Gx	8.6	none	none	ACAAUGG (s)	alternative - proximal	M
	U1	14.1	IRIRV U3	transmembrane domain; viroporin-like	CUCAUGC (w)	independent transcription	H
	U1x	5.9	none	acidic	AUAAUGC (m)	consecutive - TURBS	H
INHV	Px	7.3	none	highly basic	AGGAUGA (s)	alternative - proximal	M
	U1	12.7	TIBV, BAV U3; HPV Gx; KOOLV U1	transmembrane domain; viroporin-like	AAGAUGA (s)	independent transcription	H
	Lx	7.4	none	basic	CUCAUGA (m)	alternative - central	L

Curiovirus

ITAV	U1	9.9	none	acidic	UCAAUGG (m)	independent transcription	H
	U2	9.3	none	transmembrane domain; viroporin-like	GUCAUGG (s)	independent transcription	H
	Lx	7.6	none	acidic	CGAAUGC(w)	alternative - distal	L
IRIRV	U1	9.5	CURV U1, RBUV U1	acidic	AAGAUGG (s)	independent transcription	H
	U1x	7.6	CURV, RBUV U1x	acidic	AUCAUGA (s)	overlapping; possible RFS	H
	U2	11.7	CURV U2	basic	ACCAUGG (s)	independent transcription	H
	Gx	7.0	none	basic	AAAAUGU (m)	alternative - proximal	M
	U3	9.8	CURV U3; RBUV U4	transmembrane domain; viroporin-like	CCGAUGG (m)	independent transcription	H
	U3x	13.6	CURV 3x; RBUV 4x	basic	AAAAUGA (s)	consecutive - partial TURBS	H
CURV	U1	9.3	IRIRV U1, RBUV U1	acidic	AAGAUGG (s)	independent transcription	H
	U1x	8.2	IRIRV U1x, RBUV U1x	acidic	ACCAUGA (s)	overlapping; possible RFS	H
	U2	11.5	IRIRV U2	basic	ACCAUGG (s)	independent transcription	H
	U3	10.9	IRIRV U3; RBUV U4	transmembrane domain; viroporin-like	CCGAUGG (m)	independent transcription	H
	U3x	13.3	IRIRV 3x; RBUV 4x	basic	AAAAUGA (s)	consecutive - TURBS	H
RBUV	U1	9.3	IRIRV U1, CURV U1	acidic	AAGAUGG (s)	independent transcription	H
	U1x	8.6	IRIRV, CURV U1x	acidic	AUCAUGA (s)	overlapping; possible RFS	H
	U2	10.0	none	acidic; signal peptide	GGAAUGA (s)	independent transcription	H
	U3	15.2	none	central transmembrane domain	CCCAUGU (w)	independent transcription	H
	U4	10.9	IRIRV, CURV, BAV U3;	transmembrane domain; viroporin-like	CCGAUGG (m)	independent transcription	H
	U4x	13.4	IRIRV, CURV U3x	basic	ACAAUGA (s)	consecutive - partial TURBS	H
	Lx	9.4	none	basic	GUCAUGG (s)	alternative - distal	L

Tibrovirus

BASV	Nx	7.4	none	basic	UCGAUGU (w)	alternative - proxima	L
	U1	23.8	none	none	ACGAUGG (s)	independent transcription	H
	U2	19.9	none	basic	AAAAUGG (s)	independent transcription	H
	Gx	9.1	none	highly basic	AAGAUGC (m)	alternative - central	L
	U3	10.8	none	transmembrane domain; viroporin-like	ACUAUGC (m)	independent transcription	H
CPV	U1	19.7	TIBV, SWBV, BAV U1	acidic	GAAAUGG (s)	independent transcription	H
	U2	18.4	TIBV, SWBV, BAV U2	basic	ACAAUGG (s)	independent transcription	H
	U3	12.1	MANV Gx	transmembrane domain; viroporin-like	GUCAUGC (m)	independent transcription	H
SWBV	Px	7.3	none	basic	AGGAUGA (s)	alternative - central	M
	U1	19.4	TIBV, CPV, BAV U1	none	AACAUGG (s)	independent transcription	H
	U2	18.6	TIBV, CPV, BAV U2	none	AUCAUGC (m)	independent transcription	H
	U3	12.7	SWBV U3	transmembrane domain; viroporin-like	GUUAUGG (s)	independent transcription	H
BAV	U1	19.8	TIBV, CPV, SWBV U1	acidic	ACAAUGG (s)	independent transcription	H
	U2	18.3	TIBV, CPV, SWBV U2	basic	AUCAUGG (s)	independent transcription	H

	U3	13.1	TIBV, BAV U3	transmembrane domain; viroporin-like	GCAAUGC (m)	independent transcription	H
TIBV	U1	19.9	CPV, BAV, SWBV U1	acidic	ACAAUGG (s)	independent transcription	H
	U2	17.1	CPV, BAV, SWBV U2	basic	AUCAUGG (s)	independent transcription	H
	U3	12.9	BAV U3	transmembrane domain; viroporin-like	ACAAUGU (m)	independent transcription	H
Ephemerovirus							
YATV	Gns	62.0	YATV G; other rhabdo G; ephemero Gns	class I transmembrane glycoprotein	AAAAUGA (s)	independent transcription	H
	U1 (α1)	11.9	none	transmembrane domain; viroporin-like	ACUAUGC (m)	independent transcription	H
	U2	14.4	none	none	ACAAUGG (s)	independent transcription	H
	U3 (β)	16.5	other ephemero β	none	AAGAUGU (m)	independent transcription	H
	U4	13.8	none	acidic	AAAAUGG (s)	independent transcription	H
KOOLV	Gns	67.8	KOOLV G; other rhabdo G; ephemero Gns	class I transmembrane glycoprotein	GAAAUGG (s)	independent transcription	H
	U1 (α1)	10.6	other ephemero α1	transmembrane domain; viroporin-like	CUAAUGA (m)	independent transcription	H
	U1x (α2)	11.4	other ephemero α2	basic	GAAAUGU (m)	consecutive - partial TURBS	H
	U2 (β)	18.2	other ephemero β	none	AAAAUGA (s)	independent transcription	H
	U3 (γ)	11.7	other ephemero γ	basic	AACAUGA (s)	independent transcription	H
	U4 (δ)	12.2	KOTV U4	acidic	GUCAUGG (s)	independent transcription	H
KOTV	Gns	68.3	KOTV G; other rhabdo G; ephemero Gns	class I transmembrane glycoprotein	AAAAUGA (s)	independent transcription	H
	U1 (α1)	10.6	other ephemero α1	transmembrane domain; viroporin-like	CUCAUGA (m)	independent transcription	H
	U1x (α2)	11.4	other ephemero α2	basic	AGGAUGU (m)	consecutive - TURBS	H
	U2 (β)	18.5	other ephemero β	none	AAAAUGA (s)	independent transcription	H
	U3 (γ)	11.8	other ephemero γ	none	AACAUGA (s)	independent transcription	H
	U4 (δ)	12.4	KOOLV U4	acidic	GUCAUGG (s)	independent transcription	H
ARV	Px	7.4	ARV G; other rhabdo G; ephemero Gns	highly basic	UGAAUGC (w)	alternative - central	M
	Gns	71.0		class I transmembrane glycoprotein	AAAAUGG (s)	independent transcription	H
	U1 (α1)	12.5	other ephemero α1	transmembrane domain; viroporin-like	CUCAUGG (m)	independent transcription	H
	U1x (α2)	12.0	other ephemero α2	none	GACAUGC (m)	consecutive - possible TURBS	H
	U2 (β)	17.1	other ephemero β	none	AUUAUGG (s)	independent transcription	H
OBOV	Gns	70.4	OBOV G; other rhabdo G; ephemero Gns	class I transmembrane glycoprotein	AUCAUGA (s)	independent transcription	H
	U1 (α1)	12.2	other ephemero α1	transmembrane domain; viroporin-like	CUAAUGG (m)	independent transcription	H
	U1x (α2)	11.9	other ephemero α2	basic	AAAAUGU (m)	consecutive - possible TURBS	H
	U2 (β)	17.2	other ephemero β	none	ACUAUGG (s)	independent transcription	H
KIMV	Gns	67.7	KIMV G; other rhabdo G; ephemero Gns	class I transmembrane glycoprotein	AAUAUGU (m)	independent transcription	H
	U1 (α1)	12.5	other ephemero α1	transmembrane domain; viroporin-like	GGAAUGG (s)	independent transcription	H
	U1x (α2)	10.7	other ephemero α2	none	UGGAUGG (m)	consecutive ORF	H
	U2 (β)	16.8	other ephemero β	basic	AUCAUGG (s)	independent transcription	H
	U3 (γ)	13.7	other ephemero γ	basic	AUCAUGG (s)	independent transcription	H
BEFV	Gns	68.8	BEFV G; other rhabdo G; ephemero Gns	class I transmembrane glycoprotein	AUCAUGU (m)	independent transcription	H
	U1 (α1)	10.7	other ephemero α1	transmembrane domain; viroporin-like	GCAAUGG (w)	independent transcription	H
	U1x (α2)	14.2	other ephemero α2	basic	AAAAUGU (m)	consecutive - TURBS	
	U2 (β)	16.9	other ephemero β	basic	AUCAUGG (s)	independent transcription	H
	U3 (γ)	13.5	other ephemero γ	basic	AUCAUGG (s)	independent transcription	H
BRMV	Gns	67.8	BRMV G; other rhabdo G; ephemero Gns	class I transmembrane glycoprotein	AUCAUGU (m)	independent transcription	H
	U1 (α1)	10.8	other ephemero α1	transmembrane domain; viroporin-like	GCAAUGG (s)	independent transcription	H
	U1x (α2)	13.8	other ephemero α2	basic	AGAAUGU (m)	consecutive - TURBS	H
	U2 (β)	17.0	other ephemero β	basic	AUCAUGG (s)	independent transcription	H
	U3 (γ)	13.4	other ephemero γ	basic	AUCAUGG (s)	independent transcription	H

*Estimated for unprocessed proteins. § Hapavirus P-M intergenic region (IGR) proteins (PMIPs) form a homologous set

Strength of Kozak context (s = strong; m = moderate; w = weak)

¶ Estimated based on size and location of the ORF, Kozak context of the initiation codon and evidence of ORF conservation in related viruses (see text)
