# Supplementary material for: Evolution of Genome Size and Complexity in the Rhabdoviridae
Source: PLoS Pathog. 2015 Feb 13;11(2):e1004664. doi: 10.1371/journal.ppat.1004664 (PMC4334499; doi:10.1371/journal.ppat.1004664)
Supplement: S1 Text — (PDF) [file ppat.1004664.s015.pdf]

Assignment of rhabdoviruses to existing genera and proposed new genera

Based on the phylogenetic analysis and consideration of genome organisations and known hosts and vectors, the viruses were assigned to 17 well-supported monophyletic groups which included eight genera approved by the ICTV (*Lyssavirus*, *Vesiculovirus*, *Perhabdovirus*, *Sigmavirus*, *Ephemerovirus*, *Tibrovirus*, *Tupavirus* and *Sprivivirus*) and seven proposed new genera (*Bahiavirus*, *Sawgravirus*, *Almendravirus*, *Sripuvirus*, *Ledantevirus*, *Hapavirus* and *Curiovirus*).

Genera *Ephemerovirus*, *Tibrovirus* and *Tupavirus*. Within the established genera, *Koolpinyah virus* (KOOLV) and *Yata virus* (YATV) can be considered new species in the genus *Ephemerovirus* with which they cluster phylogenetically. Each has a genome organisation similar to other ephemeroviruses, including genes encoding the characteristic non-structural glycoprotein gene (G_{NS}) followed by a viroporin ($\alpha 1$) and several other small proteins. Like some other members of this genus, KOOLV was isolated from a healthy sentinel bovid (*Bos indicus*) and YATV was isolated from mosquitoes (*Mansonia uniformis*). *Bas Congo virus* (BASV), detected in a human with acute haemorrhagic fever, and *Sweetwater Branch virus* (SWBV), isolated from biting midges (*Culicoides insignis*), cluster phylogenetically and share the same genome organisation as existing species in the genus *Tibrovirus* and can be considered new species. Bivens Arm virus (BAV), which was isolated from biting midges (*Culicoided insignis*) and infecting cattle, also falls in this clade. However, it is very closely related to Tibrogargan virus (TIBV) and may be regarded as the same species (*Tibrogargan virus*). *Klamath virus* (KLAV), isolated from voles (*Microtus* and *Clethrionomys* spp.), can be considered a new species in the genus *Tupavirus*, clustering with the species *Tupaia virus* (TUPV) and *Durham virus* (DURV).

Genus *Bahiavirus*. The proposed new genus *Bahiavirus* comprises the species *Muir Spring virus* (MSV) together with the very closely related Bahia Grande virus (BGV) and Harlingen virus (HARV) which can be considered a single species (*Bahia Grande virus*). The bahiaviruses were all isolated from mosquitoes (*Aedes* and *Culex* spp.) and each encodes a viroporin-like protein as an overlapping reading frame at the end of the G gene.

Genus *Sawgravirus.* The proposed new genus *Sawgravirus* is a monophyletic group which is deeply rooted on the same clade as the bahiaviruses. It comprises the proposed species *Sawgrass virus* (SAWV), *Connecticut virus* (CNTV) and *New Minto virus* (NMV), each of which has been isolated from ticks. *Moussa virus* (MOUV), which was isolated from mosquitoes (*Culex decans*) but lacks the viroporin gene, lies on a branch between the bahiaviruses and sawrgaviruses. It is proposed as an unassigned species at this stage.

Genus *Almendravirus*. The proposed new genus *Almendravirus* comprises species *Puerto Almendras virus* (PTAMV) and *Arboretum virus* (ABTV). These viruses, which were each isolated from mosquitoes (*Psorophora albigenu* and *Ochlerotattus fulvus*), form a deeply rooted monophyletic group and each encoded a viroporin-like protein as an independent transcriptional unit following the G gene.

Genus *Sripuvirus*. The proposed new genus *Sripuvirus* comprises five species, *Sripur virus* (SRIV), *Niakha virus* (NIAV), *Chaco virus* (CHOV) and *Sena Madueira virus* (SMV). They form a monophyletic group and share a similar genome architecture featuring a small protein encoded in a consecutive ORF in the M gene and a small transmembrane protein encoded in an alternative ORF at the start of the G gene. SRIV and NIAV have been isolated from Old World sand flies (*Phlebotomus* and *Sergentomyia* spp.) and CHOV and SMV have been isolated from lizards (*Ameiva ameiva ameiva* and *Kentropyx calcaratus*).

Genus *Ledantevirus*. The proposed genus *Ledantevirus* is a large monophyletic group comprising three sub-clades. In the first sub-clade, the proposed species *Kolente virus* (KOLEV), *Fikirini virus* (FKRV), *Oita virus* (OITAV), *Mount Elgon bat virus* (MEBV), have each been isolated from bats (*Myotis*, *Rhinolophus* and *Hipposideros* spp.). The second sub-clade comprises *Kern Canyon virus* (KCV) which was also isolated from a bat (*Myotis yumanensus*), *Keuraliba virus* (KEUV) which was isolated from rodents (*Tatera, Taterillus* and *Mastomys* spp.) and *Le Dantec virus* (LDV) which was isolated from a 10 year-old human female with clinical fever and encephalitis The third sub-clade includes *Nkolbisson virus* (NKOV) which was isolated from mosquitoes (*Eretmapodites, Aedes* and *Culex* spp.), *Nishimuro virus* (NISV) which was isolated from a wild boar (*Sus scrofa*), *Barur virus* (BARV) which has been isolated from rodents (*Rattus* sp.), mosquitoes (mixed pool) and ticks (*Haemaphysalis* and *Rhipicephalus* spp.) that were feeding on goats and camels, respectively, and *Fukuoka virus* (FUKV) which was isolated from mosquitoes (*Culex* sp.), biting midges (*Culicoides* sp.) and cattle in which it has been associated with a mild febrile illness.

Genus *Curiovirus*. The proposed new genus *Curiovirus* comprises a monophyletic group of six viruses isolated from biting midges (*Culicoides* sp.), sandflies (*Lutzomyia* spp.) and mosquitoes (*Coqillettidia* and *Trichoprosopon* spp.) from the forests of South America and the Caribbean. The species *Curionopolis virus* (CURV), *Irini virus* (IRIRV), *Rochambeau virus* (RBUV) and *Itaciaunas virus* (ITAV) have similar genome architecture with one or more genes located between the M and G genes, and the G and L genes. The closely related species *Aruac virus* (ARUV) and *Inhangapi virus* (INHV) are more deeply rooted in the same clade but lack genes between the M and G genes, and therefore we have excluded them from the genus at this time. The only available information on the vertebrate host range of these viruses is the frequent isolation of INHV from rodents *(Proechimys* and *Oryzomys* spp.). We

note the previous suggestion that CURV and ITAV should be assigned to a new genus for which the name *Bracorhabdovirus* was proposed (Brazilian Amazonian *Culicoides* rhabdoviruses) (27). However, our analysis clearly indicates that this monophyletic group has a broader host range and geographic distribution than this proposed genus name suggests.

Genus *Hapavirus*. The proposed genus *Hapavirus* is a large and ecologically diverse monophyletic group, primarily comprising viruses that share the common characteristic of multiple small genes located between the P and M genes. *Marco virus* (MCOV) also lies within the clade (as it does in trees constructed using N gene/protein sequences; not shown) but, uniquely, lacks this genome architecture. There are two well-supported sub-clades within the genus. One comprises the species *La Joya virus* (LJV), *Parry Creek virus* (PCV) and *Ord River virus* (ORV), each isolated from mosquitoes (*Culex* spp.) and *Wongabel virus* (WONV), isolated from biting midges (*Culicoides* sp.). Antibodies to WONV have been detected in sea birds. The second sub-clade includes the species *Mossuril virus* (MOSV), *Kamese virus* (KAMV), *Hart Park virus* (HPV), *Flanders virus* (FLAV), *Mosquiero virus* (MQOV), *Mannitoba virus* (MANV), and *Grey Lodge virus* (GLOV), each isolated from mosquitoes (*Culex, Culiseta*, *Mansonia* and *Wyeomyia* spp.), and *Landjia virus* (LANV) which was isolated from a brown-throated martin (*Riparia paludicola*). HPV, FLAV and MOSV have also been isolated from birds, suggesting that members of this genus are maintained primarily in a mosquito-passerine bird transmission cycle. MCOV, isolated from a lizard (*Ameiva ameiva ameiva*), *Ngaingan virus* (NGAV), isolated from midges (Culicoides spp.), and *Joinjakaka virus* (JOIV), isolated from mosquitoes (Culicine species), also clearly fall within the genus (BSP $\geq$ 85) but their relationships to viruses in the two major sub-clades are not clearly resolved in the tree.

Other unassigned species. The appropriate taxonomic assignments of several viruses in the dataset are presently unclear. Proposed species *Garba virus* (GARV), *Kwatta virus* (KWAV) and *Oak Vale rhabdovirus* (OVRV) and *Sunguru virus* (SUNV) form a monophyletic group that sits in the tree in the same clade as the sripuviruses. However, they share similar genome architecture to the tupaviruses, which are more deeply rooted in the tree, and there is identifiable sequence homology between the GARV SH protein and those of the tupaviruses.
