# Supplementary material for: Evolution of Genome Size and Complexity in the Rhabdoviridae
Source: PLoS Pathog. 2015 Feb 13;11(2):e1004664. doi: 10.1371/journal.ppat.1004664 (PMC4334499; doi:10.1371/journal.ppat.1004664)
Supplement: S5 Fig — (PDF) [file ppat.1004664.s005.pdf]

Figure S5. Amino acid sequence alignments (Clustal X) of A) the U2 and U3 proteins of JOIV (hapavirus), B) the U1 and P proteins of CHOV and SMV (almendraviruses), and C) the U3 and G proteins of CURV and IRIRV (curioviruses), illustrating homologies that provide further evidence of gene duplication. Identical amino acids are shaded.

A

```
JOIV_U2       MAEYCYIHKAKVRVLKGNPFGHPDLFKILRNGGYSDGEKLCVLNSLETRAESIVQEYTKFFNLIHITRKRNEIIRTSDFELMVK
JOIV_U3       MA--CT-KMIKVKLVVGHLSDYENKNRVW-NAIRAQPINFKYKMSL---LRSIYSDPSRWVQTVIETDSHPILYWPATDYVWVK
              **  *  :  **::: *:  .: :  ::  *.  ::  ::    **    .** .: :::.: :  * .:  :  .:   : **

JOIV_U2       IFTSTSLISPPPSLWSQTSRFVKSYFTNKNELCEELNIYPIGIDYESLINYIP-------
JOIV_U3       LRGNIKLCFQLSTFYSKKE-FGPICHYDQLPKNENWNGWTL-SDYEKLIGCRPQDLIRPI
              :  . .*    .:::*:.. *    . ::    *: * :.:  ***.**.  *
```

B

```
CHOV_P        MENFPSDFDPTKNLKASLESFEKSCQEESKYGGETHNDLEPSFLRADSPDELGVTGKTDVFYVSTIPVGLSSKEIHDLQITNIL
SMV_P         MENFPPDFDPTKSLNSCLKSFEKTCNEERIFGGENHNDLENASGGDSITTNDSEQSSSDVFIIEKIPVGLSAKEIEDRQVLNIL
CHOV_U1       MGESPINNYHFSDFEMTDSTLDLSEYN--------------------------------VLEINAIPEGLQQWEIKSRQIDNIL
SMV_U1        MS-YYLEELKSNDVNMEEVPFDTTAYN--------------------------------TFEVDAIPEGLPQWEIKRRQIDNIL
              *     :    ...:    .:: :  :                                .: :. ** **   **.  *: ***

CHOV_P        NGFKQIGIDCKYLSTSNGNIKFMVEWADSDSD--SEDETETDTKKEDPTAPEESDDEYNFEDV-TLSTTTYQFLQNLSNEIEKE
SMV_P         RGFQQIGIECQFTSRNDDHLKFTVIWPDDSSSEISNNETLQNDKREEKYENENNDSDDEIQSAPEIDLDTFNFLKGYSEEYVKL
CHOV_U1       YALAQLGIRCDYLVDDVDSIKFKLDFDPPLYS--YERDTDDSFIYFD--EPDDLDDLFTLIDDEEFW-----------------
SMV_U1        GALSQLGVRCDYLTDDVNSIRFYITLDPQLYS--YEVDPEDNFIIFENEQSEDEDEFLCFIEEWDF------------------
               .: *:*: *.:   . . ::* :       .   : :.  .    :    :: *.   : .   :

CHOV_P        SEITDLQDYNPRPERFLQDLKQGFNLPSIEGGHVHICLDDLTLDVSKIRHLNFPEEYDVREYCFLMLSKYSDDMELFSMVDWD
SMV_P         NQENILPQIDQKTLMFLRDLENGFELRSIEGGYVNIHLGLLKTPIDKIKKIKYPEVYDVKEYCFKMLSEDPTDIELFSMVDWD
CHOV_U1       -----------------------------------------------------------------------------------
SMV_U1        -----------------------------------------------------------------------------------
```

C

```
CURV_U3       MKNTMEAKAVVAGILLLSSLILFPTL---DAQALTILKRTCGDGMMKRVPCKLMDSVENGISARGSCLIFCSSKKKIKGKDGRM
IRIRV_U3      MKKTMGAKGRLACIFIG--MVFFSYLRESDQQGFTYIKRTCGDKMMRVIPCNLMDSVKTLTGPKGKCLVFCSSKRKIKGRDGKQ
CURV_G        M----DLVR-FSIALS---VFLCYGTPPSQGQAIVSIKDSCEAKSAPWIPCEKFDYVKNATGSGIKCWIFCSRSGFYSK-TG-R
IRIRV_G       MKKTMETIKNFILILS-----VAVQLDTGHGMEFALIRNTCDTRSMKQVPCQHLISVRESTGPKGKCLIFCAKEKRRWA-EG-V
                        .   :      .       .   :. :: :*       :**: :  *.   ..  .* :**: .       *

CURV_U3       RNLCLTGDPRADEIIKCREQILKKKTEI
IRIRV_U3      RDLCLTGDPRSDEVMKCKDKISGTKSP-
CURV_G        FIRCIQGDPEAKYIKSCRRQIEKRGKEK................................................
IRIRV_G       ESQCLQGDPTSPQVVKCKERIEK-GLER................................................
                 *: *** :  : .*: :*
```
